# Uptake of circulating extracellular vesicles from rectal cancer patients and differential responses by human monocyte cultures

**DOI:** 10.1002/2211-5463.13098

**Published:** 2021-02-08

**Authors:** Tonje Bjørnetrø, Lilly Alice Steffensen, Beate Vestad, Berit Sletbakk Brusletto, Ole Kristoffer Olstad, Anne‐Marie Trøseid, Hans Christian Dalsbotten Aass, Kari Bente Foss Haug, Alicia Llorente, Stig Ove Bøe, Anna Lång, Rampradeep Samiappan, Kathrine Røe Redalen, Reidun Øvstebø, Anne Hansen Ree

**Affiliations:** ^1^ Department of Oncology Akershus University Hospital Lørenskog Norway; ^2^ Institute of Clinical Medicine University of Oslo Norway; ^3^ The Blood Cell Research Group Department of Medical Biochemistry Oslo University Hospital Norway; ^4^ Department of Molecular Cell Biology Institute of Cancer Research Oslo University Hospital Norway; ^5^ Department of Medical Biochemistry Oslo University Hospital Norway; ^6^ Department of Bioscience and Nutrition Karolinska Institutet Huddinge Sweden; ^7^ Department of Physics Norwegian University of Science and Technology Trondheim Norway

**Keywords:** colorectal cancer, extracellular vesicles, inflammation, monocytes, tumor microenvironment

## Abstract

Extracellular vesicles (EVs) released by tumor cells can directly or indirectly modulate the phenotype and function of the immune cells of the microenvironment locally or at distant sites. The uptake of circulating EVs and the responses by human monocytes *in vitro* may provide new insights into the underlying biology of the invasive and metastatic processes in cancer. Although a mixed population of vesicles is obtained with most isolation techniques, we predominantly isolated exosomes (small EVs) and microvesicles (medium EVs) from the SW480 colorectal cancer cell line (established from a primary adenocarcinoma of the colon) by sequential centrifugation and ultrafiltration, and plasma EVs were prepared from 22 patients with rectal adenoma polyps or invasive adenocarcinoma by size‐exclusion chromatography. The EVs were thoroughly characterized. The uptake of SW480 EVs was analyzed, and small SW480 EVs were observed to be more potent than medium SW480 EVs in inducing monocyte secretion of cytokines. The plasma EVs were also internalized by monocytes; however, their cytokine‐releasing potency was lower than that of the cell line‐derived vesicles. The transcriptional changes in the monocytes highlighted differences between adenoma and adenocarcinoma patient EVs in their ability to regulate biological functions, whereas the most intriguing changes were found in monocytes receiving EVs from patients with metastatic compared with localized cancer.

## Graphical abstract

Plasma EVs may mediate the interplay between the tumor and patient’s immune responses in cancer dissemination. Isolated plasma EVs from rectal cancer patients were internalized by cultured human monocytes and caused cytokine release. Transcriptional responses associated with cancer progression were stronger and had higher biological complexity in monocytes given EVs from patients with metastasis than from patients with localized cancer.

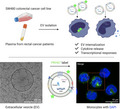

**Abbreviations**
AP_EV_: EVs from patients with noninvasive adenoma polyps
CRC: colorectal cancer
cryo‐EM: cryo‐electron microscopy
DAPI: 4',6‐diamidino‐2‐phenylindole
*EMD*: emerin
EVs: extracellular vesicles
IL‐1β: interleukin‐1β
IL‐6: interleukin‐6
IL‐8: interleukin‐8
IP‐10: interferon gamma‐induced protein 10
IPA: Ingenuity® Pathway Analysis
local‐RC_EV_: EVs from rectal cancer patients with localized disease (no metastasis) at the time of study enrollment
MCP‐1: monocyte chemoattractant protein‐1
met‐RCEV: EVs from rectal cancer patients with metastatic disease at the time of study enrollment
MIP‐1β: macrophage inflammatory protein‐1β
NTA: nanoparticle tracking analysis
PBS: phosphate‐buffered saline
PI: propidium iodide
*PRKCD*: protein kinase C, delta
RC_EV_: EVs from patients with invasive rectal cancer
SW480_EV‐S_: SW480 cell line small EVs
SW480_EV‐M_: SW480 cell line medium EVs
TME: tumor microenvironment
TNF‐α: tumor necrosis factor‐α
*TRIM28*: tripartite motif‐containing 28

Tumor cells secrete a large number of extracellular vesicles (EVs) of different intracellular origins, such as exosomes and microvesicles. The EVs carry a range of functionally active components, proteins, nucleic acids, and others, dependent on the cell type and state [1]. EVs play an important role in cell‐to‐cell communication and influence the recipient cells by interacting with cell surface receptors or by transferring their contents of bioactive molecules upon internalization [2]. EVs are involved in the regulation of vascular and epithelial barrier functions in inflamed intestines and in trafficking and activity of resident and recruited immune cells [3].

Cancer cells shape their tumor microenvironment (TME) by communicating with the surrounding stromal cells, including immune‐modulating cell types. It is now acknowledged that inflammation is of importance in cancer initiation and progression [4]. The development of invasive colorectal cancer (CRC) in the normal mucosal lining of the bowel is often a stepwise process via adenoma polyps, with genetic and epigenetic changes as early events [5]. Importantly, the TME immune cell types, functional orientation, density, and localization can determine the outcome for CRC patients [6, 7]. Tumor‐associated macrophages, originating from circulating monocytes, are an abundant component of infiltrating cells in the dynamic TME and important in these processes [8, 9]. CRC‐derived EVs can act on circulating monocytes and tumor‐associated macrophages, affecting the cellular phenotype, polarization, and activity, and thereby modulating responses related to inflammation, tumor growth, and metastasis [10]. Moreover, the local inflammatory mediators play an important role in the induction of a systemic host immune response [11]. Clinically, high blood levels of inflammatory factors are associated with increased risk of death from CRC [12, 13].

Rectal cancer typically presents as heterogeneous primary tumor manifestations within the pelvic cavity, and approximately a third of cases will have metastatic disease at presentation [14]. Assessing transcriptional changes and cytokines secreted from *in vitro* monocytes stimulated with CRC cell line and cancer patient plasma EVs may help understanding their role in tumor development, particularly mechanisms involved in the transition to invasive disease, and the metastatic process. Within this frame of reference, we hypothesized that tumor‐derived EVs might be mediators of immune and inflammatory responses that are essential for rectal cancer progression. We isolated and characterized small EVs and medium EVs from a CRC cell line and plasma EVs from patients with rectal adenoma polyps or invasive adenocarcinoma, which were subsequently incubated with human primary monocytes in order to study the uptake and elicited functional responses. All EV specimens were internalized and caused cytokine‐releasing activity, whereas the most intriguing changes were found in the transcriptional differences in monocytes receiving EVs from patients with metastatic cancer.

## Materials and methods

### Ethics approval and consent to participate

The prospective biomarker study OxyTarget (NCT01816607) in rectal cancer was approved by the Institutional Review Board and Regional Committee for Medical and Health Research Ethics of South‐East Norway (reference number REK 2013/152) and conducted in accordance with the Helsinki Declaration. Written informed consent was required to participate. The monocytes were from healthy blood donors (biobank material access number 908 at Oslo University Hospital), who had provided written consent for blood products to be used for research purposes. The material was used in accordance with the approval under REK 2013/152.

### Health and safety

We confirm that all mandatory laboratory health and safety procedures have been complied within the course of conducting any experimental work reported in this paper.

### CRC cell line and culture conditions

The human colorectal adenocarcinoma SW480 [SW480] (ATCC® CCL228™) cell line, established from a primary adenocarcinoma of the colon from a patient with Dukes' type B disease (purchased from American Type Culture Collection, Manassas, VA, USA, free of mycoplasma), was grown in RPMI medium (Gibco by Life Technologies, Paisley, UK) supplemented with 10% FBS (Lonza, Verviers, Belgium) and 1% v/v penicillin (10 000 U)/streptomycin (10 mg·mL^−1^) (Sigma‐Aldrich, Saint Louis, MO, USA). CountessII FL (Thermo Fisher Scientific, Waltham, MA, USA) was used to determine the viability and cell number. The cells were cultured at 37 °C in a 5% CO_2_‐humidified environment.

The cells were purchased in 2015 and stored at −150 °C, and the current experiments were performed October 2016‐July 2017. Cells from passage 2 were used and kept for a maximum of 6 weeks. Cell authentication (STR DNA profile) was generated by the cell bank using promega powerplex System^2^ and genemapper Software (Thermo Fisher Scientific).

### Patient and healthy blood donor materials

Citrate plasma was collected at the time of patient enrollment (patient characteristics given in Table S1). The samples were prepared by centrifugation at 2000 ***g*** for 10 min, and aliquots were stored at −80 °C. The study cohort presented here consisted of 22 patients, 16 adenocarcinoma cases and six cases with benign adenoma polyps.

Primary monocytes were isolated from healthy blood donors by elutriation purification of EDTA whole blood samples and cryopreserved at −150 °C as previously shown [15]. The cells were thawed and resuspended in medium containing 5% exosome‐depleted FBS (Thermo Fisher Scientific). Three different donors were used in the conduct of this study: the first for the analyses of SW480‐derived EV uptake and internalization (90% purity of CD14^+^ cells); the second for the analyses of plasma‐derived EV uptake and internalization as well as SW480‐ and plasma‐derived EV‐induced cytokine and transcriptional responses by monocytes (88% purity of CD14^+^ cells); and the third for the comparison analyses of SW480‐derived EVs in monocyte cytokine secretion (88% purity of CD14^+^ cells).

### EV isolation

The choice of isolation methods was dependent on the biological material and the downstream analysis to obtain separate fractions of EVs with intact integrity and biological activity [16].

#### SW480 cell line small EVs (SW480_EV‐S_) and medium EVs (SW480_EV‐M_)

The cells were seeded into T175 flasks (Thermo Fisher Scientific). At 80% confluence, the cells were washed three times with PBS (Gibco by Life Technologies), the medium was changed to serum‐free medium, and the cells were further incubated for 24 h. SW480_EV‐S_ and SW480_EV‐M_ were isolated from the conditioned media by sequential centrifugation and ultrafiltration. Briefly, cell debris and floating cells were removed by centrifugation at 300 ***g*** for 10 min and 4500 ***g*** for 5 min, and the supernatant was stored at −80 °C. The supernatant was thawed at 37 °C, and medium EVs were isolated by centrifugation at 17 000 ***g*** (11 930 r.p.m., *k*‐factor 2112) for 30 min using a fixed‐angle Sorvall SS‐34 rotor (Kendro Laboratory Products, Newtown, CT, USA). The pellet was resolved in serum‐free medium and concentrated using Amicon Ultra‐4100 kDa Centrifugal Filter Devices (Merck Millipore, Cork, Ireland). Prior to small EV isolation, to exclude particles larger than 220 nm, the 17 000 ***g*** supernatant was gently filtered through 0.22‐μm filter (Merk Millipore). Small EVs were isolated by Centricon‐70 Plus 100 kDa Centrifugal Filter Columns (Merk Millipore) and centrifuged at 3500 ***g*** for 15 min with a washing step in PBS at 3500 ***g*** for 10 min. All centrifugations were performed at room temperature. All samples were stored at −80 °C.

#### Plasma EVs

Extracellular vesicles were isolated from 500 μL citrate plasma using qEV size‐exclusion chromatography Columns (IZON Science, Oxford, UK). The columns were equilibrated with 20 mL of 0.22‐μm‐filtered PBS/0.32% citrate (Greiner Bio‐One GmbH, Kremsmüster, Austria), and EVs were isolated according to the protocol of the vendor. Fractions of 500 μL were collected, and the eluted fraction numbers 7–10 were concentrated using Amicon Ultra‐2 10 kDa Centrifugal Filter Devices (Merck Millipore). All samples were stored at −80 °C. EV specimens from patients with invasive adenocarcinoma (i.e., rectal cancer) and noninvasive adenoma polyps were termed RC_EV_ and AP_EV_, respectively.

### EV characterization

#### Cryo‐electron microscopy (cryo‐EM) analysis

Holey carbon grids (Cu R2/2; Quantifoil Micro Tools GmbH, Grosslöbichau, Germany) were glow‐discharged in a vacuum‐filled chamber for 60 s using the Balzers SCD 040 sputter coater. The EV samples (3.5 μL) were applied to the grid and incubated for 60 s in 100% humidity at 22 °C using the Vitrobot Mark I (Thermo Fisher Scientific). The grids were blotted for 3 s using a 55/20‐mm filter paper (Ted Pella, Inc., Redding, CA, USA) and frozen in liquid ethane cooled to liquid nitrogen temperature. The vitrified samples were imaged using the JEOL 2100F transmission electron microscope (JEOL Ltd., Tokyo, Japan) operated at 200 keV and a 4K × 4K CCD camera (Tietz Video and Imaging Systems GmbH, Gauting, Germany) at different magnifications (25 000× and 80 000×). The defocus values used for the images ranged from −3.0 to −4.5 µm.

#### Nanoparticle tracking analysis (NTA)

The EV samples were vortexed and diluted in PBS (0.02‐μm‐filtered; Whatman Anotop™25; GE Healthcare Life Science, Buckinghamshire, UK) to be within the recommended concentration (1.0–9.0 × 10^8^ particles per mL). The samples were loaded into the NS500 instrument (Malvern, Amesbury, UK) by a syringe at a constant flow with a syringe pump speed of 20. Three 60‐s videos were captured for each sample (slide shutter 1200, slider gain 146). The videos were analyzed by nta 3.1 software (Malvern). The vesicle quantifications had an analytic variance of 2–25% [17].

#### Immunoblot analysis

Cells and EV specimens were lysed in M‐PER® Mammalian Protein Extraction Reagent supplemented with Halt™ Protease Inhibitor Cocktail and Halt™ Phosphatase Inhibitor Cocktail (all from Thermo Fisher Scientific) for 15 min on ice and centrifuged at 17 000 ***g*** for 15 min at 4 °C. The lysates were prepared in reducing and nonreducing (for analysis of tetraspanins) conditions. Proteins from cell lysates (10 μg), SW480_EV‐S_ and SW480_EV‐M_ (300 μg), and RC_EV_ (150 μg) were separated by NuPAGE Bis/Tris (Novex by Life Technologies, Carlsbad, CA, USA). The proteins were transferred by electrophoresis to Immobilon‐P membranes (Millipore Corporation, Billerica, MA, USA). The primary antibodies were anti‐CD9 (Ts9), anti‐CD63 (Ts63), anti‐CD81 (1.3.3.22) (all from Thermo Scientific), and anti‐GM130 (D6B1) XP (Cell Signaling Technology, La Jolla, CA, USA). Secondary antibodies were from Dako Denmark (Glostrup, Denmark). Peroxidase activity was visualized using SuperSignal West Dura Extended Duration Substrate (Thermo Fisher Scientific) and the ImageQuant Las 3000 system (FujiFilm, Tokyo, Japan). The experiments were performed two or three times.

### EV uptake and internalization by monocytes

#### PKH67‐labeling of EVs

The EVs were labeled using PKH67 Green Fluorescent Cell Linker Kit (Sigma‐Aldrich), based on Lässer *et al*. [18]. The dye solution was prepared by adding 1 μL of PKH67 dye per 500 μL of Dye Solution and centrifuged at 17 000 ***g*** for 5 min to remove aggregates. The EV samples (per 100 μL) were mixed with 250 μL Diluent C and 250 μL PKH67 Dye Solution and incubated on ice for 5 min with frequent vortexing. The reaction was stopped using 500 μL 1% PBS/BSA (Sigma‐Aldrich). The labeled EVs were concentrated using Amicon Ultra‐4 100 kDa Centrifugal Filter Devices and washed three times with PBS followed by centrifugation at 3000 ***g*** for 15 min, before resuspension in culture medium. All reagents were filtered with a 0.02‐μm filter before use. The labeled EVs were stored at 4 °C until use the next day.

#### EV binding and uptake

Freshly thawed monocytes (1.5 × 10^5^/well) were incubated in a 96‐well plate (Costar 3596; Corning, NY, USA) in the absence and presence of PKH67‐labeled EVs (1.3 × 10^9^ particles per mL) in RPMI‐1640 medium with 5% exosome‐depleted FBS for 4 h at 37 °C in a 5% CO_2_‐humidified environment. A PKH67‐PBS sample was included as negative control. The cells were washed with PBS, detached with 0.25% (w/v) Trypsin (Sigma‐Aldrich), and further washed in an Eppendorf tube before evaluation by flow cytometry (BD Accuri C6; BD Biosciences, San Jose, CA, USA). Median fluorescence intensity was used to report uptake, which is composed of internalized and membrane‐bound EVs, and 10 000 events were recorded per sample. Viability of monocytes was measured by incubation with anti‐CD14 (Beckman Coulter, Marseille, France), Annexin V (BD Bioscience, Oslo, Norway), and propidium iodide (PI) (Sigma‐Aldrich) prior to measurements of median fluorescence intensity.

#### Cytochalasin D treatment

Monocytes (1.5 × 10^5^/well) were preincubated for 30 min at 37 °C in RPMI‐1640 medium with 5% exosome‐depleted FBS and 10 µg·mL^−1^ of the phagocytosis inhibitor cytochalasin D (Sigma‐Aldrich) prior to a 4‐h incubation with PKH67‐labeled SW480_EV‐S_ (1.3 × 10^9^ particles per mL). Optimal inhibitor concentration was determined from dose–response experiments monitoring the inhibition of PKH67‐labeled EV uptake combined with viability measurements with quantification of apoptosis using Annexin V/PI and expression of the monocyte marker CD14.

#### EV internalization

Monocytes (2 × 10^6^/well) were incubated in the presence of PKH67‐labeled EVs ( SW480_EV‐S_: 6–7 × 10^9^ particles per mL; AP_EV_ and RC_EV_: 5–6 × 10^11^ particles per mL) for 4 h at 37 °C in a 5% CO_2_‐humidified environment in 35‐mm glass bottom MatTek dishes (P35G‐1.5‐14‐C; MatTek Corporations, Ashland, MA, USA) coated with Poly‐d‐Lysine (A‐003‐E; Merck Millipore). The cells were washed with PBS, fixed in 4% paraformaldehyde for 15–20 min at room temperature, and mounted with VECTASHIELD mounting media containing 4',6‐diamidino‐2‐phenylindole (DAPI) (H‐1200; Vector Laboratories, Burlingame, CA, USA). Analysis of EV internalization was performed using a Leica (Leica Microsystem, Wetzlar, Germany) TCS SP8 confocal microscope equipped with a 100 × 1.40 NA oil immersion lens, a UV laser, a continuous wavelength white‐light laser set at 490 nm, and transmitted light. Z‐stack images were acquired for each sample (0.3 µm between *z*‐planes). Acquired images were further processed using the fiji imagej software (ImageJ, http://imagej.net, W Rasband, National Institute of Health, Bethesda, MD, USA) [19]. A PKH67‐PBS sample was included as negative control.

### EV‐induced monocyte phenotypes

#### Incubation conditions

Monocytes (1.5 × 10^5^/well) were incubated in a 96‐well plate in the absence and presence of EVs for 4 h at 37 °C in a 5% CO_2_‐humidified environment. Cell viability was determined by CellTiter 96®AQueous One Solution Reagent (Promega, Madison, WI, USA) according to the manufacturer's protocol. EVs were given in a dose‐dependent manner (0.1 × 10^10^, 0.5 × 10^10^, and 1.0 × 10^10^ particles per mL) in an independent experiment or with a set concentration (0.3 × 10^10^ particles per mL) to monocytes from a second donor to assess the differences in response to SW480_EV‐S_ and SW480_EV‐M_. Plasma EVs were given to the monocytes as a set volume (50 µL of the concentrated EV sample) corresponding to 1.5 × 10^10^–1.9 × 10^11^ particles per mL, thus reflecting each individual patient's total EV amount in the blood. The conditioned media were vortexed and centrifuged at 10 000 ***g*** for 10 min at 4 °C before the supernatants were stored at −80 °C for multiplex cytokine analysis (Luminex, Austin, TX, USA). The monocytes were directly lysed in QIAzol lysis buffer (Qiagen, Hilden, Germany) prior to RNA isolation.

#### Multiplex immunoassay analysis

The supernatants were thawed, and 50 μL of the samples was analyzed in duplicates on the same plate using a custom‐made human cytokine 7‐plex assay (Bio‐Rad Laboratories, Hercules, CA, USA) for the simultaneous measurement of interleukin (IL)‐1β, IL‐6, IL‐8, tumor necrosis factor‐α (TNF‐α), interferon gamma‐induced protein 10 (IP‐10), macrophage inflammatory protein‐1β (MIP‐1β), and monocyte chemoattractant protein‐1 (MCP‐1). The plates were washed with the magnetic plate washer Bio‐Plex Pro Wash Station (Bio‐Rad Laboratories). The analyses were performed using Luminex IS 100 (Luminex‐Corp.) with the Bio‐Plex software version 6.0.1 (Bio‐Rad Laboratories). Intra‐percent coefficient of variation was within acceptable range for values < 8.

#### Gene expression analysis

Total RNA was isolated using miRNeasy Micro Kit (Qiagen), following the manufacturers protocol, with additional use of phase‐lock tubes (5 PRIME GmbH, Hamburg, Germany). RNA concentration (4–15 ng·μL^−1^) and quality (RIN > 8) were assessed using NanoDrop spectrophotometer (Saveen Werner, Limhamn, Sweden), and Agilent BioAnalyzer 2100 (Agilent Technologies, Santa Clara, CA, USA). Total RNA (10 ng) was subjected to the GeneChip™ WT Pico Reagent Kit (Thermo Fisher Scientific). Microarray analysis was performed using the Affymetrix Human Clariom™ S Arrays (Affymetrix, Santa Clara, CA, USA), containing more than 20 000 well‐annotated genes. Signal intensities were detected by the Hewlett Packard (Palo Alto, CA, USA) 30007G gene array scanner and processed using the Affymetrix GeneChip Command Console software. The extracted data were imported into the Partek® genomics suite ™ software (Partek Inc., Saint Louis, MO, USA), and the Robust Multichip Analysis algorithm was applied for generation of relative signal values and normalization.

For technical validation, selected differentially expressed genes were analyzed with qRT–PCR (TaqMan gene expression assays and the Applied Biosystems ViiA7 sequence detection system) in all patients (*n* = 22). Total RNA (20 ng) was reverse transcribed using SuperScript IV VILO cDNA Synthesis kit (Thermo Fisher Scientific). cDNA was diluted 1 : 10 and analyzed in 20 μL reactions (three technical replicates) using TaqMan® Fast Advanced Master Mix (Applied Biosystems, Foster City, CA, USA) and the following primers: emerin (*EMD*) Hs02560738_s1, protein kinase C, delta (*PRKCD*) Hs01090047_m1, and tripartite motif‐containing 28 (*TRIM28*) Hs00232212_m1 as target genes. The relative changes in each transcript were analyzed using the mean of alanyl aminopeptidase (*ANPEP*) Hs00174265_m1, ribosomal protein L13A (*RLP13A*) Hs04194366_g1, and ribosomal protein L30 (*PRP30*) Hs00265497 as endogenous controls and viia7 Software v1.2 (Applied Biosystems). Pearson correlation was computed using graphpad prism 8.3.0 (GraphPad Software, San Diego, CA, USA).

#### Statistical analysis and functional annotation of array data

Following quality control and pre‐processing, the data were log_2_‐transformed. Differential gene expression between the groups [monocytes incubated with RC_EV_ or AP_EV_, and monocytes incubated with EVs from patients with metastasis (met‐RC_EV_) or localized disease (no metastasis; local‐RC_EV_) at the time of study enrollment] was determined using a one‐way ANOVA model with *P*‐value cutoff of 0.05 and fold change cutoff of 1.2. Signal values were subjected to clustering using the Partek software. Ingenuity® Pathway Analysis (IPA; QIAGEN inc., https://www.digitalinsights.qiagen.com) core analysis was used to identify involved functions and GO annotations. In IPA, Fisher's exact test was used to calculate *P*‐values determining the probability that the given functions did not represent findings due to chance, and correction for multiple testing was accounted for, when possible, by the Benjamin–Hochberg method. *Z*‐scores were calculated by the IPA‐*z*‐score algorithm to generate predictions about the direction of change in functions, and an absolute *z*‐score ≥ 2 was considered significant.

### Other statistical analyses

The data are presented as mean ± SD, mean ± SEM, or median with range. In cell line experiments, the differences between groups were determined with two‐tailed Student’s *t*‐test. The statistical analyses were performed using ibm spss Statistics (IBM, Armonk, NY, USA) for Mac v25 and graphpad prism 8.3.0, and *P*‐values < 0.05 were considered significant.

## Results

### EV characteristics

**Fig. 1:**
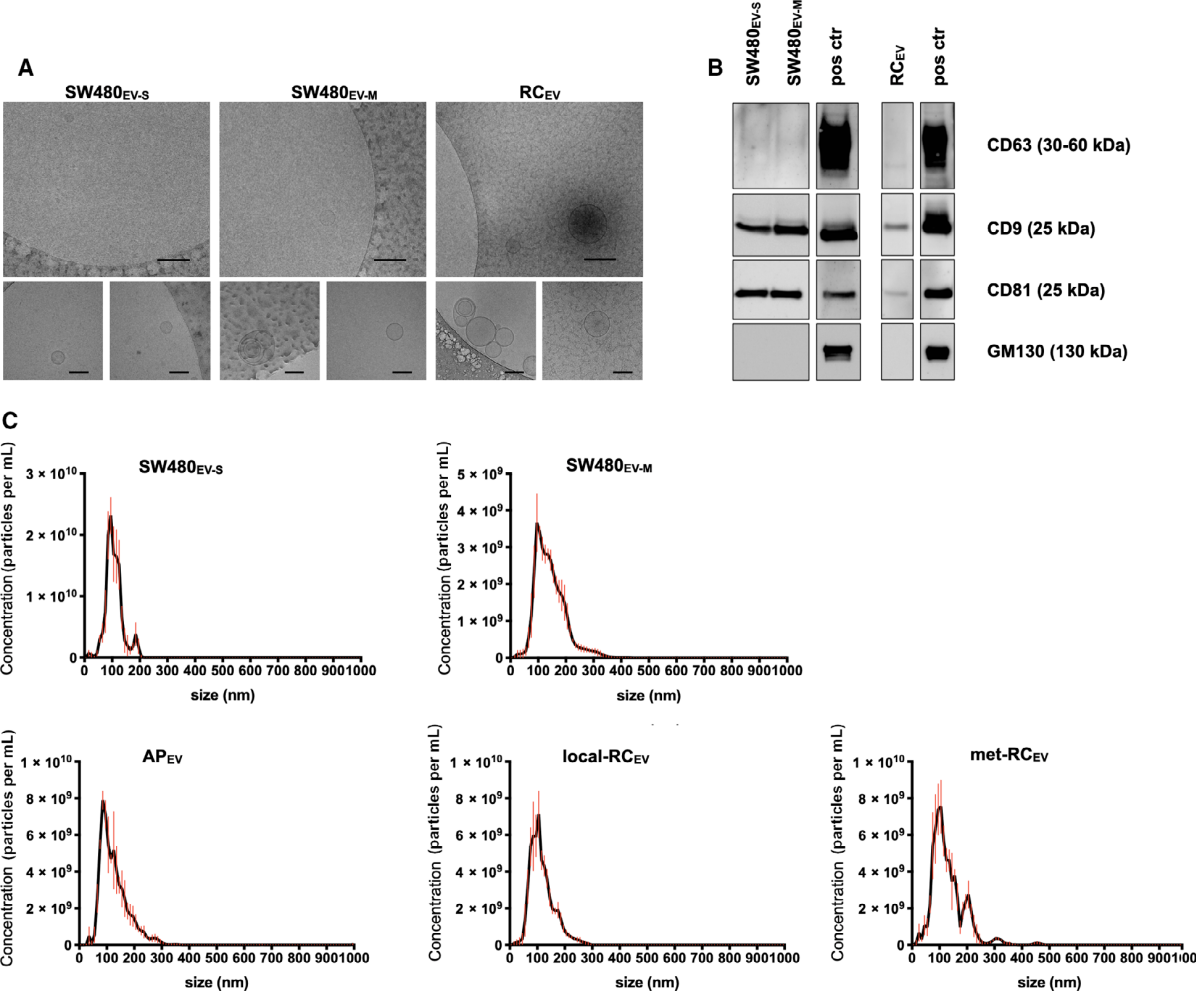
Characteristics of SW480‐derived and plasma EVs. (A) Cryo‐electron microscopy images of small EVs (SW480_EV‐S_) and medium EVs (SW480_EV‐M_) from the CRC SW480 cell line and plasma EVs from a patient with localized rectal adenocarcinoma (RC_EV_). Upper panel 25 000×, scale bars are 200 nm. Lower panel 80 000×, scale bars are 100 nm. (B) Immunoblot images of CD63, CD9, CD81, and GM130 protein expression. The samples were applied to different gels because of specific running requirements such as nonreducing conditions and overlapping molecular weight for the tetraspanins. A whole‐cell lysate of the CRC LoVo cell line was included as positive control (pos ctr). (C) Nanoparticle tracking histograms of SW480_EV‐S_, SW480_EV‐M_, plasma EVs from a patient with rectal adenoma polyp (AP_EV_), localized rectal adenocarcinoma (local‐RC_EV_) or metastatic disease (met‐RC_EV_). The data represent the mean ± standard error of the three videos from each sample. The data shown are representative for the groups.

Features of isolated SW480_EV‐S_, SW480_EV‐M_, AP_EV_, and RC_EV_ were characterized by several methods. Cryo‐EM revealed a visible membrane bilayer for SW480_EV‐S_, SW480_EV‐M_, and RC_EV_; while the two former had a diameter size in the range of 30–90 nm and 60–200 nm (*n* = 1), respectively, the patient samples showed a heterogeneous population of mostly 30–100 nm‐sized EVs but some > 100 nm (*n* = 1) (Fig. 1A). Immunoblot analysis confirmed expression of the EV‐enriched proteins CD63, CD9, and CD81 and the absence of the Golgi apparatus marker GM130 (often used as control for cellular contamination of EV samples) by SW480_EV‐S_, SW480_EV‐M_, and RC_EV_ (Fig. 1B). Further measurements by NTA showed a mode size of 90.9 ± 8.30 nm (mean ± SEM; *n* = 3) for SW480_EV‐S_, 117 ± 17.9 nm (mean ± SEM; *n* = 3) for SW480_EV‐M_, 91.30 ± 4.56 nm (mean ± SEM; *n* = 6) for AP_EV_, and 92.6 ± 3.90 nm (mean ± SEM; *n* = 16) for RC_EV_. No significant difference in plasma EV concentration (particles per mL) was found between AP_EV_ (2.30 × 10^11^±3.96 × 10^10^; mean ± SEM) and RC_EV_ (2.09 × 10^11^±3.44 × 10^10^; mean ± SEM), with a median of 2.12 × 10^11^ (range, 5.52 × 10^10^–5.50 × 10^11^) particles per mL for all patients (Fig. 1C).

### EV uptake and internalization by monocytes

**Fig. 2:**
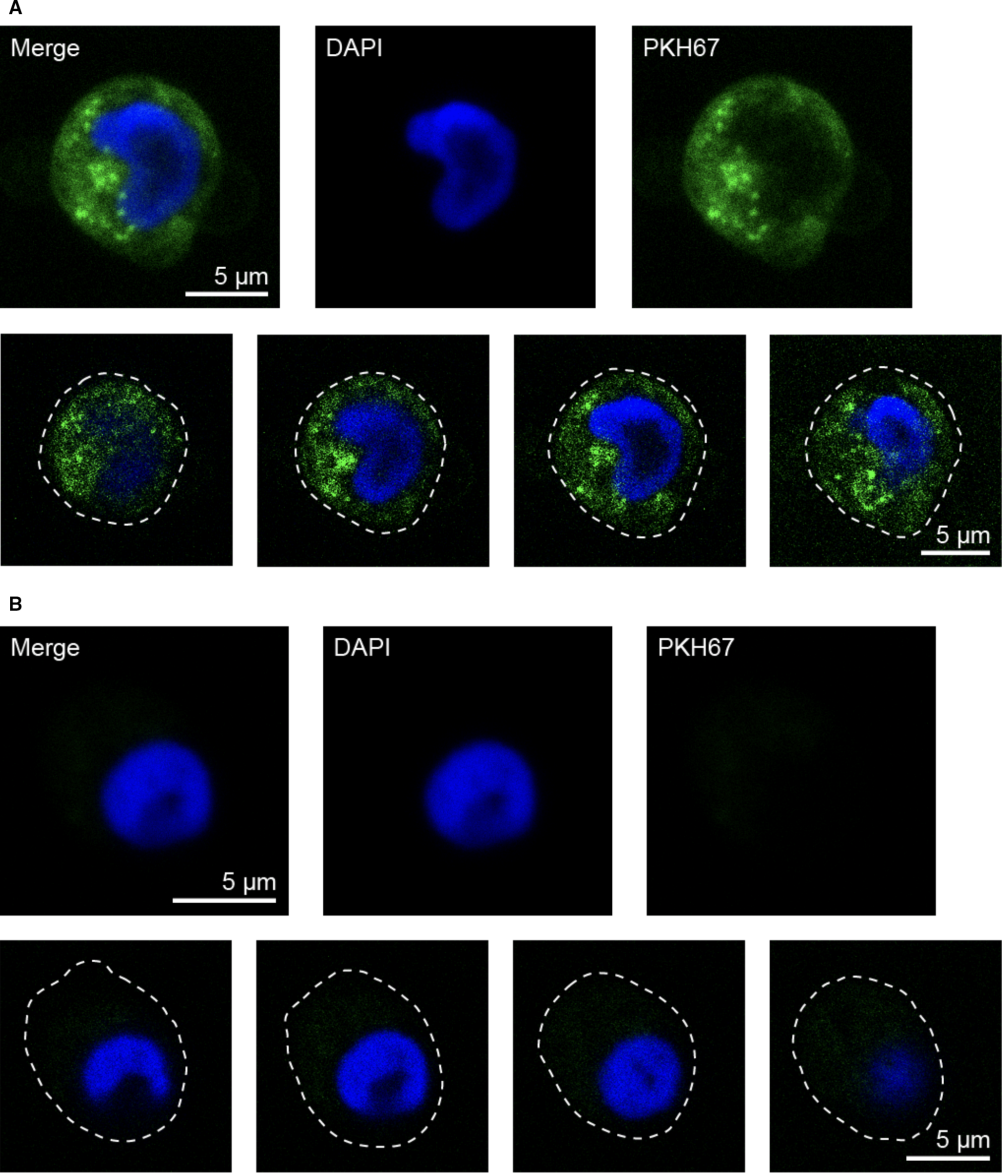
Internalization of SW480‐derived small EVs by human primary monocytes. (A) Confocal microscopy images of monocytes incubated with PKH67‐labeled EVs (green). (B) Corresponding images of monocytes incubated with PKH67‐labeled PBS as negative control. For all panels, monocytes were fixed and mounted with the nuclear stain DAPI; blue. Upper panels show average intensity z‐projections of a randomly selected monocyte. Lower panels display single *z*‐planes spanning the cell in the upper panel. White dashed lines mark the plasma membrane. Scale bars are 5 µm.

**Fig. 3:**
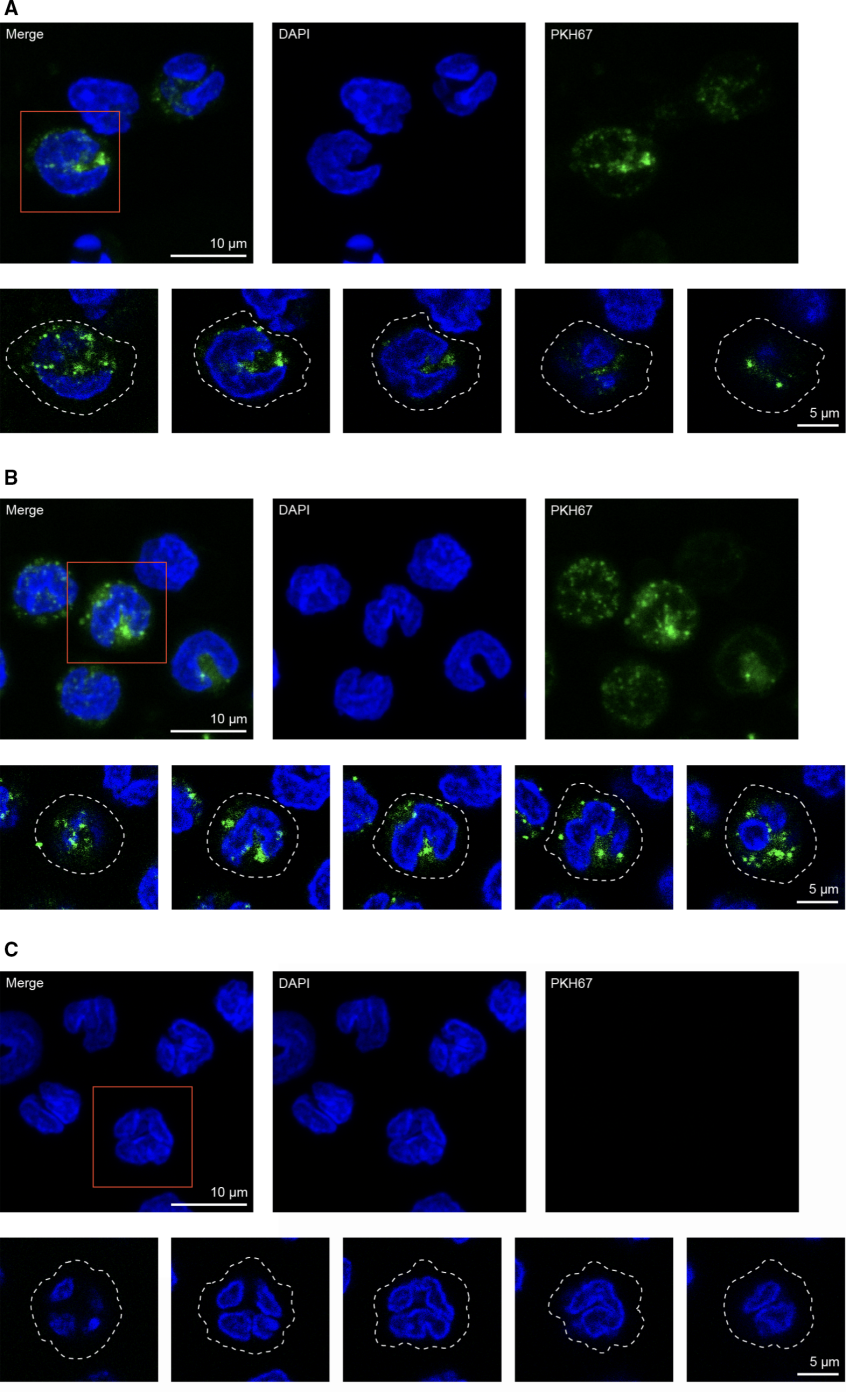
Internalization of plasma EVs by human primary monocytes. (A) Confocal microscopy images of monocytes incubated with PKH67‐labeled EVs (green) from a patient with rectal adenocarcinoma. (B) Corresponding images of monocytes incubated with PKH67‐labeled EVs (green) from a patient with a rectal adenoma polyp. (C) Corresponding images of monocytes incubated with PKH67‐labeled PBS as negative control. For all panels, monocytes were fixed and mounted with the nuclear stain DAPI; blue. Upper panels show average intensity z‐projections of randomly selected monocytes. Scale bars are 10 μm. Each red square denotes the cell shown in the corresponding lower panels, which display single *z*‐planes spanning the denoted monocytes. White dashed lines mark the plasma membrane. Scale bars are 5 μm.

Flow cytometry analysis showed enhancement of ~ 10‐ to 20‐fold in median fluorescent intensity in monocytes after 4 h of incubation with PKH67‐labeled SW480‐derived small and medium EVs (*n* = 3), and plasma EVs (*n* = 1) (Fig. S1a‐c). The internalization of SW480_EV‐S_ was further studied by confocal microscopy after incubation with PKH67‐labeled vesicles, which were clearly detected in the monocyte cytoplasm (Figs 2 and S2). Confocal microscopy analysis of PKH67‐labeled plasma EVs also showed internalization and cytoplasmic distribution (Figs 3 and S3). Each experiment was performed once.

Because plasma EV preparations contain a complex mixture of various EV types secreted from many types of blood cells and tissues, SW480_EV‐S_ was chosen for analyzing the contribution of phagocytosis to the endocytic pathways used by the primary monocytes for EV uptake. The toxicity of the phagocytosis inhibitor cytochalasin D (10 µg·mL^−1^) on monocytes was determined as negligible (data not shown). The amount of PKH67‐labeled SW480_EV‐S_ associated with monocytes was measured by flow cytometry in the absence and presence of cytochalasin D and shown to be reduced by an average of 36% by cytochalasin D (*n* = 3) (Fig. S1d).

### EV‐induced monocyte phenotypes

**Fig. 4:**
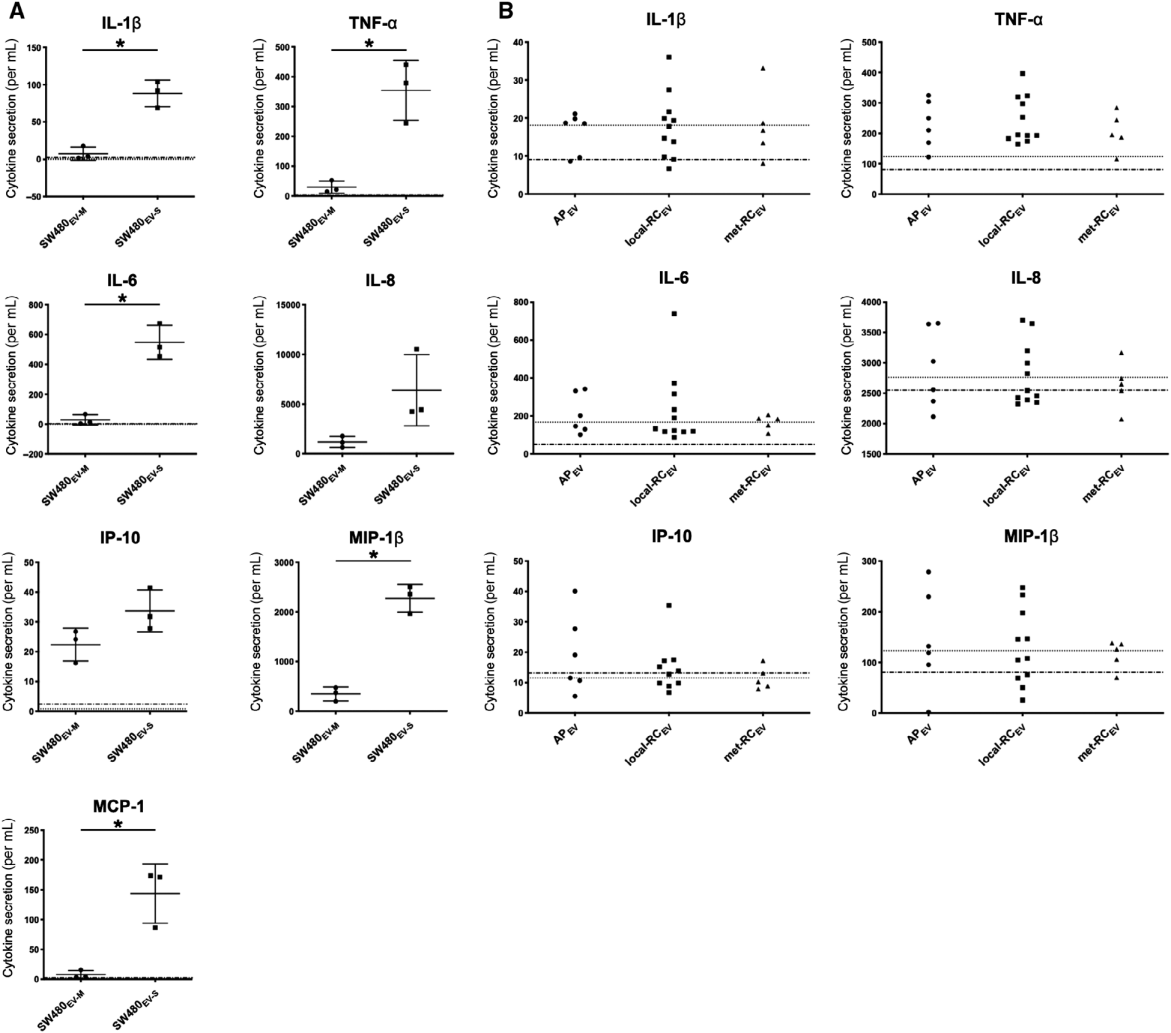
Cytokine secretion by human primary monocytes given SW480‐derived and plasma EVs. (A) Multiprotein (Luminex) assay results following incubation with CRC SW480 cell line medium EVs (SW480_EV‐M_) or small EVs (SW480_EV‐S_). The data represent the mean ± standard deviation of three independent experiments; * two‐tailed Student’s *t*‐test *P* < 0.05. (B) Luminex assay results following incubation with plasma EVs from patients with either a rectal adenoma polyp (AP_EV_) (*n* = 6), localized adenocarcinoma (local‐RC_EV_) (*n* = 11), or metastatic disease (met‐RC_EV_) (*n* = 5). Cytokine secretion by control monocytes given citrate PBS (dotted line) or serum‐free cell culture medium (stippled line).

Since all the investigated EV populations were able to enter the monocytes, the question arose whether immune or inflammatory responses might be elicited. First, following the 4‐h EV incubation, monocyte supernatants were analyzed for seven cytokines selected on the basis of a previous screening of monocyte responses to SW480_EV‐S_ (unpublished data). A strong and dose‐dependent secretion was observed for IL‐1β, IL‐6, IL‐8, MIP‐1β, TNF‐α, and MCP‐1 in particular, whereas IP‐10 showed a moderate increase (Fig. S4). In general, SW480_EV‐S_ was more potent than SW480_EV‐M_ (Fig. 4A). The monocyte viability was not affected by EVs (data not shown). The monocytes incubated with AP_EV_, local‐RC_EV_, or met‐RC_EV_ showed a more moderate increase in the secretion responses, several even below the controls (Fig. 4B). The MCP‐1 measures were not reliable because of technical challenges.

**Fig. 5:**
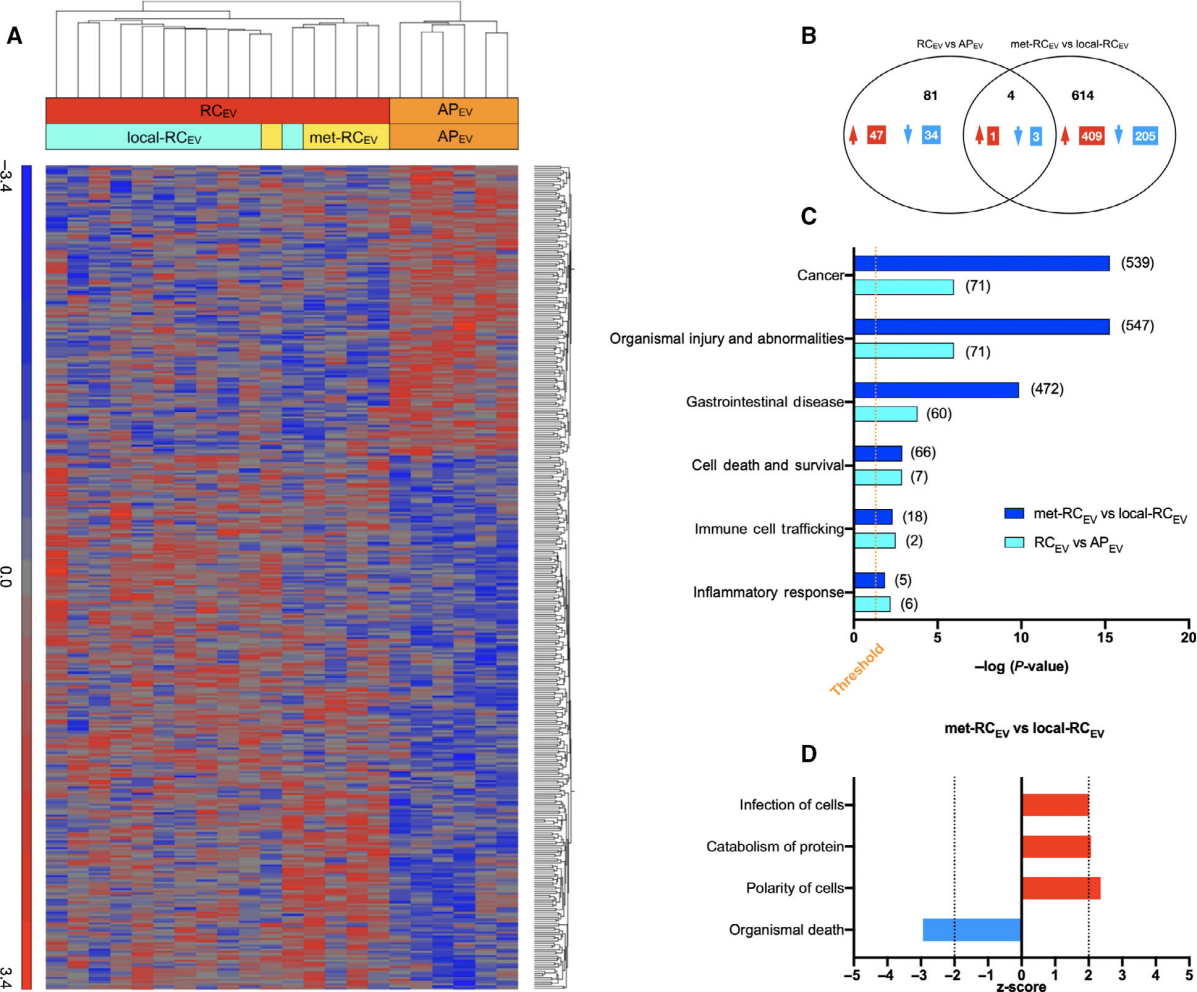
Gene expression in human primary monocytes given plasma EVs and selected high‐level functional categories. (A) Unsupervised hierarchical clustering of differentially expressed genes following incubation with plasma EVs from patients with rectal adenoma polyps (AP_EV_; *n* = 6) or invasive adenocarcinoma (RC_EV_) who had either localized cancer (local‐RC_EV_; *n* = 11) or metastatic disease (met‐RC_EV_; *n* = 5). In the heat map, each column represents one patient and each row one transcript. Red colors indicate upregulation and blue colors downregulation of transcripts. (B) The number of significantly upregulated (red) and downregulated (blue) genes on comparison of the indicated groups. The data represent a one‐way ANOVA *P*‐value cutoff of 0.05 and a fold change cutoff of 1.2. (C) Shared diseases and functions among the indicated groups, as predicted by IPA. The –log significance level is shown along the *x*‐axis. The orange dotted line denotes the cutoff for significance, corresponding to Fisher's exact test *P*‐value 0.05. The figure in bracket behind each bar is the number of transcripts in the dataset involved in the given category. (D) Predicted activation state of function annotations significantly regulated in met‐RC_EV_ compared with local‐RC_EV_ by IPA, corresponding to Fisher's exact test *P*‐value 0.05. The *z*‐score is shown along the x‐axis, and a positive (red) or negative (blue) *z*‐score indicates that the function is predicted to increase or decrease. The black dotted lines denote the cutoff for significance, corresponding to ≥ 2 and ≤ −2.

Interestingly, incubation with AP_EV_, local‐RC_EV_, and met‐RC_EV_ evoked clearly distinguishable transcriptional responses by monocytes (Fig. 5A). Using a fold change cutoff of 1.2, RC_EV_ caused differential expression of 85 genes (48 upregulated, 37 downregulated) when compared to AP_EV_ (Fig. 5B). When comparing met‐RC_EV_ and local‐RC_EV_, the vesicles from patients with metastatic disease led to 618 differentially expressed genes (410 upregulated and 208 downregulated) (Fig. 5B). Four transcripts were found to be jointly regulated in monocytes in the comparisons of RC_EV_ vs AP_EV_ and met‐RC_EV_ vs local‐RC_EV_ (Fig. 5B), and involved in protein binding, apoptotic mitochondrial changes, immune cell signaling, and cell growth, among other biological processes (Table S2). The top 10 up‐ and downregulated transcripts in monocytes incubated with EVs were involved in a range of metabolic and immune system processes (Table S3).

Among the five most discriminating RNA profile‐defined functions, when applying the IPA software, were cell morphology, cellular assembly and organization, and organ morphology for AP_EV_ vs RC_EV_, and endocrine system disorders, gastrointestinal disease, and hematological disease for met‐RC_EV_ vs local‐RC_EV_ (Table 1; the transcript IDs assigned to the disease and disorder categories are provided in Table S4). Associations with the categories cancer and organismal injury and abnormalities were found for both comparisons (Tables 1 and S4). All patients with disseminated disease had liver metastasis (one had additional lung and bone metastases). For the met‐RC_EV_ vs local‐RC_EV_ comparison, a higher complexity and significance were found for transcripts involved in the categories cancer, organismal injury and abnormalities, and gastrointestinal disease (Fig. 5C; the transcript IDs assigned to the disease and function categories are provided in Tables S4,S5). However, the categories cell death and survival, immune cell trafficking, and inflammatory response were involved to a similar significance level in the various patient groups (Fig. 5C and Table S5). Predictions on the direction of change for the functions were only applicable for the dataset of met‐RC_EV_ vs local‐RC_EV_. A negative or positive *z*‐score value indicates that the function was predicted to decrease or increase in monocytes receiving EVs from patients with metastatic disease compared to patients with localized disease. Organismal death (*z*‐score of −3.020, *P* = 0.014) was among the functions that were predicted to significantly decrease, whereas polarity of cells (*z*‐score of 2.433, *P* = 0.014), catabolism of protein (*z*‐score of 2.149, *P* = 0.015), and infections of cells (*z*‐score of 2.085, *P* = 0.0078) were predicted to significantly increase (Fig. 5D).

**Table 1:** Gene expression in human primary monocytes given plasma EVs. Comparison of monocytes incubated with plasma EVs from patients with rectal adenoma polyps (AP_EV_) or invasive adenocarcinoma (RC_EV_) who had either localized cancer (local‐RC_EV_) or metastatic disease (met‐RC_EV_). The data represent a *P*‐value cutoff of 0.05 and fold change cutoff of 1.2. Upper table shows the number of differentially expressed transcripts and analysis‐ready molecules across observations eligible for IPA software analysis. Lower tables shows top five diseases and disorders in the core analysis, as defined by IPA, assigned to the transcriptional changes using Fisher's exact test *P*‐values and Benjamin–Hochberg (B‐H) multiple testing correction *P*‐values.

	RC_EV_ vs AP_EV_	met‐RC_EV_ vs local‐RC_EV_	
Differentially expressed transcripts	85	618	
Analysis‐ready molecules in IPA	73	588	
Top 5 Molecular and cellular functions^a^	No. of transcripts	*P*‐value range	B‐H *P*‐value range
RC_EV_ vs AP_EV_			
Cancer	71	1.05E‐06–3.92E‐02	1.83E‐03–1.35E‐01
Organismal injury and abnormalities	71	1.05E‐06–3.92E‐02	1.83E‐03–1.35E‐01
Cell morphology	12	3.05E‐05–3.90E‐02	1.78E‐02–1.35E‐01
Cellular assembly and organization	15	3.05E‐05–3.90E‐02	1.78E‐02–1.35E‐01
Organ morphology	12	3.05E‐05–3.79E‐02	1.78E‐02–1.34E‐01
met‐RC_EV_ vs local‐RC_EV_			
Cancer	539	5.21E‐16–2.59E‐02	2.41E‐12–2.05E‐01
Organismal injury and abnormalities	547	5.21E‐16–2.59E‐02	2.41E‐12–2.05E‐01
Endocrine system disorders	425	3.05E‐12–2.59E‐02	2.53E‐09–2.05E‐01
Gastrointestinal disease	472	1.37E‐10–2.59E‐02	6.68E‐08–2.05E‐01
Hematological disease	162	5.06E‐05–2.59E‐02	1.35E‐02–2.05E‐01

^a^ Functional core analysis. Gene expression differences in monocytes induced by the indicated groups. Eligible transcripts for IPA analysis were 15 052 of a total 16 598.

Technical validation of the gene expression array by qRT–PCR resulted in the following correlations: *EMD* (Pearson *r* = 0.583, *P* = 0.0044), *PRKCD* (Pearson *r* = 0.555, *P* = 0.0073), and *TRIM28* (Pearson *r* = 0.405, *P* = 0.061).

## Discussion

Presuming tumor‐derived EVs are critical in shaping an inflammatory TME and thereby facilitate disease dissemination to distant organs, we assessed the uptake and functional responses by human monocyte cultures of CRC cell line small EVs (SW480_EV‐S_) and medium EVs (SW480_EV‐M_) as well as plasma EVs from patients with rectal adenoma polyps (AP_EV_) and localized (local‐RC_EV_) or metastatic (met‐RC_EV_) adenocarcinoma. All vesicle types were efficiently internalized by monocytes. SW480_EV‐S_ was more potent in inducing cytokine responses than SW480_EV‐M_ on direct comparison. The AP_EV_, local‐RC_EV_, and met‐RC_EV_ evoked clearly distinguishable transcriptional responses with the most compelling evidence of biologically relevant EV‐directed pathophysiology for patients with metastatic cancer.

Circulating EV levels have been reported as elevated in CRC patients compared with healthy subjects [20, 21]. There were no differences in the quantity of plasma EVs in our small cohort of patients with rectal cancer or adenoma polyps. The cultured monocytes internalized all of the investigated EV types. The uptake of SW480_EV‐S_ in monocytes was partly inhibited by cytochalasin D, indicating that phagocytosis was involved but was not the only uptake mechanism [22, 23]. This is in accordance with previous reports showing that the uptake of EVs by monocytes and macrophages is partly dependent on dynamin‐dependent endocytic pathways and phagocytosis [24, 25]. Specificity of labeling is a limitation for EV visualization using PKH67 [26]. This lipophilic dye was not used when performing functional assays with EVs.

The functional effect of exposure of CRC‐derived EVs on monocyte/macrophage differentiation and polarization is complex and probably dependent on the cell origin of the EVs, the timing of exposure, as well as the stage of maturation of the recipient cell [10]. Early studies showed that *in vitro* stimulation of human monocytes with CRC‐EVs altered the monocyte phenotype toward antitumor activity [27, 28]. Moreover, monocytes were differentiated by CRC‐EVs into macrophages with a mixed polarization status (M1/M2) depending on contact time and vesicle cargo [24, 28]. Others have described an M2 phenotype after exposure to CRC‐EVs, promoting *in vivo* tumor growth and development of metastasis [29, 30]. Our study additionally addressed the differential cytokine release by cell line small EVs and medium EVs, and the impact of EVs isolated from the circulation in patients with rectal benign or malignant tumors. Both SW480_EV‐S_ and SW480_EV‐M_ increased the cytokine secretion by the monocytes and based on equal number of vesicles given, small EVs seemed to be more potent. This indicates that the nature of the vesicles is important, but we could not rule out that there is a balance or threshold that needs to be achieved to detect an effect or that freezing and thawing could have affected EV integrity and function of the vesicles. There is also a possibility that the EV‐associated functions are partly mediated by non‐EV‐bound components co‐isolated with the different fractions, or by a combination of both [31]. Our experiments were compared with a medium (EV‐free) control condition. A detailed investigation into the effects of EV fractions, EV‐depleted fractions, and unfractionated starting material (initial fluid) might have determined the relative contribution of each [32]. Another approach is to compare the activity of intact EVs with EV samples treated with detergents to destroy the vesicle structure [33]. Altogether, our data endorse the role of EV‐enriched preparations as profound modulators of monocyte’s biological activity.

Increased monocyte or macrophage release of TNF‐α, IL‐1β, and IP‐10 has previously been reported upon exposure of CRC cell line‐derived EVs [24, 27, 28, 34]. The cytokine levels in the circulation and tumors in CRC patients have been associated with disease progression and poor survival [13, 35, 36, 37]. SW480 is a well known and commonly used cell line for investigating CRC‐derived EVs effects on monocytes/macrophages [24, 28], and in this study, it was used to establish a reliable model system for monocyte responses of cancer‐derived EVs. In our experimental setting, AP_EV_ and RC_EV_ were less potent than EVs from SW480 cells with regard to the cytokine responses. Unexpectedly, few of the patient specimens induced secretion above the controls. We cannot exclude the possibility that EVs from a colon cancer cell line are dissimilar to circulating EVs from rectal cancer patients or that the patient‐derived EVs might stimulate the secretion of other cytokines (e.g., anti‐inflammatory cytokines) than the ones recorded in this study. It is important to note that blood harbors a great number of EVs derived from blood cells (platelets, erythrocytes, lymphocytes, granulocytes, and monocytes) in addition to possible tumor EVs [38], and the dose of cancer‐derived EVs in our study might be too low to elicit a measurable effect.

In the global analysis of transcriptional monocyte responses to EVs, the functional categories cancer, organismal injury and abnormalities, and gastrointestinal disease were more strongly induced in monocytes given plasma EVs from patients with metastatic disease than from cases with tumor confined to the pelvic cavity. In the comparison of downstream effect analysis, these categories were also shown with stronger significance in the group of patients with metastasis. Interestingly, we observed that the plasma EVs from patients with invasive cancer compared with adenoma polyps caused effects that are essential in cancer. This category had annotations such as carcinoma, vascularization of tumor, and function of mitochondria. Further along this line, additional annotations associated with plasma EVs from patients with metastatic disease were tumorigenesis of tissue, gastrointestinal carcinoma, and hematologic cancer. Many of the identified genes linked to the category gastrointestinal disease have been shown to be involved in CRC initiation and development as well as intestinal inflammation [39, 40, 41]. The categories inflammatory response, immune cell trafficking, and cell death and survival were involved to a similar significance level in the two patient comparisons. However, EVs from patients with metastatic disease were predicted to repress functions involved in organismal death by upregulation of transcripts essential in cellular development and survival, and in tumor progression *in vitro*. Some upregulated transcripts were associated with death in patients with several cancer types. IPA also predicted activation of polarity of monocytes, with transcripts regulating motility and adhesion, cell metabolism, and functions involved in infection of cells, such as viruses resembling vesicles in physical and chemical characteristics. These findings may reflect the interaction between the cancer and the detrimental immune response in patients with disseminated CRC that commonly is seen in clinical practice [42].

Our findings are in strong support of *in vitro* monocytes being a relevant model system for investigating local TME and systemic host responses to circulating EVs in rectal cancer patients. Limitations that need to be taken into consideration are the small patient cohort with nonrecorded comorbidities that might have affected the results. Each group comprised a heterogeneous patient population, and the circulating EVs were derived from individuals with the only joint and controllable feature of a rectal tumor. We also acknowledge that there could be individual differences in the effects of tumor‐derived EVs on monocytes isolated from different healthy donors. However, because of limitation of biological material (plasma volume for EV isolation) from the rectal cancer patients, it was required to focus the main experiment on monocytes from only one donor. The monocyte transcriptional differences among the groups were rather low with the fold change cutoff of 1.2; more stringent settings would have made the data ineligible for enrichment analysis by the IPA software.

In conclusion, plasma EVs from patients diagnosed with a rectal tumor were internalized by human primary monocytes cultured *in vitro* and caused responses related to the disease progression from noninvasive to invasive and disseminated cancer. At the gene expression level, the monocyte responses distinguished EVs from subjects with adenoma polyps from cases with invasive cancer that was localized within the pelvic cavity and further from those with metastatic disease. Additional studies of circulating patient EVs are still needed to understand their evident role in cancer immunity and inflammation.

## Conflict of interest

The authors declare no conflict of interest.

## Author contributions

TB, KRR, RØ, and AHR were responsible for the concept and experimental design. TB, LAS, BV, BSB, AMT, KBFH, ALl, SOB, and RØ were involved in the development of methodology. TB, LAS, BSB, OKO, HCDA, ALo, and RS performed the experiments, analyzed, and interpreted the data. TB, RØ, and AHR wrote the manuscript. All authors read and approved the manuscript.

## Supporting information


**Table S1.** Patient characteristics.
**Table S2.** Biological processes in human primary monocytes given plasma extracellular vesicles (EVs) associated with the four jointly regulated transcripts across observations. Comparison of monocytes incubated with plasma EVs from patients with rectal adenoma polyps (APEV) or invasive adenocarcinoma (RCEV) who had either localized cancer (local‐RCEV) or metastatic disease (met‐RCEV). The data represent a *P*‐value cut‐off of 0.05 and fold‐change cut‐off of 1.2. Biological processes (GO annotations), defined by Ingenuity® Pathway Analysis, assigned to the transcriptional changes by Fisher's Exact Test *P*‐values.
**Table S3.** Biological processes in human primary monocytes given plasma extracellular vesicles (EVs) associated with the top ten up‐ or down‐regulated transcripts. Comparison of monocytes incubated with plasma EVs from patients with rectal adenoma polyps (APEV) or invasive adenocarcinoma (RCEV) who had either localized cancer (local‐RCEV) or metastatic disease (met‐RCEV). The data represent a *P*‐value cut‐off of 0.05 and foldchange cut‐off of 1.2. Biological processes (GO annotations), defined by Ingenuity® Pathway Analysis, assigned to the transcriptional changes by Fisher's Exact Test *P*‐values.
**Table S4.** Transcript IDs assigned to the top five diseases and disorders in Table 1. Comparison of monocytes incubated with plasma extracellular vesicles (EVs) from patients with rectal adenoma polyps (APEV) or invasive adenocarcinoma (RCEV) who had either localized cancer (local‐RCEV) or metastatic disease (met‐RCEV). The data represent a *P*‐value cut‐off of 0.05 and fold‐change cut‐off of 1.2.
**Table S5.** Transcripts assigned to the additional diseases and functions in Figure 5c. Comparison of monocytes incubated with plasma extracellular vesicles (EVs) from patients with rectal adenoma polyps (APEV) or invasive adenocarcinoma (RCEV) who had either localized cancer (local‐RCEV) or metastatic disease (met‐RCEV). The data represent a *P*‐value cut‐off of 0.05 and fold‐change cut‐off of 1.2.
**Fig. S1.** Uptake of SW480‐derived and plasma extracellular vesicles (EVs) by human primary monocytes. (A) Representative plots in flow cytometry analysis for identification of monocytes. Cells were selected in an SSC vs FSC dotplot (gate I) and single cells were identified and gated (gate II) in a width vs SSC dotplot. Single monocytes were identified (gate III) in a FSC vs Anti‐CD14‐APC dotplot. (B) Representative plots in flow cytometry analysis of primary monocytes incubated with PKH67‐labeled EVs from the colorectal cancer cell line SW480. Monocytes were identified and gated (gate I) in an SSC vs FSC dotplot and single monocytes were identified and gated (gate II) in a width vs SSC dotplot. The fluorescence intensity of primary monocytes incubated with PKH67‐labeled EVs is shown in an overlay histogram of monocytes given PKH67‐labeled phosphate‐buffered saline (PBS) (dark blue line), SW480EV‐S (violet line), or SW480EV‐M (orange line). (C) Flow cytometry analysis of primary monocytes incubated with PKH67‐labeled EVs. The data represent the mean ± standard deviation of median fluorescence intensity from 3 independent experiments for small EVs (SW480EV‐S) and medium EVs (SW480EV‐M) from the colorectal cancer SW480 cell line and 1 experiment each for plasma EVs from patients with either rectal adenocarcinoma (RCEV) or a rectal adenoma polyp (APEV). Monocytes given PKH67‐labeled PBS were used as negative control. (D) Flow cytometry analysis of primary monocytes incubated with PKH67‐labeled SW480EV‐S in the absence (PBS) or presence of cytochalasin D (Cyt D). The data represent mean percent inhibition ± standard deviation from 3 independent experiments.
**Fig. S2.** Internalization of SW480‐derived small extracellular vesicles (EVs) by human primary monocytes. Single *z*‐planes of the monocyte shown in each lower panel of Figure 3 A and B. A Images of monocyte given PKH67‐labeled small EVs (green). B Corresponding images of monocytes incubated with PKH67‐labeled phosphate‐buffered saline as negative control. For all panels Cells were fixed and mounted with the nuclear stain 4',6‐diamidino‐2‐phenylindole (DAPI; blue). White dashed lines mark the plasma membrane, which is also shown in the transmitted light channel (grey). Scale bars are 5 μm.
**Fig. S3.** Internalization of plasma extracellular vesicles (EVs) by human primary monocytes. Single *z*‐planes of the cell shown in each lower panel of Figure 4 A‐C. (A) Images of monocytes given PKH67‐labeled EVs (green) from a patient with rectal adenocarcinoma. (B) Corresponding images of monocytes incubated with PKH67‐labeled EVs (green) from a patient with a rectal adenoma polyp. (C) Corresponding images of monocytes incubated with PKH67‐labeled phosphate‐buffered saline as negative control. For all panels Cells were fixed and mounted with the nuclear stain 4',6‐diamidino‐2‐phenylindole (DAPI; blue). White dashed lines mark the plasma membrane, which is also shown in the transmitted light channel (grey). Scale bars are 5 μm.
**Fig. S4.** Cytokine secretion by human primary monocytes given SW480‐derived extracellular vesicles. Multiprotein (Luminex) assay results following incubation with colorectal cancer SW480 cell line medium EVs (SW480EV‐M) or small EVs (SW480EV‐S) in the indicated numbers (particles/mL). Control monocytes were given serum‐free RPMI medium. The data represent an experiment performed once.

## Data Availability

Request to inspect and analyze the data that underlie the results in this article, including the RNA array data, should be directed to the corresponding author, and access will be provided in accordance with the General Data Protection Regulation of the European Union.
