# Supplementary material for: Uptake of circulating extracellular vesicles from rectal cancer patients and differential responses by human monocyte cultures
Source: FEBS Open Bio. 2021 Feb 8;11(3):724–40. doi: 10.1002/2211-5463.13098 (PMC7931235; doi:10.1002/2211-5463.13098)
Supplement: Supplementary file 1 — Table S1. Patient characteristics. Table S2. Biological processes in human primary monocytes given plasma extracellular vesicles (EVs) associated with the four jointly regulated transcripts across observations. Comparison of monocytes incubated with plasma EVs from patients with rectal adenoma polyps (APEV) or invasive adenocarcinoma (RCEV) who had either localized cancer (local‐RCEV) or metastatic disease (met‐RCEV). The data represent a P‐value cut‐off of 0.05 and fold‐change cut‐off of 1.2. Biological processes (GO annotations), defined by Ingenuity® Pathway Analysis, assigned to the transcriptional changes by Fisher's Exact Test P‐values. Table S3. Biological processes in human primary monocytes given plasma extracellular vesicles (EVs) associated with the top ten up‐ or down‐regulated transcripts. Comparison of monocytes incubated with plasma EVs from patients with rectal adenoma polyps (APEV) or invasive adenocarcinoma (RCEV) who had either localized cancer (local‐RCEV) or metastatic disease (met‐RCEV). The data represent a P‐value cut‐off of 0.05 and foldchange cut‐off of 1.2. Biological processes (GO annotations), defined by Ingenuity® Pathway Analysis, assigned to the transcriptional changes by Fisher's Exact Test P‐values. Table S4. Transcript IDs assigned to the top five diseases and disorders in Table 1. Comparison of monocytes incubated with plasma extracellular vesicles (EVs) from patients with rectal adenoma polyps (APEV) or invasive adenocarcinoma (RCEV) who had either localized cancer (local‐RCEV) or metastatic disease (met‐RCEV). The data represent a P‐value cut‐off of 0.05 and fold‐change cut‐off of 1.2. Table S5. Transcripts assigned to the additional diseases and functions in Figure 5c. Comparison of monocytes incubated with plasma extracellular vesicles (EVs) from patients with rectal adenoma polyps (APEV) or invasive adenocarcinoma (RCEV) who had either localized cancer (local‐RCEV) or metastatic disease (met‐RCEV). The data represent a P‐valu [file FEB4-11-724-s001.pdf]

Uptake of circulating extracellular vesicles from rectal cancer patients and differential responses by human monocyte cultures

Journal:	*FEBS Open Bio*
Manuscript ID	FEBSOPEN-20-0849.R2
Wiley - Manuscript type:	Research Article
Date Submitted by the Author:	13-Jan-2021

Complete List of Authors:	Bjørnetrø, Tonje; Akershus University Hospital, Department of Oncology; University of Oslo, Institute of Clinical Medicine<br>Steffensen, Lilly Alice; Oslo University Hospital, Department of Medical Biochemistry<br>Vestad, Beate; Oslo University Hospital, Department of Medical Biochemistry<br>Brusletto, Berit; Oslo University Hospital, Department of Medical Biochemistry<br>Olstad, Ole Kristoffer; Oslo University Hospital, Department of Medical Biochemistry<br>Trøseid, Anne-Marie; Oslo University Hospital, Department of Medical Biochemistry<br>Aass, Hans Christian; Oslo University Hospital, Department of Medical Biochemistry<br>Haug, Kari Bente; Oslo University Hospital, Department of Medical Biochemistry<br>Llorente, Alicia; Oslo University Hospital, Department of Molecular Cell Biology<br>Bøe, Stig Ove; Oslo University Hospital, Department of Medical Biochemistry<br>Lång, Anna; Oslo University Hospital, Department of Medical Biochemistry<br>Samiappan, Rampradeep; Karolinska Institute, Department of Bioscience and Nutrition<br>Redalen, Kathrine Røe; Norwegian University of Science and Technology, Department of Physics; Akershus University Hospital, Oncology<br>Øvstebø, Reidun ; Oslo University Hospital, Department of Medical Biochemistry<br>Ree, Anne; Akershus University Hospital, Oncology; University of Oslo, Institute of Clinical Medicine

Search Terms:	colorectal cancer, extracellular vesicles, monocytes, tumor microenvironment

Abstract:	Extracellular vesicles (EVs) released by tumor cells can directly or indirectly modulate the phenotype and function of the immune cells of the microenvironment locally or at distant sites. The uptake of circulating EVs and the responses by human monocytes *in vitro* may provide new

insights into the underlying biology of the invasive and metastatic processes in cancer. Although a mixed population of vesicles is obtained with most isolation techniques, we predominantly isolated exosomes (small EVs) and microvesicles (medium EVs) from the SW480 colorectal cancer cell line (established from a primary adenocarcinoma of the colon) by sequential centrifugation and ultrafiltration, and plasma EVs were prepared from 22 patients with rectal adenoma polyps or invasive adenocarcinoma by size-exclusion chromatography. The EVs were thoroughly characterized. The uptake of SW480 EVs was analyzed, and small SW480 EVs were observed to be more potent than medium SW480 EVs in inducing monocyte secretion of cytokines. The plasma EVs were also internalized by monocytes; however, their cytokine-releasing potency was lower than that of the cell line-derived vesicles. The transcriptional changes in the monocytes highlighted differences between adenoma and adenocarcinoma patient EVs in their ability to regulate biological functions, whereas the most intriguing changes were found in monocytes receiving EVs from patients with metastatic compared to localized cancer.

SCHOLARONE™
Manuscripts

Uptake of circulating extracellular vesicles from rectal cancer patients and differential responses by human monocyte cultures

Tonje Bjørnetrø[1,2], Lilly Alice Steffensen[3], Beate Vestad[3], Berit Sletbakk Brusletto[3], Ole Kristoffer Olstad[3], Anne-Marie Trøseid[3], Hans Christian Dalsbotten Aass[3], Kari Bente Foss Haug[3], Alicia Llorente[4], Stig Ove Bøe[5], Anna Lång[5], Rampradeep Samiappan[6], Kathrine Røe Redalen[1,7], Reidun Øvstebø[3,*] and Anne Hansen Ree[1,2,*].

[1] Department of Oncology, Akershus University Hospital, Lørenskog, Norway; [2] Institute of Clinical Medicine, University of Oslo, Oslo, Norway; [3] The Blood Cell Research Group, Department of Medical Biochemistry, Oslo University Hospital, Oslo, Norway; [4] Department of Molecular Cell Biology, Institute of Cancer Research, Oslo University Hospital, Oslo, Norway; [5] Department of Medical Biochemistry, Oslo University Hospital, Oslo, Norway, [6] Department of Bioscience and Nutrition, Karolinska Institutet, Huddinge, Sweden; [7] Department of Physics, Norwegian University of Science and Technology, Trondheim, Norway. * Shared last authorship

Corresponding author: Tonje Bjørnetrø, Department of Oncology, Akershus University Hospital, P.O. Box 1000, 1478 Lørenskog, Norway. Phone: (+47) 6796-0000. E-mail: tonje.bjornetro@ahus.no. ORCIDs 0000-0003-2110-5875.

For Review Only

Running title: Monocyte Response to Cancer Extracellular Vesicles

Keywords: Extracellular vesicles, Colorectal cancer, Monocytes, Inflammation, Tumor microenvironment

List of abbreviations:

AP$_{EV}$ EVs from patients with non-invasive adenoma polyps
CRC Colorectal cancer
cryo-EM Cryo-electron microscopy
DAPI 4',6-Diamidino-2-phenylindole
EMD Emerin
EVs Extracellular vesicles
FBS Fetal bovine serum
IL-1β Interleukin-1β
IL-6 Interleukin-6
IL-8 Interleukin-8
IP-10 Interferon gamma-induced protein 10
IPA Ingenuity® Pathway Analysis
local-RC$_{EV}$ EVs from rectal cancer patients with localized disease (no metastasis) at the time of study enrollment
MCP-1 Monocyte chemoattractant protein-1
met-RC$_{EV}$ EVs from rectal cancer patients with metastatic disease at the time of study enrollment
MIP-1β Macrophage inflammatory protein-1β
NTA Nanoparticle tracking analysis
PBS Phosphate-buffered saline
PI Propidium iodide
PRKCD Protein kinase C, delta
RC$_{EV}$ EVs from patients with invasive rectal cancer
SEM Standard error of the mean
SW480$_{EV-S}$ SW480 cell line small EVs
SW480$_{EV-M}$ SW480 cell line medium EVs
TME Tumor microenvironment
TNF-α Tumor necrosis factor-α
TRIM28 Tripartite motif containing 28

For Review Only

Abstract

Extracellular vesicles (EVs) released by tumor cells can directly or indirectly modulate the phenotype and function of the immune cells of the microenvironment locally or at distant sites. The uptake of circulating EVs and the responses by human monocytes *in vitro* may provide new insights into the underlying biology of the invasive and metastatic processes in cancer. Although a mixed population of vesicles is obtained with most isolation techniques, we predominantly isolated exosomes (small EVs) and microvesicles (medium EVs) from the SW480 colorectal cancer cell line (established from a primary adenocarcinoma of the colon) by sequential centrifugation and ultrafiltration, and plasma EVs were prepared from 22 patients with rectal adenoma polyps or invasive adenocarcinoma by size-exclusion chromatography. The EVs were thoroughly characterized. The uptake of SW480 EVs was analyzed, and small SW480 EVs were observed to be more potent than medium SW480 EVs in inducing monocyte secretion of cytokines. The plasma EVs were also internalized by monocytes; however, their cytokine-releasing potency was lower than that of the cell line-derived vesicles. The transcriptional changes in the monocytes highlighted differences between adenoma and adenocarcinoma patient EVs in their ability to regulate biological functions, whereas the most intriguing changes were found in monocytes receiving EVs from patients with metastatic compared to localized cancer.

For Review Only

1.0 Introduction

Tumor cells secrete a large number of extracellular vesicles (EVs) of different intracellular origin, such as exosomes and microvesicles. The EVs carry a range of functionally active components, proteins, nucleic acids and others, dependent on the cell type and state [1]. EVs play an important role in cell-to-cell communication and influence the recipient cells by interacting with cell surface receptors or by transferring their contents of bioactive molecules upon internalization [2]. EVs are involved in the regulation of vascular and epithelial barrier functions in inflamed intestines and in trafficking and activity of resident and recruited immune cells [3].

Cancer cells shape their tumor microenvironment (TME) by communicating with the surrounding stromal cells, including immune-modulating cell types. It is now acknowledged that inflammation is of importance in cancer initiation and progression [4]. The development of invasive colorectal cancer (CRC) in the normal mucosal lining of the bowel is often a stepwise process via adenoma polyps, with genetic and epigenetic changes as early events [5]. Importantly, the TME immune cell types, functional orientation, density, and localization can determine the outcome for CRC patients [6,7]. Tumor-associated macrophages, originating from circulating monocytes, are an abundant component of infiltrating cells in the dynamic TME and important in these processes [8,9]. CRC-derived EVs can act on circulating monocytes and tumor-associated macrophages, affecting the cellular phenotype, polarization, and activity, and thereby modulating responses related to inflammation, tumor growth, and metastasis [10]. Moreover, the local inflammatory mediators play an important role in the induction of a systemic host immune response [11]. Clinically, high blood levels of inflammatory factors are associated with increased risk of death from CRC [12,13].

For Review Only

Rectal cancer typically presents as heterogeneous primary tumor manifestations within the pelvic cavity, and approximately a third of cases will have metastatic disease at presentation [14]. Assessing transcriptional changes and cytokines secreted from *in vitro* monocytes stimulated with CRC cell line- and cancer patient plasma EVs may help understanding their role in tumor development, particularly mechanisms involved in the transition to invasive disease, and the metastatic process. Within this frame of reference, we hypothesized that tumor-derived EVs might be mediators of immune and inflammatory responses that are essential for rectal cancer progression. We isolated and characterized small EVs and medium EVs from a CRC cell line and plasma EVs from patients with rectal adenoma polyps or invasive adenocarcinoma, which were subsequently incubated with human primary monocytes in order to study the uptake and elicited functional responses. All EV specimens were internalized and caused cytokine-releasing activity, whereas the most intriguing changes were found in the transcriptional differences of monocytes receiving EVs from patients with metastatic cancer.

For Review Only

2.0 Materials and methods

2.1 Ethics approval and consent to participate

The prospective biomarker study OxyTarget (NCT01816607) in rectal cancer was approved by the Institutional Review Board and Regional Committee for Medical and Health Research Ethics of South-East Norway (reference number REK 2013/152) and conducted in accordance with the Helsinki Declaration. Written informed consent was required to participate. The monocytes were from healthy blood donors (biobank material access number 908 at Oslo University Hospital), who had provided written consent for blood products to be used for research purposes. The material was used in accordance with the approval under REK 2013/152.

2.2 Health and safety

We confirm that all mandatory laboratory health and safety procedures have been complied within the course of conducting any experimental work reported in this paper.

2.3 CRC cell line and culture conditions

The human colorectal adenocarcinoma SW480 [SW480] (ATCC® CCL228™) cell line, established from a primary adenocarcinoma of the colon from a patient with Dukes´ type B disease (purchased from American Type Culture Collection, Manassas, VA, USA, free of mycoplasma), was grown in RPMI medium (Gibco by Life Technologies, Paisley, UK) supplemented with 10% fetal bovine serum (FBS) (Lonza, Verviers, Belgium) and 1% v/v Penicillin (10,000 U)/Streptomycin (10 mg/ml) (Sigma-Aldrich, Saint Louis, MO, USA). CountessII FL (Thermo Fisher Scientific, Waltham, MA, USA) was used to determine the viability and cell number. The cells were cultured at 37 °C in a 5% CO_2-humidified environment.

For Review Only

The cells were purchased in 2015 and stored at –150 °C, and the current experiments were performed October 2016 - July 2017. Cells from passage 2 were used and kept for a maximum of 6 weeks. Cell authentication (STR DNA profile) was generated by the cell bank using Promega PowerPlex System[2] and GeneMapper Software (Thermo Fisher Scientific).

2.4 Patient and healthy blood donor materials

Citrate plasma was collected at the time of patient enrollment (patient characteristics given in Supplementary Table S1). The samples were prepared by centrifugation at 2,000*g* for 10 minutes, and aliquots were stored at –80 °C. The study cohort presented here consisted of 22 patients; 16 adenocarcinoma cases and 6 cases with benign adenoma polyps.

Primary monocytes were isolated from healthy blood donors by elutriation-purification of EDTA whole blood samples, and cryo-preserved at –150 °C as previously shown [15]. The cells were thawed and resuspended in medium containing 5% exosome-depleted FBS (Thermo Fisher Scientific). Three different donors were used in the conduct of this study: the first for the analyses of SW480-derived EV uptake and internalization (90% purity of CD14$^+$ cells); the second for the analyses of plasma-derived EV uptake and internalization as well as SW480- and plasma-derived EV-induced cytokine- and transcriptional responses by monocytes (88% purity of CD14$^+$ cells); the third for the comparison analyses of SW480-derived EVs in monocyte cytokine secretion (88% purity of CD14$^+$ cells).

2.5 EV isolation

The choice of isolation methods was dependent on the biological material and the downstream analysis to obtain separate fractions of EVs with intact integrity and biological activity [16].

For Review Only

2.5.1 SW480 cell line small EVs (SW480$_{EV\text{-}S}$) and medium EVs (SW480$_{EV\text{-}M}$)

The cells were seeded in T175 flasks (Thermo Fisher Scientific). At 80% confluence, the cells were washed three times with phosphate-buffered saline (PBS; Gibco by Life Technologies), the medium was changed to serum-free medium, and the cells were further incubated for 24 hours. SW480$_{EV\text{-}S}$ and SW480$_{EV\text{-}M}$ were isolated from the conditioned media by sequential centrifugation and ultrafiltration. Briefly, cell debris and floating cells were removed by centrifugation at 300*g* for 10 minutes and 4,500*g* for 5 minutes, and the supernatant was stored at −80 °C. The supernatant was thawed at 37 °C and medium EVs were isolated by centrifugation at 17,000*g* (11,930 rpm, k-factor 2,112) for 30 minutes using a fixed-angle Sorvall SS-34 rotor (Kendro Laboratory Products, Newtown, CT, USA). The pellet was resolved in serum-free medium and concentrated using Amicon Ultra-4 100 kDa Centrifugal Filter Devices (Merck Millipore, Cork, Ireland). Prior to small EV isolation, to exclude particles larger than 220 nm, the 17,000*g*-supernatant was gently filtered through 0.22-μm filter (Merk Millipore). Small EVs were isolated by Centricon-70 Plus 100 kDa Centrifugal Filter Columns (Merk Millipore) and centrifuged at 3,500*g* for 15 minutes with a washing step in PBS at 3,500*g* for 10 minutes. All centrifugations were performed at room temperature. All samples were stored at −80 °C.

For Review Only

2.5.2 Plasma EVs

EVs were isolated from 500 μl citrate plasma using qEV Size Exclusion chromatography Columns (IZON Science, Oxford, UK). The columns were equilibrated with 20 ml of 0.22-μm-filtered PBS/0.32% citrate (Greiner Bio-One GmbH, Kremsmüster, Austria), and EVs were isolated according to the protocol of the vendor. Fractions of 500 μl were collected, and the eluted fraction number 7-10 were concentrated using Amicon Ultra-2 10 kDa Centrifugal Filter Devices (Merck Millipore). All samples were stored at −80 °C. EV specimens from patients

with invasive adenocarcinoma (*i.e.*, rectal cancer) and non-invasive adenoma polyps were termed RC_{EV} and AP_{EV}, respectively.

2.6 EV characterization

2.6.1 Cryo-electron microscopy (cryo-EM) analysis

Holey carbon grids (Cu R2/2; Quantifoil Micro Tools GmbH, Grosslöbichau, Germany) were glow-discharged in a vacuum-filled chamber for 60 seconds using the Balzers SCD 040 sputter coater. The EV samples (3.5 µl) were applied to the grid and incubated for 60 seconds in 100% humidity at 22 °C using the Vitrobot Mark I (Thermo Fisher Scientific). The grids were blotted for 3 seconds using a 55/20 mm filter paper (Ted Pella, Inc., Redding, CA, USA) and frozen in liquid ethane cooled to liquid nitrogen temperature. The vitrified samples were imaged using the JEOL 2100F transmission electron microscope (JEOL Ltd., Tokyo, Japan) operated at 200 keV and a 4K×4K CCD camera (Tietz Video and Imaging Systems GmbH, Germany) at different magnifications (25,000× and 80,000×). The defocus values used for the images ranged from –3.0 µm to –4.5 µm.

2.6.2 Nanoparticle tracking analysis (NTA)

The EV samples were vortexed and diluted in PBS (0.02-µm-filtered; Whatman Anotop™25, GE Healthcare Life Science, Buckinghamshire, UK) to be within the recommended concentration (1.0-9.0×10^8 particles/ml). The samples were loaded into the NS500 instrument (Malvern, Amesbury, UK) by a syringe at a constant flow with a syringe pump speed of 20. Three 60-second videos were captured for each sample (slide shutter 1,200, slider gain 146). The videos were analyzed by NTA 3.1 software (Malvern). The vesicle quantifications had an analytic variance of 2-25% [17].

For Review Only

2.6.3 Immunoblot analysis

Cells and EV specimens were lysed in M-PER® Mammalian Protein Extraction Reagent supplemented with Halt™ Protease Inhibitor Cocktail and Halt™ Phosphatase Inhibitor Cocktail (all from Thermo Fisher Scientific) for 15 minutes on ice and centrifuged at 17,000*g* for 15 minutes at 4 °C. The lysates were prepared in reducing and non-reducing (for analysis of tetraspanins) conditions. Proteins from cell lysates (10 µg), $SW480_{EV-S}$ and $SW480_{EV-M}$ (300 µg), and RC_{EV} (150 µg) were separated by NuPAGE Bis-Tris (Novex by Life Technologies, Carlsbad, CA, USA). The proteins were transferred by electrophoresis to Immobilon-P membranes (Millipore Corporation, Billerica, MA, USA). The primary antibodies were anti-CD9 (Ts9), anti-CD63 (Ts63), and anti-CD81 (1.3.3.22) (all from Thermo Scientific), and anti-GM130 (D6B1) XP (Cell Signaling Technology, La Jolla, CA, USA). Secondary antibodies were from Dako Denmark (Glostrup, Denmark). Peroxidase activity was visualized using SuperSignal West Dura Extended Duration Substrate (Thermo Fisher Scientific) and the ImageQuant Las 3000 system (FujiFilm, Tokyo, Japan). The experiments were performed two or three times.

2.7 EV uptake and internalization by monocytes

2.7.1 PKH67-labeling of EVs

The EVs were labeled using PKH67 Green Fluorescent Cell Linker Kit (Sigma-Aldrich), based on Lässer *et al.* [18]. The dye solution was prepared by adding 1 µl of PKH67 dye per 500 µl of Dye Solution and centrifuged at 17,000*g* for 5 minutes to remove aggregates. The EV samples (per 100 µl) were mixed with 250 µl Diluent C and 250 µl PKH67 Dye Solution and incubated on ice for 5 minutes with frequent vortexing. The reaction was stopped using 500 µl 1% PBS/bovine serum albumin (Sigma-Aldrich). The labeled EVs were concentrated using Amicon Ultra-4 100 kDa Centrifugal Filter Devices, washed three times with PBS followed by

For Review Only

centrifugation at 3,000*g* for 15 minutes, before resuspension in culture medium. All reagents were filtered with a 0.02 µm filter before use. The labeled EVs were stored at 4 °C until use the next day.

2.7.2 EV binding and uptake

Freshly thawed monocytes (1.5×10^5/well) were incubated in a 96-well plate (Costar 3596, Corning, NY, USA) in the absence and presence of PKH67-labeled EVs (1.3×10^9 particles/ml) in RPMI-1640 medium with 5% exosome-depleted FBS for 4 hours at 37 °C in a 5% CO_2-humidified environment. A PKH67-PBS sample was included as negative control. The cells were washed with PBS, detached with 0.25% (w/v) Trypsin (Sigma-Aldrich), and further washed in an Eppendorf tube before evaluation by flow cytometry (BD Accuri C6, BD Biosciences, San Jose, CA, USA). Median fluorescence intensity was used to report uptake, which is composed of internalized and membrane-bound EVs, and 10,000 events were recorded per sample. Viability of monocytes was measured by incubation with anti-CD14 (Beckman Coulter, Marseille, France), Annexin V (BD Bioscience, Norway), and propidium iodide (PI) (Sigma-Aldrich) prior to measurements of median fluorescence intensity.

2.7.3 Cytochalasin D treatment

Monocytes (1.5×10^5/well) were preincubated for 30 minutes at 37 °C in RPMI-1640 medium with 5% exosome-depleted FBS and 10 µg/ml of the phagocytosis inhibitor cytochalasin D (Sigma-Aldrich) prior to a 4-hour incubation with PKH67-labeled $SW480_{EV\text{-}S}$ (1.3×10^9 particles/ml). Optimal inhibitor concentration was determined from dose-response experiments monitoring the inhibition of PKH67-labeled EV uptake combined with viability measurements with quantification of apoptosis using Annexin V/PI and expression of the monocyte marker CD14.

For Review Only

2.7.4 EV internalization

Monocytes (2×10^6/well) were incubated in the presence of PKH67-labeled EVs ($SW480_{EV\text{-}S}$: $6\text{-}7\times10^9$ particles/ml; AP_{EV} and RC_{EV}: $5\text{-}6\times10^{11}$ particles/ml) for 4 hours at 37 °C in a 5% CO_2-humidified environment in 35-mm glass bottom MatTek dishes (P35G-1.5-14-C; MatTek Corporations, Ashland, MA, USA) coated with Poly-D-Lysine (A-003-E, Merck Millipore). The cells were washed with PBS, fixed in 4% paraformaldehyde for 15-20 minutes at room temperature, and mounted with Vectashield mounting media containing 4',6-diamidino-2-phenylindole (DAPI) (H-1200; Vector Laboratories, Burlingame, CA, USA). Analysis of EV internalization was performed using a Leica TCS SP8 confocal microscope equipped with a 100× 1.40 NA oil immersion lens, a UV laser, a continuous wavelength white-light laser set at 490 nm, and transmitted light. Z-stack images were acquired for each sample (0.3 µm between z-planes). Acquired images were further processed using the Fiji ImageJ software (ImageJ, http://imagej.net) [19]. A PKH67-PBS sample was included as negative control.

For Review Only

2.8 EV-induced monocyte phenotypes

2.8.1 Incubation conditions

Monocytes (1.5×10^5/well) were incubated in a 96-well plate in the absence and presence of EVs for 4 hours at 37 °C in a 5% CO_2-humidified environment. Cell viability was determined by CellTiter 96®AQ_{ueous} One Solution Reagent (Promega, Madison, WI, USA) according to the manufacturer´s protocol. EVs were given in a dose-dependent manner (0.1×10^{10}, 0.5×10^{10}, and 1.0×10^{10} particles/ml) in an independent experiment or with a set concentration (0.3×10^{10} particles/ml) to monocytes from a second donor to assess the differences in response to $SW480_{EV\text{-}S}$ and $SW480_{EV\text{-}M}$. Plasma EVs were given to the monocytes as a set volume (50 ul of the concentrated EV-sample) corresponding to $1.5\times10^{10}\text{-}1.9\times10^{11}$ particles/ml, thus reflecting each individual patient’s total EV amount in the blood. The conditioned media were

vortexed and centrifuged at 10,000*g* for 10 minutes at 4 °C before the supernatants were stored at –80 °C for multiplex cytokine analysis (Luminex). The monocytes were directly lysed in QIAzol lysis buffer (Qiagen, Hilden, Germany) prior to RNA isolation.

2.8.2 Multiplex immunoassay analysis

The supernatants were thawed, and 50 µl of the samples were analyzed in duplicates on the same plate using a custom-made human cytokine 7-plex assay (Bio-Rad Laboratories, Hercules, CA, USA) for the simultaneous measurement of interleukin (IL)-1β, IL-6, IL-8, tumor necrosis factor-α (TNF-α), interferon gamma-induced protein 10 (IP-10), macrophage inflammatory protein-1β (MIP-1β), and monocyte chemoattractant protein-1 (MCP-1). The plates were washed with the magnetic plate washer Bio-Plex Pro Wash Station (Bio-Rad Laboratories). The analyses were performed using Luminex IS 100 (Luminex-Corp., Austin, TX, USA) with the Bio-Plex software version 6.0.1 (Bio-Rad Laboratories). Intra-percent coefficient of variation was within acceptable range for values <8.

For Review Only

2.8.3 Gene expression analysis

Total RNA was isolated using miRNeasy Micro Kit (Qiagen), following the manufacturers protocol, with additional use of phase-lock tubes (5 PRIME GmbH, Hamburg, Germany). RNA concentration (4-15 ng/µl) and quality (RIN >8) were assessed using Nano Drop spectrophotometer (Saveen Werner, Limhamn, Sweden) and Agilent BioAnalyzer 2100 (Agilent Technologies, Santa Clara, CA, USA). Total RNA (10 ng) was subjected to the GeneChip™ WT Pico Reagent Kit (Thermo Fisher Scientific). Microarray analysis was performed using the Affymetrix Human Clariom™ S Arrays (Affymetrix, Santa Clara, CA, USA), containing more than 20,000 well-annotated genes. Signal intensities were detected by the Hewlett Packard (Palo Alto, CA, USA) 30007G gene array scanner and processed using the

Affymetrix GeneChip Command Console software. The extracted data were imported into the Partek® Genomics Suite ™ software (Partek Inc., Saint Louis) and the Robust Multichip Analysis algorithm was applied for generation of relative signal values and normalization.

For technical validation, selected differentially expressed genes were analyzed with qRT-PCR (TaqMan gene expression assays and the Applied Biosystems ViiA7 sequence detection system) in all patients (n=22). Total RNA (20 ng) was reverse transcribed using SuperScript IV VILO cDNA Synthesis kit (Thermo Fisher Scientific). cDNA was diluted 1:10 and analyzed in 20 µl reactions (3 technical replicates) using TaqMan® Fast Advanced Master Mix (Applied Biosystems) and the following primers: Emerin (*EMD*) Hs02560738_s1, Protein kinase C, delta (*PRKCD*) Hs01090047_m1, and Tripartite motif containing 28 (*TRIM28*) Hs00232212_m1 as target genes. The relative changes of each transcript were analyzed using the mean of Alanyl aminopeptidase (*ANPEP*) Hs00174265_m1, Ribosomal protein L13A (*RLP13A*) Hs04194366_g1, and Ribosomal protein L30 (*PRP30*) Hs00265497 as endogenous controls and ViiA7 Software v1.2. Pearson correlation was computed using Graphpad Prism 8.3.0.

2.8.4 Statistical analysis and functional annotation of array data

Following quality control and pre-processing, the data were $\log_2$-transformed. Differential gene expression between the groups [monocytes incubated with RC_{EV} or AP_{EV}, and monocytes incubated with EVs from patients with metastasis (met-RC_{EV}) or localized disease (no metastasis; local-RC_{EV}) at the time of study enrollment] was determined using a one-way ANOVA model with p-value cut-off of 0.05 and fold-change cut-off of 1.2. Signal values were subjected to clustering using the Partek software. Ingenuity® Pathway Analysis (IPA; QIAGEN inc., https://www.qiagenbioinformatics.com) core analysis was used to identify involved functions and GO annotations. In IPA, Fisher´s Exact Test was used to calculate p-

For Review Only

values determining the probability that the given functions did not represent findings due to chance, and correction for multiple testing was accounted for, when possible, by the Benjamin-Hochberg method. Z-scores was calculated by the IPA-z-score algorithm to generate predictions about the direction of change in functions, and an absolute z-score ≥ 2 was considered significant.

2.9 Other statistical analyses

The data are presented as mean ± standard deviation (SD), mean ± standard error (SEM), or median with range. In cell line experiments, the differences between groups were determined with two-tailed Student's *t*-test. The statistical analyses were performed using IBM SPSS Statistics for Mac v25 and Graphpad Prism 8.3.0, and *p*-values <0.05 were considered significant.

For Review Only

3.0 Results

3.1 EV characteristics

Features of isolated SW480$_{EV-S}$, SW480$_{EV-M}$, AP$_{EV}$, and RC$_{EV}$ were characterized by several methods. Cryo-EM revealed a visible membrane bilayer for SW480$_{EV-S}$, SW480$_{EV-M}$, and RC$_{EV}$; while the two former had a diameter size in the range of 30-90 nm and 60-200 nm (n=1), respectively, the patient samples showed a heterogeneous population of mostly 30-100 nm-sized EVs but some >100 nm (n=1) (Figure 1a). Immunoblot analysis confirmed expression of the EV-enriched proteins CD63, CD9, and CD81 and the absence of the Golgi apparatus marker GM130 (often used as control for cellular contamination of EV samples) by SW480$_{EV-S}$, SW480$_{EV-M}$, and RC$_{EV}$ (Figure 1b). Further measurements by NTA showed a mode size of 90.9±8.30 nm (mean±SEM; n=3) for SW480$_{EV-S}$, 117±17.9 nm (mean±SEM; n=3) for SW480$_{EV-M}$, 91.30±4.56 nm (mean±SEM; n=6) for AP$_{EV}$, and 92.6±3.90 nm (mean±SEM; n=16) for RC$_{EV}$. No significant difference in plasma EV concentration (particles per ml) was found between AP$_{EV}$ ($2.30\times10^{11}\pm3.96\times10^{10}$; mean±SEM) and RC$_{EV}$ ($2.09\times10^{11}\pm3.44\times10^{10}$; mean±SEM), with a median of 2.12×10^{11} (range, 5.52×10^{10}- 5.50×10^{11}) particles per ml for all patients (Figure 1c).

For Review Only

3.2 EV uptake and internalization by monocytes

Flow cytometry analysis showed enhancement of approximately 10- to 20-fold in median fluorescent intensity in monocytes after 4 hours of incubation with PKH67-labeled SW480-derived small and medium EVs (n=3), and plasma EVs (n=1) (Supplementary Figure S1a, b and c). The internalization of SW480$_{EV-S}$ was further studied by confocal microscopy after incubation with PKH67-labeled vesicles, which were clearly detected in the monocyte cytoplasm (Figure 2 and Supplementary Figure S2). Confocal microscopy analysis of PKH67-

labeled plasma EVs also showed internalization and cytoplasmic distribution (Figure 3 and Supplementary Figure S3). Each experiment was performed once.

Because plasma EV preparations contain a complex mixture of various EV types secreted from many types of blood cells and tissues, SW480$_{\text{EV-S}}$ were chosen for analyzing the contribution of phagocytosis to the endocytic pathways used by the primary monocytes for EV uptake. The toxicity of the phagocytosis inhibitor cytochalasin D (10 μg/ml) on monocytes was determined as negligible (data not shown). The amount of PKH67-labeled SW480$_{\text{EV-S}}$ associated with monocytes was measured by flow cytometry in the absence and presence of cytochalasin D and shown to be reduced by an average of 36% by cytochalasin D (n=3) (Supplementary Figure S1d).

For Review Only

3.3 EV-induced monocyte phenotypes

Since all the investigated EV populations were able to enter the monocytes, the question arose whether immune or inflammatory responses might be elicited. First, following the 4-hour EV incubation, monocyte supernatants were analyzed for seven cytokines selected on the basis of a previous screening of monocyte responses to SW480$_{\text{EV-S}}$ (unpublished data). A strong and dose-dependent secretion was observed for IL-1β, IL-6, IL-8, MIP-1β, TNF-α, and MCP-1 in particular, whereas IP-10 showed a moderate increase (Supplementary Figure S4). In general, SW480$_{\text{EV-S}}$ were more potent than SW480$_{\text{EV-M}}$ (Figure 4a). The monocyte viability was not affected by EVs (data not shown). The monocytes incubated with AP$_{\text{EV}}$, local-RC$_{\text{EV}}$, or met-RC$_{\text{EV}}$ showed a more moderate increase in the secretion responses, several even below the controls (Figure 4b). The MCP-1 measures were not reliable because of technical challenges.

Interestingly, incubation with AP_{EV}, local-RC_{EV}, and met-RC_{EV} evoked clearly distinguishable transcriptional responses by monocytes (Figure 5a). Using a fold-change cut-off of 1.2, RC_{EV} caused differential expression of 85 genes (48 up-regulated, 37 down-regulated) when compared to AP_{EV} (Figure 5b). When comparing met-RC_{EV} and local-RC_{EV}, the vesicles from patients with metastatic disease led to 618 differentially expressed genes (410 up-regulated, 208 down-regulated) (Figure 5b). Four transcripts were found to be jointly regulated in monocytes in the comparisons of RC_{EV} *versus* AP_{EV} and met-RC_{EV} *versus* local-RC_{EV} (Figure 5b), and involved in protein binding, apoptotic mitochondrial changes, immune cell signaling, and cell growth, among other biological processes (Supplementary Table S2). The top 10 up- and down-regulated transcripts in monocytes incubated with EVs were involved in a range of metabolic and immune system processes (Supplementary Table S3).

For Review Only

Among the five most discriminating RNA profile-defined functions, when applying the IPA software, were cell morphology, cellular assembly and organization, and organ morphology for AP_{EV} *versus* RC_{EV}, and endocrine system disorders, gastrointestinal disease, and hematological disease for met-RC_{EV} *versus* local-RC_{EV} (Table 1; the transcript IDs assigned to the disease and disorder categories are provided in Supplementary Table S4). Associations with the categories cancer and organismal injury and abnormalities were found for both comparisons (Table 1 and Supplementary Table S4). All patients with disseminated disease had liver metastasis (one had additional lung and bone metastases). For the met-RC_{EV} *versus* local-RC_{EV} comparison, a higher complexity and significance were found for transcripts involved in the categories cancer, organismal injury and abnormalities, and gastrointestinal disease (Figure 5c; the transcript IDs assigned to the disease and function categories are provided in Supplementary Table S4 and S5). However, the categories cell death and survival, immune cell trafficking, and inflammatory response were involved to a similar significance level in the various patient groups (Figure 5c

and Supplementary Table S5). Predictions on the direction of change for the functions were only applicable for the dataset of met-RC_{EV} *versus* local-RC_{EV}. A negative or positive z-score value indicates that the function was predicted to decrease or increase in monocytes receiving EVs from patients with metastatic disease compared to patients with localized disease. Organismal death (z-score of –3.020, p=0.014) was among the functions that were predicted to significantly decrease, whereas polarity of cells (z-score of 2.433, p=0.014), catabolism of protein (z-score of 2.149, p=0.015), and infections of cells (z-score of 2.085, p=0.0078) were predicted to significantly increase (Figure 5d).

Technical validation of the gene expression array by qRT-PCR resulted in the following correlations: *EMD* (Pearson r=0.583, p=0.0044), *PRKCD* (Pearson r=0.555, p=0.0073), and *TRIM28* (Pearson r=0.405, p=0.061).

For Review Only

4.0 Discussion

Presuming tumor-derived EVs are critical in shaping an inflammatory TME and thereby facilitate disease dissemination to distant organs, we assessed the uptake and functional responses by human monocyte cultures of CRC cell line small EVs ($SW480_{EV\text{-}S}$) and medium EVs ($SW480_{EV\text{-}M}$) as well as plasma EVs from patients with rectal adenoma polyps (AP_{EV}) and localized (local-RC_{EV}) or metastatic (met-RC_{EV}) adenocarcinoma. All vesicle types were efficiently internalized by monocytes. $SW480_{EV\text{-}S}$ were more potent in inducing cytokine responses than $SW480_{EV\text{-}M}$ on direct comparison. The AP_{EV}, local-RC_{EV}, and met-RC_{EV} evoked clearly distinguishable transcriptional responses with the most compelling evidence of biologically relevant EV-directed pathophysiology for patients with metastatic cancer.

For Review Only

Circulating EV levels have been reported as elevated in CRC patients compared to healthy subjects [20,21]. There were no differences in the quantity of plasma EVs in our small cohort of patients with rectal cancer or adenoma polyps. The cultured monocytes internalized all of the investigated EV types. The uptake of $SW480_{EV\text{-}S}$ in monocytes was partly inhibited by cytochalasin D, indicating that phagocytosis was involved but was not the only uptake mechanism [22,23]. This is in accordance with previous reports showing that the uptake of EVs by monocytes and macrophages is partly dependent on dynamin-dependent endocytic pathways and phagocytosis [24,25]. Specificity of labeling is a limitation for EV visualization using PKH67 [26]. This lipophilic dye was not used when performing functional assays with EVs.

The functional effect of exposure of CRC-derived EVs on monocyte/macrophage differentiation and polarization is complex and probably dependent on the cell origin of the EVs, the timing of exposure, as well as the stage of maturation of the recipient cell [10]. Early studies showed that *in vitro* stimulation of human monocytes with CRC-EVs altered the

monocyte phenotype towards antitumor activity [27,28]. Moreover, monocytes were differentiated by CRC-EVs into macrophages with a mixed polarization status (M1/M2) depending on contact time and vesicle cargo [24,28]. Others have described an M2 phenotype after exposure to CRC-EVs, promoting *in vivo* tumor growth and development of metastasis [29,30]. Our study additionally addressed the differential cytokine release by cell line small EVs and medium EVs, and the impact of EVs isolated from the circulation in patients with rectal benign or malignant tumors. Both $SW480_{EV\text{-}S}$ and $SW480_{EV\text{-}M}$ increased the cytokine secretion by the monocytes and based on equal number of vesicles given, small EVs seemed to be more potent. This indicates that the nature of the vesicles is important, but we could not rule out that there is a balance or threshold that needs to be achieved to detect an effect or that freezing and thawing could have affected EV integrity and function of the vesicles. There is also a possibility that the EV-associated functions are partly mediated by non-EV-bound components co-isolated with the different fractions, or by a combination of both [31]. Our experiments were compared to a medium (EV-free) control condition. A detailed investigation into the effects of EV fractions, EV-depleted fractions, and unfractionated starting material (initial fluid) might have determined the relative contribution of each [32]. Another approach is to compare the activity of intact EVs with EV samples treated with detergents to destroy the vesicle structure [33]. Altogether, our data endorse the role of EV-enriched preparations as profound modulators of monocyte's biological activity.

Increased monocyte or macrophage release of TNF-α, IL-1β, and IP-10 has previously been reported upon exposure of CRC cell line-derived EVs [24,27,28,34]. The cytokine levels in the circulation and tumors in CRC patients have been associated with disease progression and poor survival [13,35-37]. SW480 is a well known and commonly used cell line for investigating CRC-derived EVs effects on monocytes/macrophages [24,28] and in this study it was used to

establish a reliable model system for monocyte responses of cancer-derived EVs. In our experimental setting, AP_{EV} and RC_{EV} were less potent than EVs from SW480 cells with regard to the cytokine responses. Unexpectedly, few of the patient specimens induced secretion above the controls. We can not exclude the possibility that EVs from a colon cancer cell line are dissimilar to circulating EVs from rectal cancer patients, or that the patient-derived EVs might stimulate the secretion of other cytokines (*e.g.*, anti-inflammatory cytokines) than the ones recorded in this study. It is important to note that blood harbors a great number of EVs derived from blood cells (platelets, erythrocytes, lymphocytes, granulocytes, and monocytes) in addition to possible tumor EVs [38], and the dose of cancer-derived EVs in our study might be too low to elicit a measurable effect.

For Review Only

In the global analysis of transcriptional monocyte responses to EVs, the functional categories cancer, organismal injury and abnormalities, and gastrointestinal disease were more strongly induced in monocytes given plasma EVs from patients with metastatic disease than from cases with tumor confined to the pelvic cavity. In the comparison of downstream effect analysis, these categories were also shown with stronger significance in the group of patients with metastasis. Interestingly, we observed that the plasma EVs from patients with invasive cancer compared to adenoma polyps caused effects that are essential in cancer. This category had annotations such as carcinoma, vascularization of tumor, and function of mitochondria. Further along this line, additional annotations associated to plasma EVs from patients with metastatic disease were tumorigenesis of tissue, gastrointestinal carcinoma, and hematologic cancer. Many of the identified genes linked to the category gastrointestinal disease have been shown to be involved in CRC initiation and development as well as intestinal inflammation [39-41]. The categories inflammatory response, immune cell trafficking, and cell death and survival were involved to a similar significance level in the two patient comparisons. However, EVs from patients with

metastatic disease were predicted to repress functions involved in organismal death by up-regulation of transcripts essential in cellular development and survival, and in tumor progression *in vitro*. Some upregulated transcripts were associated with death in patients with several cancer types. IPA also predicted activation of polarity of monocytes, with transcripts regulating motility and adhesion, cell metabolism, and functions involved in infection of cells, such as viruses resembling vesicles in physical and chemical characteristics. These findings may reflect the interaction between the cancer and the detrimental immune response in patients with disseminated CRC that commonly is seen in clinical practice [42].

Our findings are in strong support of *in vitro* monocytes being a relevant model system for investigating local TME and systemic host responses to circulating EVs in rectal cancer patients. Limitations that need to be taken into consideration are the small patient cohort with non-recorded co-morbidities that might have affected the results. Each group comprised a heterogeneous patient population, and the circulating EVs were derived from individuals with the only joint and controllable feature of a rectal tumor. We also acknowledge that there could be individual differences in the effects of tumor-derived EVs on monocytes isolated from different healthy donors. However, because of limitation of biological material (plasma volume for EV isolation) from the rectal cancer patients, it was required to focus the main experiment on monocytes from only one donor. The monocyte transcriptional differences among the groups were rather low with the fold-change cut-off of 1.2; more stringent settings would have made the data ineligible for enrichment analysis by the IPA software.

In conclusion, plasma EVs from patients diagnosed with a rectal tumor were internalized by human primary monocytes cultured *in vitro* and caused responses related to the disease progression from non-invasive to invasive and disseminated cancer. At the gene expression

For Review Only

level, the monocyte responses distinguished EVs from subjects with adenoma polyps from cases with invasive cancer that was localized within the pelvic cavity and further from those with metastatic disease. Additional studies of circulating patient EVs are still needed to understand their evident role in cancer immunity and inflammation.

For Review Only

5.0 Acknowledgements

The authors thank Hanna Abrahamsson and Sebastian Meltzer at Department of Oncology at Akershus University Hospital for the selection of study patients with adenoma polyps and rectal cancer. We also appreciate the technical assistance from the Flow Cytometry Core Facility at Oslo University Hospital – Ullevål, the Affymetrix Core Facility at Oslo University Hospital – Ullevål, and the Core Facility of Advanced Light Microscopy at Oslo University Hospital – Radiumhospitalet and Gaustad. Graphical abstract was created with BioRender.com.

6.0 Funding sources

This work was supported by the South-Eastern Norway Regional Health Authority under Grants 2014010, 2016050, 2017109, 2018042 and 2019109; Akershus University Hospital under Grants 2017013 and 2018004; and the Karolinska Institute contribution under the Science Council Grant 2016-03810.

For Review Only

7.0 Disclosure of conflict of interest

The authors report no conflicts of interest

8.0 Author contributions

TB, KRR, RØ, and AHR were responsible for the concept and experimental design. TB, LAS, BV, BSB, AMT, KBFH, ALl, SOB and RØ were involved in the development of methodology. TB, LAS, BSB, OKO, HCDA, ALo and RS performed the experiments, analyzed and interpreted the data. TB, RØ, AHR wrote the manuscript. All authors read and approved the manuscript.

9.0 Data availability

Request to inspect and analyze the data that underlie the results in this article, including the RNA array data, should be directed to the corresponding author, and access will be provided in accordance with the General Data Protection Regulation of the European Union.

For Review Only

References

1. Robbins PD, Morelli AE (2014) Regulation of immune responses by extracellular vesicles. Nat Rev Immunol;14(3):195-208 doi: 10.1038/nri3622
2. Yanez-Mo M, Siljander PR, Andreu Z, Zavec AB, Borras FE, Buzas EI, Buzas K, Casal E, Cappello F, Carvalho J, Colas E, Cordeiro-da Silva A, Fais S, Falcon-Perez JM, Ghobrial IM, Giebel B, Gimona M, Graner M, Gursel I, Gursel M, Heegaard NH, Hendrix A, Kierulf P, Kokubun K, Kosanovic M, Kralj-Iglic V, Kramer-Albers EM, Laitinen S, Lasser C, Lener T, Ligeti E, Line A, Lipps G, Llorente A, Lotvall J, Mancek-Keber M, Marcilla A, Mittelbrunn M, Nazarenko I, Nolte-'t Hoen EN, Nyman TA, O'Driscoll L, Olivan M, Oliveira C, Pallinger E, Del Portillo HA, Reventos J, Rigau M, Rohde E, Sammar M, Sanchez-Madrid F, Santarem N, Schallmoser K, Ostenfeld MS, Stoorvogel W, Stukelj R, Van der Grein SG, Vasconcelos MH, Wauben MH, De Wever O (2015) Biological properties of extracellular vesicles and their physiological functions. J Extracell Vesicles;4:27066 doi: 10.3402/jev.v4.27066
3. Bui TM, Mascarenhas LA, Sumagin R (2018) Extracellular vesicles regulate immune responses and cellular function in intestinal inflammation and repair. Tissue Barriers;6(2):e1431038 doi: 10.1080/21688370.2018.1431038
4. Terzic J, Grivennikov S, Karin E, Karin M (2010) Inflammation and colon cancer. Gastroenterology;138(6):2101-14 doi: 10.1053/j.gastro.2010.01.058
5. Brenner H, Kloor M, Pox CP (2014) Colorectal cancer. Lancet;383(9927):1490-502 doi: 10.1016/S0140-6736(13)61649-9
6. Pages F, Mlecnik B, Marliot F, Bindea G, Ou FS, Bifulco C, Lugli A, Zlobec I, Rau TT, Berger MD, Nagtegaal ID, Vink-Borger E, Hartmann A, Geppert C, Kolwelter J, Merkel S, Grutzmann R, Van den Eynde M, Jouret-Mourin A, Kartheuser A, Leonard D, Remue C, Wang JY, Bavi P, Roehrl MHA, Ohashi PS, Nguyen LT, Han S, MacGregor HL, Hafezi-Bakhtiari S, Wouters BG, Masucci GV, Andersson EK, Zavadova E, Vocka M, Spacek J, Petruzelka L, Konopasek B, Dundr P, Skalova H, Nemejcova K, Botti G, Tatangelo F, Delrio P, Ciliberto G, Maio M, Laghi L, Grizzi F, Fredriksen T, Buttard B, Angelova M, Vasaturo A, Maby P, Church SE, Angell HK, Lafontaine L, Bruni D, El Sissy C, Haicheur N, Kirilovsky A, Berger A, Lagorce C, Meyers JP, Paustian C, Feng Z, Ballesteros-Merino C, Dijkstra J, van de Water C, van Lent-van Vliet S, Knijn N, Musina AM, Scripcariu DV, Popivanova B, Xu M, Fujita T, Hazama S, Suzuki N, Nagano H, Okuno K, Torigoe T, Sato N, Furuhata T, Takemasa I, Itoh K, Patel PS, Vora HH, Shah B, Patel JB, Rajvik KN, Pandya SJ, Shukla SN, Wang Y, Zhang G, Kawakami Y, Marincola FM, Ascierto PA, Sargent DJ, Fox BA, Galon J (2018) International validation of the consensus Immunoscore for the classification of colon cancer: a prognostic and accuracy study. Lancet;391(10135):2128-39 doi: 10.1016/S0140-6736(18)30789-X
7. Anitei MG, Zeitoun G, Mlecnik B, Marliot F, Haicheur N, Todosi AM, Kirilovsky A, Lagorce C, Bindea G, Ferariu D, Danciu M, Bruneval P, Scripcariu V, Chevallier JM, Zinzindohoue F, Berger A, Galon J, Pages F (2014) Prognostic and predictive values of the immunoscore in patients with rectal cancer. Clin Cancer Res;20(7):1891-9 doi: 10.1158/1078-0432.CCR-13-2830
8. Shi Y, Li Z, Zheng W, Liu X, Sun C, Laugsand JB, Liu Z, Cui G (2015) Changes of immunocytic phenotypes and functions from human colorectal adenomatous stage to cancerous stage: Update. Immunobiology;220(10):1186-96 doi: 10.1016/j.imbio.2015.06.003

For Peer Review Only

9. Richards DM, Hettinger J, Feuerer M (2013) Monocytes and macrophages in cancer: development and functions. Cancer Microenviron;6(2):179-91 doi: 10.1007/s12307-012-0123-x
10. Manning S, Danielson KM (2018) The immunomodulatory role of tumor-derived extracellular vesicles in colorectal cancer. Immunol Cell Biol;96:733-41 doi: 10.1111/imcb.12038
11. Lin WW, Karin M (2007) A cytokine-mediated link between innate immunity, inflammation, and cancer. J Clin Invest;117(5):1175-83 doi: 10.1172/JCI31537
12. McMillan DC (2013) The systemic inflammation-based Glasgow Prognostic Score: a decade of experience in patients with cancer. Cancer Treat Rev;39(5):534-40 doi: 10.1016/j.ctrv.2012.08.003
13. Olsen RS, Nijm J, Andersson RE, Dimberg J, Wagsater D (2017) Circulating inflammatory factors associated with worse long-term prognosis in colorectal cancer. World J Gastroenterol;23(34):6212-9 doi: 10.3748/wjg.v23.i34.6212
14. Riihimaki M, Hemminki A, Sundquist J, Hemminki K (2016) Patterns of metastasis in colon and rectal cancer. Sci Rep;6:29765 doi: 10.1038/srep29765
15. Lund PK, Joo GB, Westvik AB, Ovstebo R, Kierulf P (2000) Isolation of monocytes from whole blood by density gradient centrifugation and counter-current elutriation followed by cryopreservation: six years' experience. Scand J Clin Lab Invest;60(5):357-65 doi: 10.1080/003655100750019260
16. Konoshenko MY, Lekchnov EA, Vlassov AV, Laktionov PP (2018) Isolation of Extracellular Vesicles: General Methodologies and Latest Trends. Biomed Res Int;2018:8545347 doi: 10.1155/2018/8545347
17. Vestad B, Llorente A, Neurauter A, Phuyal S, Kierulf B, Kierulf P, Skotland T, Sandvig K, Haug KBF, Ovstebo R (2017) Size and concentration analyses of extracellular vesicles by nanoparticle tracking analysis: a variation study. J Extracell Vesicles;6(1):1344087 doi: 10.1080/20013078.2017.1344087
18. Lasser C, Alikhani VS, Ekstrom K, Eldh M, Paredes PT, Bossios A, Sjostrand M, Gabrielsson S, Lotvall J, Valadi H (2011) Human saliva, plasma and breast milk exosomes contain RNA: uptake by macrophages. J Transl Med;9:9 doi: 10.1186/1479-5876-9-9
19. Schindelin J, Arganda-Carreras I, Frise E, Kaynig V, Longair M, Pietzsch T, Preibisch S, Rueden C, Saalfeld S, Schmid B, Tinevez JY, White DJ, Hartenstein V, Eliceiri K, Tomancak P, Cardona A (2012) Fiji: an open-source platform for biological-image analysis. Nat Methods;9(7):676-82 doi: 10.1038/nmeth.2019
20. Silva J, Garcia V, Rodriguez M, Compte M, Cisneros E, Veguillas P, Garcia JM, Dominguez G, Campos-Martin Y, Cuevas J, Pena C, Herrera M, Diaz R, Mohammed N, Bonilla F (2012) Analysis of exosome release and its prognostic value in human colorectal cancer. Genes Chromosomes Cancer;51(4):409-18 doi: 10.1002/gcc.21926
21. Yoshioka Y, Kosaka N, Konishi Y, Ohta H, Okamoto H, Sonoda H, Nonaka R, Yamamoto H, Ishii H, Mori M, Furuta K, Nakajima T, Hayashi H, Sugisaki H, Higashimoto H, Kato T, Takeshita F, Ochiya T (2014) Ultra-sensitive liquid biopsy of circulating extracellular vesicles using ExoScreen. Nat Commun;5:3591 doi: 10.1038/ncomms4591
22. Mulcahy LA, Pink RC, Carter DR (2014) Routes and mechanisms of extracellular vesicle uptake. J Extracell Vesicles;3:24641 doi: 10.3402/jev.v3.24641

For Review Only

23. van Niel G, D'Angelo G, Raposo G (2018) Shedding light on the cell biology of extracellular vesicles. Nat Rev Mol Cell Biol;19(4):213-28 doi: 10.1038/nrm.2017.125

24. Popena I, Abols A, Saulite L, Pleiko K, Zandberga E, Jekabsons K, Endzelins E, Llorente A, Line A, Riekstina U (2018) Effect of colorectal cancer-derived extracellular vesicles on the immunophenotype and cytokine secretion profile of monocytes and macrophages. Cell Commun Signal;16(1):17 doi: 10.1186/s12964-018-0229-y

25. Feng D, Zhao WL, Ye YY, Bai XC, Liu RQ, Chang LF, Zhou Q, Sui SF (2010) Cellular internalization of exosomes occurs through phagocytosis. Traffic;11(5):675-87 doi: 10.1111/j.1600-0854.2010.01041.x

26. Takov K, Yellon DM, Davidson SM (2017) Confounding factors in vesicle uptake studies using fluorescent lipophilic membrane dyes. J Extracell Vesicles;6(1):1388731 doi: 10.1080/20013078.2017.1388731

27. Baj-Krzyworzeka M, Szatanek R, Weglarczyk K, Baran J, Zembala M (2007) Tumour-derived microvesicles modulate biological activity of human monocytes. Immunol Lett;113(2):76-82 doi: 10.1016/j.imlet.2007.07.014

28. Baj-Krzyworzeka M, Mytar B, Szatanek R, Surmiak M, Weglarczyk K, Baran J, Siedlar M (2016) Colorectal cancer-derived microvesicles modulate differentiation of human monocytes to macrophages. J Transl Med;14:36 doi: 10.1186/s12967-016-0789-9

29. Shinohara H, Kuranaga Y, Kumazaki M, Sugito N, Yoshikawa Y, Takai T, Taniguchi K, Ito Y, Akao Y (2017) Regulated Polarization of Tumor-Associated Macrophages by miR-145 via Colorectal Cancer-Derived Extracellular Vesicles. J Immunol;199(4):1505-15 doi: 10.4049/jimmunol.1700167

30. Takano Y, Masuda T, Iinuma H, Yamaguchi R, Sato K, Tobo T, Hirata H, Kuroda Y, Nambara S, Hayashi N, Iguchi T, Ito S, Eguchi H, Ochiya T, Yanaga K, Miyano S, Mimori K (2017) Circulating exosomal microRNA-203 is associated with metastasis possibly via inducing tumor-associated macrophages in colorectal cancer. Oncotarget;8(45):78598-613 doi: 10.18632/oncotarget.20009

31. Théry C, Witwer KW, Aikawa E, Alcaraz MJ, Anderson JD, Andriantsitohaina R, Antoniou A, Arab T, Archer F, Atkin-Smith GK, Ayre DC, Bach J-M, Bachurski D, Baharvand H, Balaj L, Baldacchino S, Bauer NN, Baxter AA, Bebawy M, Beckham C, Bedina Zavec A, Benmoussa A, Berardi AC, Bergese P, Bielska E, Blenkiron C, Bobis-Wozowicz S, Boilard E, Boireau W, Bongiovanni A, Borràs FE, Bosch S, Boulanger CM, Breakefield X, Breglio AM, Brennan MÁ, Brigstock DR, Brisson A, Broekman MLD, Bromberg JF, Bryl-Górecka P, Buch S, Buck AH, Burger D, Busatto S, Buschmann D, Bussolati B, Buzás EI, Byrd JB, Camussi G, Carter DRF, Caruso S, Chamley LW, Chang Y-T, Chen C, Chen S, Cheng L, Chin AR, Clayton A, Clerici SP, Cocks A, Cocucci E, Coffey RJ, Cordeiro-da-Silva A, Couch Y, Coumans FAW, Coyle B, Crescitelli R, Criado MF, D'Souza-Schorey C, Das S, Datta Chaudhuri A, de Candia P, De Santana EF, De Wever O, del Portillo HA, Demaret T, Deville S, Devitt A, Dhondt B, Di Vizio D, Dieterich LC, Dolo V, Dominguez Rubio AP, Dominici M, Dourado MR, Driedonks TAP, Duarte FV, Duncan HM, Eichenberger RM, Ekström K, El Andaloussi S, Elie-Caille C, Erdbrügger U, Falcón-Pérez JM, Fatima F, Fish JE, Flores-Bellver M, Försönits A, Frelet-Barrand A, Fricke F, Fuhrmann G, Gabrielsson S, Gámez-Valero A, Gardiner C, Gärtner K, Gaudin R, Gho YS, Giebel B, Gilbert C, Gimona M, Giusti I, Goberdhan DCI, Görgens A, Gorski SM, Greening DW, Gross JC, Gualerzi A, Gupta GN, Gustafson D, Handberg A, Haraszti RA, Harrison P, Hegyesi

For Review Only

H, Hendrix A, Hill AF, Hochberg FH, Hoffmann KF, Holder B, Holthofer H, Hosseinkhani B, Hu G, Huang Y, Huber V, Hunt S, Ibrahim AG-E, Ikezu T, Inal JM, Isin M, Ivanova A, Jackson HK, Jacobsen S, Jay SM, Jayachandran M, Jenster G, Jiang L, Johnson SM, Jones JC, Jong A, Jovanovic-Talisman T, Jung S, Kalluri R, Kano S-i, Kaur S, Kawamura Y, Keller ET, Khamari D, Khomyakova E, Khvorova A, Kierulf P, Kim KP, Kislinger T, Klingeborn M, Klinke DJ, Kornek M, Kosanović MM, Kovács ÁF, Krämer-Albers E-M, Krasemann S, Krause M, Kurochkin IV, Kusuma GD, Kuypers S, Laitinen S, Langevin SM, Languino LR, Lannigan J, Lässer C, Laurent LC, Lavieu G, Lázaro-Ibáñez E, Le Lay S, Lee M-S, Lee YXF, Lemos DS, Lenassi M, Leszczynska A, Li ITS, Liao K, Libregts SF, Ligeti E, Lim R, Lim SK, Linē A, Linnemannstöns K, Llorente A, Lombard CA, Lorenowicz MJ, Lörincz ÁM, Lötvall J, Lovett J, Lowry MC, Loyer X, Lu Q, Lukomska B, Lunavat TR, Maas SLN, Malhi H, Marcilla A, Mariani J, Mariscal J, Martens-Uzunova ES, Martin-Jaular L, Martinez MC, Martins VR, Mathieu M, Mathivanan S, Maugeri M, McGinnis LK, McVey MJ, Meckes DG, Meehan KL, Mertens I, Minciacchi VR, Möller A, Møller Jørgensen M, Morales-Kastresana A, Morhayim J, Mullier F, Muraca M, Musante L, Mussack V, Muth DC, Myburgh KH, Najrana T, Nawaz M, Nazarenko I, Nejsum P, Neri C, Neri T, Nieuwland R, Nimrichter L, Nolan JP, Nolte-'t Hoen ENM, Noren Hooten N, O'Driscoll L, O'Grady T, O'Loghlen A, Ochiya T, Olivier M, Ortiz A, Ortiz LA, Osteikoetxea X, Østergaard O, Ostrowski M, Park J, Pegtel DM, Peinado H, Perut F, Pfaffl MW, Phinney DG, Pieters BCH, Pink RC, Pisetsky DS, Pogge von Strandmann E, Polakovicova I, Poon IKH, Powell BH, Prada I, Pulliam L, Quesenberry P, Radeghieri A, Raffai RL, Raimondo S, Rak J, Ramirez MI, Raposo G, Rayyan MS, Regev-Rudzki N, Ricklefs FL, Robbins PD, Roberts DD, Rodrigues SC, Rohde E, Rome S, Rouschop KMA, Rughetti A, Russell AE, Saá P, Sahoo S, Salas-Huenuleo E, Sánchez C, Saugstad JA, Saul MJ, Schiffelers RM, Schneider R, Schøyen TH, Scott A, Shahaj E, Sharma S, Shatnyeva O, Shekari F, Shelke GV, Shetty AK, Shiba K, Siljander PRM, Silva AM, Skowronek A, Snyder OL, Soares RP, Sódar BW, Soekmadji C, Sotillo J, Stahl PD, Stoorvogel W, Stott SL, Strasser EF, Swift S, Tahara H, Tewari M, Timms K, Tiwari S, Tixeira R, Tkach M, Toh WS, Tomasini R, Torrecilhas AC, Tosar JP, Toxavidis V, Urbanelli L, Vader P, van Balkom BWM, van der Grein SG, Van Deun J, van Herwijnen MJC, Van Keuren-Jensen K, van Niel G, van Royen ME, van Wijnen AJ, Vasconcelos MH, Vechetti IJ, Veit TD, Vella LJ, Velot É, Verweij FJ, Vestad B, Viñas JL, Visnovitz T, Vukman KV, Wahlgren J, Watson DC, Wauben MHM, Weaver A, Webber JP, Weber V, Wehman AM, Weiss DJ, Welsh JA, Wendt S, Wheelock AM, Wiener Z, Witte L, Wolfram J, Xagorari A, Xander P, Xu J, Yan X, Yáñez-Mó M, Yin H, Yuana Y, Zappulli V, Zarubova J, Žėkas V, Zhang J-y, Zhao Z, Zheng L, Zheutlin AR, Zickler AM, Zimmermann P, Zivkovic AM, Zocco D, Zuba-Surma EK (2018) Minimal information for studies of extracellular vesicles 2018 (MISEV2018): a position statement of the International Society for Extracellular Vesicles and update of the MISEV2014 guidelines. Journal of Extracellular Vesicles;7(1):1535750 doi: 10.1080/20013078.2018.1535750

32. Lai RC, Arslan F, Lee MM, Sze NS, Choo A, Chen TS, Salto-Tellez M, Timmers L, Lee CN, El Oakley RM, Pasterkamp G, de Kleijn DP, Lim SK (2010) Exosome secreted by MSC reduces myocardial ischemia/reperfusion injury. Stem Cell Res;4(3):214-22 doi: 10.1016/j.scr.2009.12.003

33. Gyorgy B, Modos K, Pallinger E, Paloczi K, Pasztoi M, Misjak P, Deli MA, Sipos A, Szalai A, Voszka I, Polgar A, Toth K, Csete M, Nagy G, Gay S, Falus A, Kittel A, Buzas EI (2011) Detection and isolation of cell-derived microparticles are compromised

For Review Only

by protein complexes resulting from shared biophysical parameters. Blood;117(4):e39-48 doi: 10.1182/blood-2010-09-307595
34. Shao Y, Chen T, Zheng X, Yang S, Xu K, Chen X, Xu F, Wang L, Shen Y, Wang T, Zhang M, Hu W, Ye C, Yu X, Shao J, Zheng S (2018) Colorectal Cancer-derived Small Extracellular Vesicles Establish an Inflammatory Pre-metastatic Niche in Liver Metastasis. Carcinogenesis;39(11):1368-79 doi: 10.1093/carcin/bgy115
35. Chen ZY, He WZ, Peng LX, Jia WH, Guo RP, Xia LP, Qian CN (2015) A prognostic classifier consisting of 17 circulating cytokines is a novel predictor of overall survival for metastatic colorectal cancer patients. Int J Cancer;136(3):584-92 doi: 10.1002/ijc.29017
36. Chang PH, Pan YP, Fan CW, Tseng WK, Huang JS, Wu TH, Chou WC, Wang CH, Yeh KY (2016) Pretreatment serum interleukin-1beta, interleukin-6, and tumor necrosis factor-alpha levels predict the progression of colorectal cancer. Cancer Med;5(3):426-33 doi: 10.1002/cam4.602
37. Stanilov N, Miteva L, Dobreva Z, Stanilova S (2014) Colorectal cancer severity and survival in correlation with tumour necrosis factor-alpha. Biotechnol Biotechnol Equip;28(5):911-7 doi: 10.1080/13102818.2014.965047
38. Yuana Y, Sturk A, Nieuwland R (2013) Extracellular vesicles in physiological and pathological conditions. Blood Rev;27(1):31-9 doi: 10.1016/j.blre.2012.12.002
39. Kang DW, Choi CY, Cho YH, Tian H, Di Paolo G, Choi KY, Min do S (2015) Targeting phospholipase D1 attenuates intestinal tumorigenesis by controlling beta-catenin signaling in cancer-initiating cells. J Exp Med;212(8):1219-37 doi: 10.1084/jem.20141254
40. Takayama O, Yamamoto H, Damdinsuren B, Sugita Y, Ngan CY, Xu X, Tsujino T, Takemasa I, Ikeda M, Sekimoto M, Matsuura N, Monden M (2006) Expression of PPARdelta in multistage carcinogenesis of the colorectum: implications of malignant cancer morphology. Br J Cancer;95(7):889-95 doi: 10.1038/sj.bjc.6603343
41. Png CW, Weerasooriya M, Guo J, James SJ, Poh HM, Osato M, Flavell RA, Dong C, Yang H, Zhang Y (2016) DUSP10 regulates intestinal epithelial cell growth and colorectal tumorigenesis. Oncogene;35(2):206-17 doi: 10.1038/onc.2015.74
42. Pennel KAF, Park JH, McMillan DC, Roseweir AK, Edwards J (2019) Signal interaction between the tumour and inflammatory cells in patients with gastrointestinal cancer: Implications for treatment. Cell Signal;54:81-90 doi: 10.1016/j.cellsig.2018.11.013

For Review Only

Tables

Table 1. Gene expression in human primary monocytes given plasma extracellular vesicles (EVs). Comparison of monocytes incubated with plasma EVs from patients with rectal adenoma polyps (AP_{EV}) or invasive adenocarcinoma (RC_{EV}) who had either localized cancer (local-RC_{EV}) or metastatic disease (met-RC_{EV}). The data represent a *p*-value cut-off of 0.05 and fold-change cut-off of 1.2. **Upper table** The number of differentially expressed transcripts and analysis-ready molecules across observations eligible for Ingenuity® Pathway Analysis (IPA) software analysis. **Lower tables** Top five diseases and disorders in the core analysis, as defined by IPA, assigned to the transcriptional changes using Fisher´s Exact Test *p*-values and Benjamin-Hochberg (B-H) Multiple Testing Correction *p*-values.

	RC_{EV} vs AP_{EV}	met-RC_{EV} vs local-RC_{EV}
Differentially expressed transcripts	85	618
Analysis-ready molecules in IPA	73	588

Top 5 Molecular and cellular functions [a]	# transcripts	*p*-value range	B-H *p*-value range
RC_{EV} vs AP_{EV}			
Cancer	71	1.05E-06 – 3.92E-02	1.83E-03 – 1.35E-01
Organismal Injury and Abnormalities	71	1.05E-06 – 3.92E-02	1.83E-03 – 1.35E-01
Cell Morphology	12	3.05E-05 – 3.90E-02	1.78E-02 – 1.35E-01
Cellular Assembly and Organization	15	3.05E-05 – 3.90E-02	1.78E-02 – 1.35E-01
Organ Morphology	12	3.05E-05 – 3.79E-02	1.78E-02 – 1.34E-01
met-RC_{EV} vs local-RC_{EV}			
Cancer	539	5.21E-16 – 2.59E-02	2.41E-12 – 2.05E-01
Organismal Injury and Abnormalities	547	5.21E-16 – 2.59E-02	2.41E-12 – 2.05E-01
Endocrine System Disorders	425	3.05E-12 – 2.59E-02	2.53E-09 – 2.05E-01
Gastrointestinal Disease	472	1.37E-10 – 2.59E-02	6.68E-08 – 2.05E-01
Hematological Disease	162	5.06E-05 – 2.59E-02	1.35E-02 – 2.05E-01

[a] Functional core analysis. Gene expression differences in monocytes induced by the indicated groups. Eligible transcripts for IPA analysis were 15,052 of a total 16,598.

For Review Only

Figure legends

Figure 1. Characteristics of SW480-derived and plasma extracellular vesicles (EVs). A Cryo-electron microscopy images of small EVs ($SW480_{EV-S}$) and medium EVs ($SW480_{EV-M}$) from the colorectal cancer SW480 cell line and plasma EVs from a patient with localized rectal adenocarcinoma (RC_{EV}). Upper panel 25,000×, scale bars are 200 nm. Lower panel 80,000×, scale bars are 100 nm. **B** Immunoblot images of CD63, CD9, CD81, and GM130 protein expression. The samples were applied to different gels because of specific running requirements such as non-reducing conditions and overlapping molecular weight for the tetraspanins. A whole-cell lysate of the colorectal cancer LoVo cell line was included as positive control (pos ctr). **C** Nanoparticle tracking histograms of $SW480_{EV-S}$, $SW480_{EV-M}$, plasma EVs from a patient with rectal adenoma polyp (AP_{EV}), localized rectal adenocarcinoma (local-RC_{EV}) or metastatic disease (met-RC_{EV}). The data represent the mean ± standard error of the 3 videos from each sample. The data shown are representative for the groups.

For Review Only

Figure 2. Internalization of SW480-derived small extracellular vesicles (EVs) by human primary monocytes. **A** Confocal microscopy images of monocytes incubated with PKH67-labeled EVs (green). **B** Corresponding images of monocytes incubated with PKH67-labeled phosphate-buffered saline as negative control. **For all panels** Monocytes were fixed and mounted with the nuclear stain 4',6-diamidino-2-phenylindole (DAPI; blue). Upper panels show average intensity z-projections of a randomly selected monocyte. Lower panels display single z-planes spanning the cell in the upper panel. White dashed lines mark the plasma membrane. Scale bars are 5 µm.

For Review Only

Figure 3. Internalization of plasma extracellular vesicles (EVs) by human primary monocytes. A Confocal microscopy images of monocytes incubated with PKH67-labeled EVs (green) from a patient with rectal adenocarcinoma. **B** Corresponding images of monocytes incubated with PKH67-labeled EVs (green) from a patient with a rectal adenoma polyp. **C** Corresponding images of monocytes incubated with PKH67-labeled phosphate-buffered saline as negative control. **For all panels** Monocytes were fixed and mounted with the nuclear stain 4',6-diamidino-2-phenylindole (DAPI; blue). Upper panels show average intensity z-projections of randomly selected monocytes. Scale bars are 10 μm. Each red square denotes the cell shown in the corresponding lower panels, which display single z-planes spanning the denoted monocytes. White dashed lines mark the plasma membrane. Scale bars are 5 μm.

For Review Only

Figure 4. Cytokine secretion by human primary monocytes given SW480-derived and plasma extracellular vesicles (EVs). A Multiprotein (Luminex) assay results following incubation with colorectal cancer SW480 cell line medium EVs (EV-M) or small EVs (EV-S). The data represent the mean ± standard deviation of 3 independent experiments; * two-tailed Student's *t*-test $p<0.05$. **B** Luminex assay results following incubation with plasma EVs from patients with either a rectal adenoma polyp (AP_{EV}) ($n=6$), localized adenocarcinoma (local-RC_{EV}) ($n=11$), or metastatic disease (met-RC_{EV}) ($n=5$). Cytokine secretion by control monocytes given citrate phosphate-buffered saline (dotted line) or serum-free cell culture medium (stippled line).

For Review Only

Figure 5. Gene expression in human primary monocytes given plasma extracellular vesicles (EVs) and selected high-level functional categories. A Unsupervised hierarchical clustering of differentially expressed genes following incubation with plasma EVs from patients with rectal adenoma polyps (AP_{EV}; *n*=6) or invasive adenocarcinoma (RC_{EV}) who had either localized cancer (local-RC_{EV}; *n*=11) or metastatic disease (met-RC_{EV}; *n*=5). In the heat map, each column represents one patient and each row one transcript. Red colors indicate up-regulation and blue colors down-regulation of transcripts. **B** The number of significantly up-regulated (red) and down-regulated (blue) genes on comparison of the indicated groups. The data represent a one-way ANOVA *p*-value cut-off of 0.05 and a fold-change cut-off of 1.2. **C** Shared diseases and functions among the indicated groups, as predicted by Ingenuity® Pathway Analysis. The –log significance level is shown along the x-axis. The orange dotted line denotes the cut-off for significance, corresponding to Fisher´s Exact Test *p*-value 0.05. The figure in bracket behind each bar is the number of transcripts in the dataset involved in the given category. **D** Predicted activation state of function annotations significantly regulated in met-RC_{EV} compared to local-RC_{EV} by Ingenuity® Pathway Analysis, corresponding to Fisher´s Exact Test *p*-value 0.05. The z-score is shown along the x-axis, and a positive (red) or negative (blue) z-score indicates that the function is predicted to increase or decrease. The black dotted lines denote the cut-off for significance, corresponding to ≥ 2 and ≤ –2.

For Review Only

Supporting Information

Supplementary Table S1. Patient characteristics.

Supplementary Table S2. Biological processes in human primary monocytes given plasma extracellular vesicles (EVs) associated with the four jointly regulated transcripts across observations.

Supplementary Table S3. Biological processes in human primary monocytes given plasma extracellular vesicles (EVs) associated with the top ten up- or down-regulated transcripts.

Supplementary Table S4. Transcript IDs assigned to the top five diseases and disorders in Table 1.

Supplementary Table S5. Transcripts assigned to the additional diseases and functions in Figure 5c.

Supplementary Figure S1. Uptake of SW480-derived and plasma extracellular vesicles (EVs) by human primary monocytes.

Supplementary Figure S2. Internalization of SW480-derived small extracellular vesicles (EVs) by human primary monocytes.

Supplementary Figure S3. Internalization of plasma extracellular vesicles (EVs) by human primary monocytes.

Supplementary Figure S4. Cytokine secretion by human primary monocytes given SW480-derived extracellular vesicles.

For Review Only

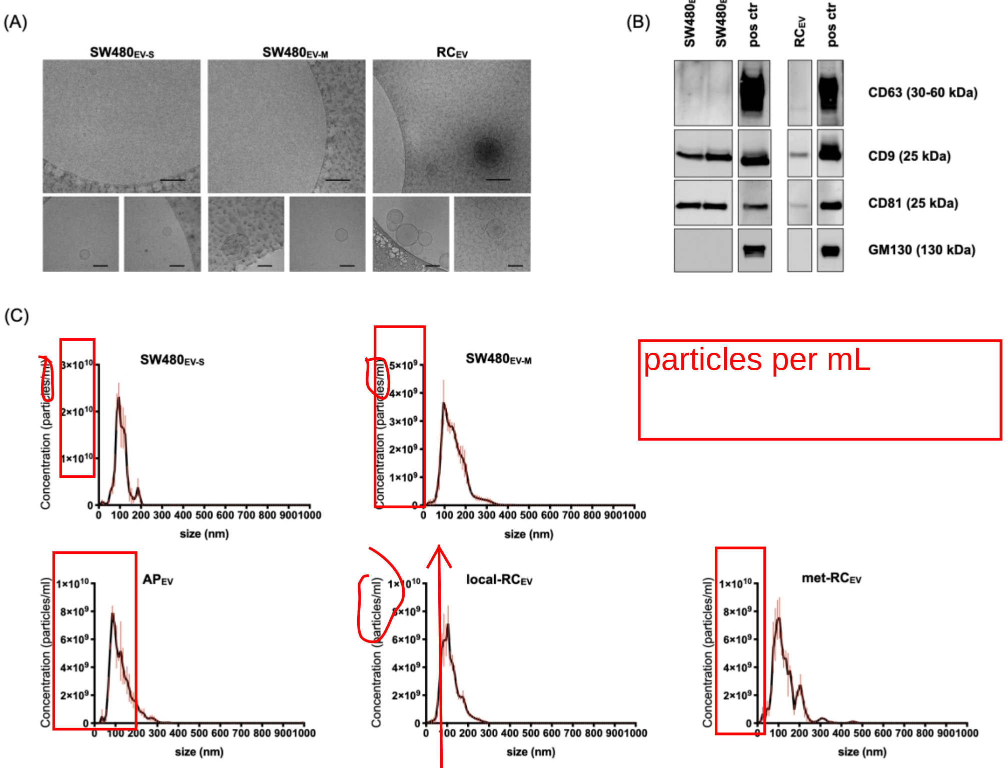

Figure 1

give thin space b/w
b.s. of operator

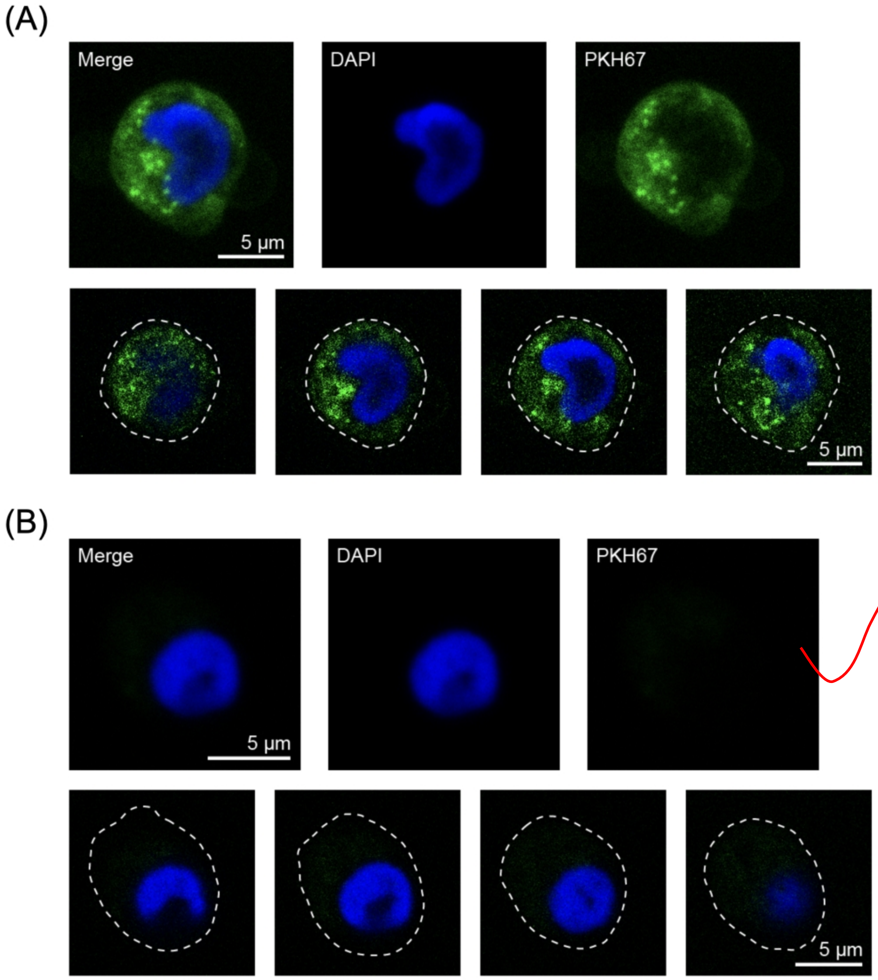

Figure 2

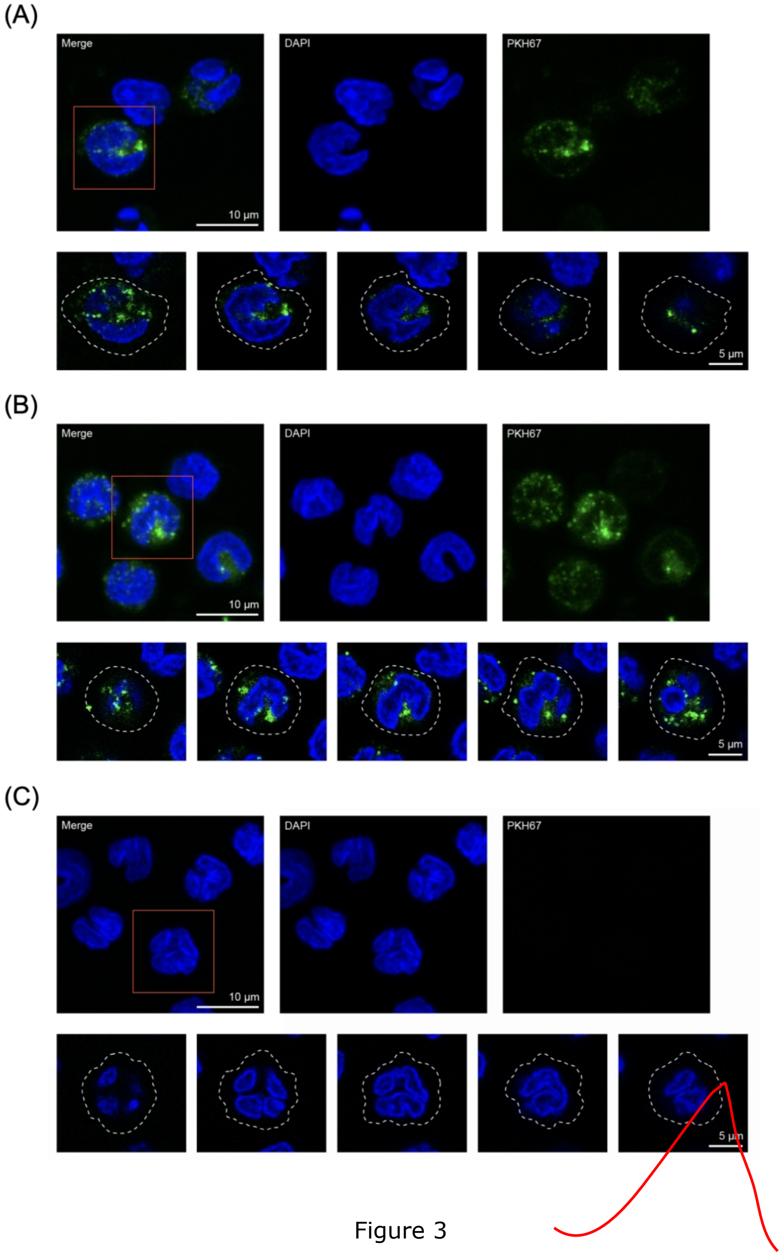
(A)
Merge
DAPI
PKH67
10 µm
5 µm
(B)
Merge
DAPI
PKH67
10 µm
5 µm
(C)
Merge
DAPI
PKH67
10 µm
5 µm

Figure 3

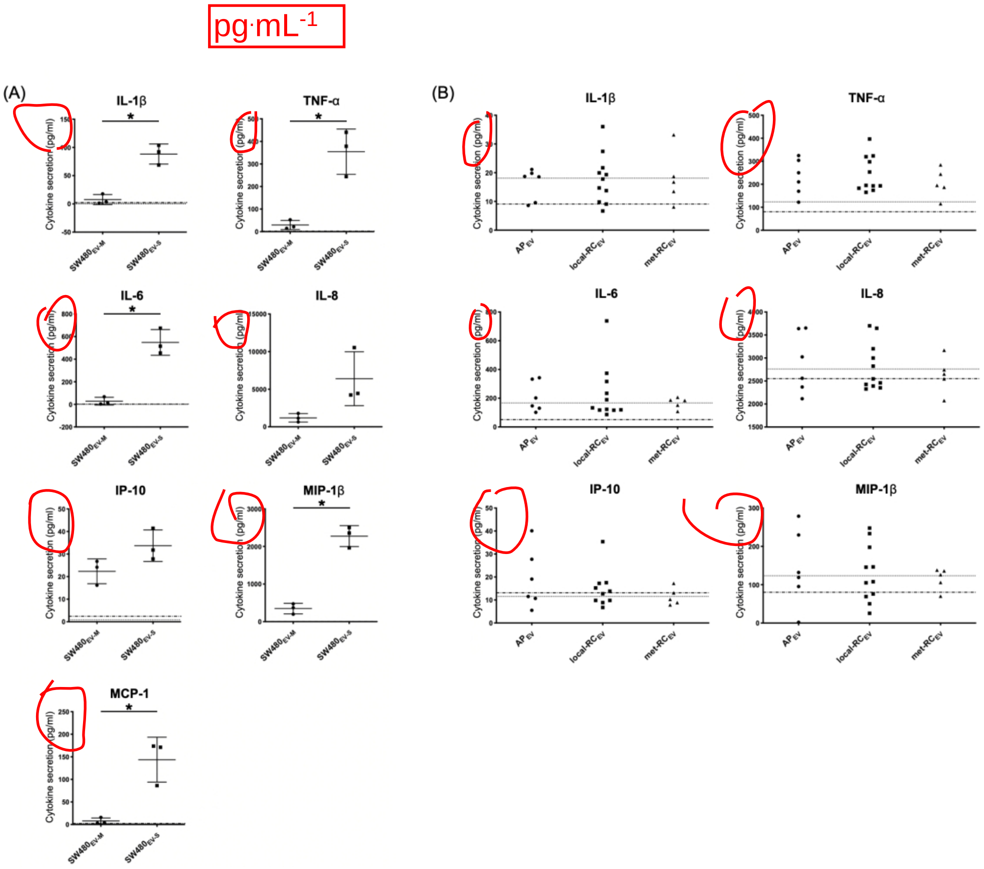
pg·mL-1
(A)
IL-1β
TNF-α
IL-6
IL-8
IP-10
MIP-1β
MCP-1
Cytokine secretion (pg/ml)
SW480EV-M
SW480EV-S
(B)
IL-1β
TNF-α
IL-6
IL-8
IP-10
MIP-1β
APEV
local-RCEV
met-RCEV

Figure 4

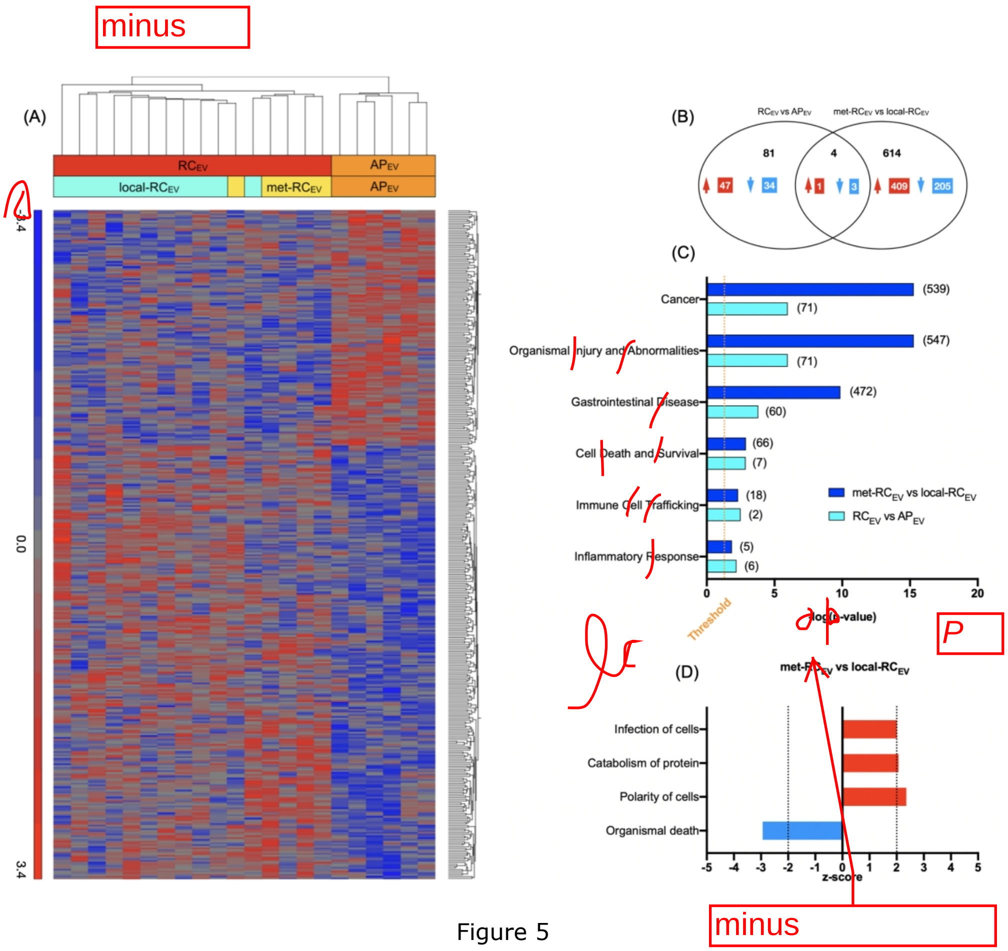
minus
(A)
RCEV
APEV
local-RCEV
met-RCEV
APEV
3.4
0.0
3.4
(B)
RCEV vs APEV
met-RCEV vs local-RCEV
81
4
614
47
34
1
3
409
205
(C)
Cancer
Organismal Injury and Abnormalities
Gastrointestinal Disease
Cell Death and Survival
Immune Cell Trafficking
Inflammatory Response
(539)
(71)
(547)
(71)
(472)
(60)
(66)
(7)
(18)
(2)
(5)
(6)
met-RCEV vs local-RCEV
RCEV vs APEV
Threshold
-log(p-value)
P
(D)
met-RCEV vs local-RCEV
Infection of cells
Catabolism of protein
Polarity of cells
Organismal death
z-score
minus

Figure 5

Supplementary Data

Supplementary Tables

Supplementary Table S1. Patient characteristics.

**Sex**	
Male	14 (63.6 %)
Female	8 (36.4%)
**Age**	
Median (range), years	56.5 (45-85)
**Disease**	
Adenoma polyp	6 (27.3%)
Adenocarcinoma	16 (72.7%)
**TNM-stage**	
T2-3	10 (62.5%)
T4	6 (37.5%)
N0	6 (37.5%)
N1-2	10 (62.5%)
M0	11 (68.8%)
M1	5 (31.3%)
**Distance from anal verge**	
Low (< 5 cm)	5 (31.3%)
Mid (5-9 cm)	7 (43,8%)
High (10-15 cm)	4 (25.0%)

TNM = Tumor-Node-Metastasis (for patients with cancer)

For Review Only

Supplementary Table S2. Biological processes in human primary monocytes given plasma extracellular vesicles (EVs) associated with the four jointly regulated transcripts across observations. Comparison of monocytes incubated with plasma EVs from patients with rectal adenoma polyps (AP_{EV}) or invasive adenocarcinoma (RC_{EV}) who had either localized cancer (local-RC_{EV}) or metastatic disease (met-RC_{EV}). The data represent a *p*-value cut-off of 0.05 and fold-change cut-off of 1.2. Biological processes (GO annotations), defined by Ingenuity® Pathway Analysis, assigned to the transcriptional changes by Fisher´s Exact Test *p*-values.

ID	Name	Fold-change	*p*-value	*Biological processes*
RC_{EV} vs AP_{EV} & met-RC_{EV} vs local-RC_{EV}				Up-regulated
USP32	Ubiquitin specific peptidase 32	1.218 & 1.247	4.58E-02 & 2.47E-02	protein deubiquitination; ubiquitin-dependent protein catabolic process
RC_{EV} vs AP_{EV} & met-RC_{EV} vs local-RC_{EV}				Down-regulated
LRRC3	Leucine rich repeat containing 3	-1.282 & -1.286	2.33E-03 & 1.02E-03	---
PPIF	Peptidylprolyl isomerase F	-1.208 & -1.285	2.01E-02 & 1.54E-03	apoptotic mitochondrial changes; apoptotic process; cellular response to arsenic-containing substance; cellular response to calcium ion; cellular response to hydrogen peroxide; necroptosis; negative regulation of apoptotic process; negative regulation of ATPase activity; negative regulation of intrinsic apoptotic signaling pathway; negative regulation of oxidative phosphorylation; negative regulation of oxidative phosphorylation uncoupler activity; negative regulation of release of cytochrome c from mitochondria; positive regulation of release of cytochrome c from mitochondria; programmed cell death; protein folding; protein peptidyl-prolyl isomerization; protein refolding; regulation of apoptotic process; regulation of mitochondrial membrane permeability; regulation of necrotic cell death; regulation of proton-transporting ATPase activity, rotational mechanism; response to ischemia; response to oxidative stress
RFTN1	Raftlin, lipid raft linker 1	-1.226 & -1.300	2.74E-02 & 8.83E-03	B cell receptor signaling pathway; dsRNA transport; interleukin-17 production; membrane raft assembly; positive regulation of growth rate; protein transport into membrane raft; response to exogenous dsRNA; T cell antigen processing and presentation; T cell receptor signaling pathway; toll-like receptor 3 signaling pathway

Supplementary Table S3. Biological processes in human primary monocytes given plasma extracellular vesicles (EVs) associated with the top ten up- or down-regulated transcripts. Comparison of monocytes incubated with plasma EVs from patients with rectal adenoma polyps (AP_{EV}) or invasive adenocarcinoma (RC_{EV}) who had either localized cancer (local-RC_{EV}) or metastatic disease (met-RC_{EV}). The data represent a *p*-value cut-off of 0.05 and fold-change cut-off of 1.2. Biological processes (GO annotations), defined by Ingenuity® Pathway Analysis, assigned to the transcriptional changes by Fisher´s Exact Test *p*-values.

ID	Name	Fold-change	*p*-value	*Biological process*
RC_{EV} vs AP_{EV}				Up-regulated
C8orf86	Chromosome 8 open reading frame 86	1.335	1.30E-04	---
USP54	Ubiquitin specific peptidase 54	1.291	1.17E-02	protein deubiquitination
EPS15	Epidermal growth factor receptor pathway substrate 15	1.272	7.74E-04	clathrin coat assembly; endocytic recycling; endocytosis; Golgi to endosome transport; membrane organization; negative regulation of epidermal growth factor receptor signaling pathway; positive regulation of receptor recycling; protein transport; regulation of cell proliferation; synaptic vesicle budding from presynaptic membrane; vesicle organization; viral entry into host cell; viral entry into host cell via receptor-mediated endocytosis
KCNMB3	Potassium calcium-activated channel subfamily M regulatory beta subunit 3	1.268	1.05E-02	detection of calcium ion; potassium ion transmembrane transport; potassium ion transport; regulation of action potential; regulation of action potential in neuron
ANGEL2	Angel homolog 2	1.265	2.26E-02	3'-UTR-mediated mRNA stabilization; negative regulation of mitotic cell cycle
TREM2	Triggering receptor expressed on myeloid cells 2	1.258	3.13E-02	apoptotic cell clearance; astrocyte activation; beta-amyloid clearance; cellular response to lipoteichoic acid; cellular response to peptidoglycan; defense response to bacterium; dendritic cell differentiation; detection of lipopolysaccharide; detection of lipoteichoic acid; detection of peptidoglycan; humoral immune response; innate immune response; lipopolysaccharide-mediated signaling pathway; microglial cell activation; microglial cell activation involved in immune response; negative regulation of apoptotic process; negative regulation of autophagy; negative regulation of interleukin-1 beta production; negative regulation of tumor necrosis factor production; osteoclast differentiation; phagocytosis, engulfment; positive regulation of antigen processing and presentation of peptide antigen via MHC class II; positive regulation of ATP biosynthetic process; positive regulation of beta-amyloid clearance;

For Review Only

				positive regulation of calcium-mediated signaling; positive regulation of CD40 signaling pathway; positive regulation of chemotaxis; positive regulation of ERK1 and ERK2 cascade; positive regulation of gene expression; positive regulation of interleukin-10 production; positive regulation of macrophage fusion; positive regulation of mitochondrion organization; positive regulation of osteoclast differentiation; positive regulation of peptidyl-tyrosine phosphorylation; positive regulation of phagocytosis, engulfment; positive regulation of protein phosphorylation; positive regulation of protein secretion; positive regulation of TOR signaling cascade; regulation of gene expression; regulation of immune response; regulation of innate immune response; regulation of interleukin-6 production; regulation of macrophage inflammatory protein 1 alpha production; regulation of peptidyl-tyrosine phosphorylation; regulation of resting membrane potential; regulation of TOR signaling cascade; response to ischemia
DPPA3	Developmental pluripotency associated 3	1.256	1.22E-02	chromatin organization; regulation of genetic imprinting
GNB5	G protein subunit beta 5	1.255	1.68E-02	dopamine receptor signaling pathway; G-protein coupled receptor signaling pathway; positive regulation of GTPase activity; protein folding; signal transduction
MLLT10	MLLT10 histone lysine methyltransferase DOT1L cofactor	1.254	3.89E-03	positive regulation of transcription from RNA polymerase II promoter
PIAS2	Protein inhibitor of activated STAT 2	1.251	2.98E-03	androgen receptor signaling pathway; negative regulation of androgen receptor signaling pathway; negative regulation of sequence-specific DNA binding transcription factor activity; positive regulation of dendrite morphogenesis; positive regulation of transcription, DNA-dependent; positive regulation of transcription from RNA polymerase II promoter; protein sumoylation; regulation of androgen receptor signaling pathway; regulation of osteoblast differentiation; regulation of transcription, DNA-dependent; regulation of transcription from RNA polymerase II promoter; response to organic substance; response to testosterone stimulus; spermatogenesis; transcription, DNA-dependent
RC_{EV} vs AP_{EV}				Down-regulated
KCNJ14	Potassium inwardly	-1.364	7.74E-05	cardiac conduction; ion transport; potassium

For Review Only

	rectifying channel subfamily J member 14			ion transport; regulation of ion transmembrane transport
VNN2	Vanin 2	-1.311	2.25E-02	pantothenate metabolic process
LOC100287036	Uncharacterized LOC100287036	-1.297	8.93E-03	---
TXNDC5	Thioredoxin domain containing 5	-1.292	3.55E-02	apoptotic cell clearance; cell redox homeostasis; negative regulation of apoptotic process; neutrophil degranulation
LRRC3	Leucine rich repeat containing 3	-1.282	2.33E-03	---
SPATA41	Spermatogenesis associated 41	-1.276	8.49E-04	---
ZNF785	Zinc finger protein 785	-1.270	1.19E-02	regulation of transcription from RNA polymerase II promoter
GRIN3B	Glutamate ionotropic receptor NMDA type subunit 3B	-1.270	1.11E-03	calcium ion transmembrane transport; ionotropic glutamate receptor signaling pathway; ion transport; protein insertion into membrane; regulation of calcium ion transport
TATDN3	TatD DNase domain containing 3	-1.256	2.35E-02	nucleic acid phosphodiester bond hydrolysis
LCE6A	C1orf44, late cornified envelope 6A	-1.250	2.43E-03	keratinization
met-RC_{EV} vs local-RC_{EV}				Up-regulated
FKBP2	FKBP prolyl isomerase 2	1.696	3.42E-02	protein peptidyl-prolyl isomerization
HK3	Hexokinase 3	1.662	4.72E-02	carbohydrate metabolic process; carbohydrate phosphorylation; cellular glucose homeostasis; glucose 6-phosphate metabolic process; glycolysis; hexose metabolic process; metabolic process; neutrophil degranulation; phosphorylation
IVNS1ABP	Influenza virus NS1A binding protein	1.618	2.87E-02	negative regulation of intrinsic apoptotic signaling pathway; negative regulation of protein ubiquitination; response to virus; RNA splicing; transcription from RNA polymerase III promoter; viral reproduction
ARL6IP6	ADP ribosylation factor like GTPase 6 interacting protein 6	1.586	3.09E-03	---
RPS6KA1	Ribosomal protein S6 kinase A1	1.553	3.28E-03	apoptotic process; cell cycle; hepatocyte proliferation; intracellular signal transduction; negative regulation of apoptotic process;

For Review Only

				negative regulation of cysteine-type endopeptidase activity involved in apoptotic process; phosphorylation; positive regulation of cell differentiation; positive regulation of cell growth; positive regulation of hepatic stellate cell activation; positive regulation of transcription, DNA-dependent; positive regulation of transcription from RNA polymerase II promoter; protein phosphorylation; regulation of DNA-dependent transcription in response to stress; regulation of translation in response to stress; signal transduction
OAZ2	Ornithine decarboxylase antizyme 2	1.553	3.34E-02	negative regulation of catalytic activity; polyamine biosynthetic process; polyamine metabolic process; positive regulation of intracellular protein transport; positive regulation of protein catabolic process; regulation of cellular amino acid metabolic process
SLC50A1	Solute carrier family 50 member 1	1.531	1.89E-02	carbohydrate transmembrane transport; carbohydrate transport; glucoside transport; hexose transport; positive regulation of gene expression, epigenetic
SLC37A2	Solute carrier family 37 member 2	1.496	1.89E-02	carbohydrate transport; glucose-6-phosphate transport; glycerol-3-phosphate metabolic process; glycerol-3-phosphate transport; glycerophosphate shuttle; hexose phosphate transport; organic anion transport; phosphate ion transmembrane transport; transmembrane transport
GNL1	G protein nucleolar 1 (putative)	1.492	2.49E-02	response to DNA damage stimulus; signal transduction; T cell mediated immunity
PANK2	Pantothenate kinase 2	1.490	1.83E-02	aerobic respiration; coenzyme A biosynthetic process; coenzyme biosynthetic process; mitochondrion morphogenesis; pantothenate metabolic process; phosphorylation; regulation of fatty acid metabolic process; regulation of mitochondrial membrane potential; regulation of triglyceride metabolic process; spermatid development
met-RC_{EV} vs local-RC_{EV}				Down-regulated
TRIM28	Tripartite motif containing 28	-1.798	3.12E-02	chromatin organization; convergent extension involved in axis elongation; DNA methylation involved in embryo development; DNA repair; embryo implantation; embryonic placenta morphogenesis; epithelial to mesenchymal transition; innate immune response; in utero embryonic development; negative regulation of retroviral genome replication; negative regulation of transcription, DNA-dependent; negative regulation of transcription from RNA

For Review Only

				polymerase II promoter; positive regulation of DNA binding; positive regulation of DNA repair; positive regulation of methylation-dependent chromatin silencing; positive regulation of protein import into nucleus; positive regulation of transcription, DNA-dependent; protein autophosphorylation; protein oligomerization; protein phosphorylation; protein sumoylation; protein ubiquitination; Ras protein signal transduction; regulation of genetic imprinting; transcription initiation from RNA polymerase II promoter; viral reproduction
EMD	Emerin	-1.749	2.08E-02	cellular response to growth factor stimulus; mitotic nuclear envelope reassembly; muscle contraction; muscle organ development; negative regulation of canonical Wnt receptor signaling pathway; negative regulation of fibroblast proliferation; nuclear envelope reassembly; positive regulation of protein export from nucleus; regulation of canonical Wnt receptor signaling pathway; skeletal muscle cell differentiation
CYBC1	Cytochrome b-245 chaperone 1	-1.671	4.02E-02	immune system process; innate immune response; respiratory burst after phagocytosis
GNAI1	G protein subunit alpha i1	-1.412	1.48E-02	adenylate cyclase-inhibiting G-protein coupled receptor signaling pathway; adenylate cyclase-modulating G-protein coupled receptor signaling pathway; cell cycle; cell division; G-protein coupled receptor signaling pathway; negative regulation of synaptic transmission; protein folding; regulation of cAMP-mediated signaling; regulation of mitotic spindle organization; response to peptide hormone stimulus; signal transduction
HBEGF	Heparin binding EGF like growth factor	-1.392	3.85E-02	angiogenesis; blastocyst growth; cell chemotaxis; cell migration; epidermal growth factor receptor signaling pathway; ERBB2 signaling pathway; MAPK cascade; membrane organization; muscle organ development; negative regulation of elastin biosynthetic process; negative regulation of epidermal growth factor receptor signaling pathway; nervous system development; positive regulation of cell growth; positive regulation of cell migration; positive regulation of cell proliferation; positive regulation of keratinocyte migration; positive regulation of peptidyl-tyrosine phosphorylation; positive regulation of protein kinase B signaling cascade; positive regulation of smooth muscle cell proliferation; positive regulation of wound healing; regulation of cell motility; regulation of heart contraction; regulation of receptor activity; signal transduction; wound healing, spreading of

For Review Only

				epidermal cells
CD3E	CD3e molecule	-1.380	8.57E-03	adaptive immune response; apoptotic signaling pathway; cell surface receptor signaling pathway; cerebellum development; dendrite development; G-protein coupled receptor signaling pathway; immune system process; lymphocyte activation; macromolecular complex assembly; negative regulation of gene expression; negative regulation of smoothened signaling pathway; negative thymic T cell selection; positive regulation of alpha-beta T cell proliferation; positive regulation of calcium-mediated signaling; positive regulation of cell-cell adhesion mediated by integrin; positive regulation of cell-matrix adhesion; positive regulation of gene expression; positive regulation of interferon-gamma production; positive regulation of interleukin-2 biosynthetic process; positive regulation of interleukin-4 production; positive regulation of peptidyl-tyrosine phosphorylation; positive regulation of T cell activation; positive regulation of T cell anergy; positive regulation of T cell proliferation; positive thymic T cell selection; protein homooligomerization; regulation of apoptotic process; regulation of immune response; response to nutrient; signal complex assembly; T cell activation; T cell costimulation; T cell differentiation; T cell differentiation in thymus; T cell receptor signaling pathway; transmembrane receptor protein tyrosine kinase signaling pathway
VAV2	Vav guanine nucleotide exchange factor 2	-1.371	1.08E-02	angiogenesis; cell migration; cell projection assembly; ephrin receptor signaling pathway; Fc-epsilon receptor signaling pathway; Fc-gamma receptor signaling pathway involved in phagocytosis; G-protein coupled receptor signaling pathway; intracellular signal transduction; lamellipodium assembly; platelet activation; positive regulation of apoptotic process; positive regulation of phosphatidylinositol 3-kinase activity; regulation of cell size; regulation of GTPase activity; regulation of Rho protein signal transduction; regulation of small GTPase mediated signal transduction; signal transduction; small GTPase mediated signal transduction; vascular endothelial growth factor receptor signaling pathway
MIA	MIA SH3 domain containing	-1.358	1.71E-02	cell-matrix adhesion; extracellular matrix organization; regulation of receptor activity; signal transduction
GNA12	G protein subunit alpha 12	-1.357	4.55E-02	adenylate cyclase-modulating G-protein coupled receptor signaling pathway; blood coagulation; cell differentiation; dopamine

For Review Only

				receptor signaling pathway; embryonic digit morphogenesis; G-protein coupled receptor signaling pathway; intracellular signal transduction; in utero embryonic development; platelet activation; regulation of cell shape; regulation of fibroblast migration; regulation of proteasomal ubiquitin-dependent protein catabolic process; regulation of TOR signaling cascade; response to drug; Rho protein signal transduction; signal transduction
FARSB	Phenylalanyl-tRNA synthetase subunit beta	-1.350	2.74E-02	phenylalanyl-tRNA aminoacylation; protein heterotetramerization; translation; tRNA aminoacylation for protein translation

For Review Only

Supplementary Table S4. Transcript IDs assigned to the top five diseases and disorders in Table 1. Comparison of monocytes incubated with plasma extracellular vesicles (EVs) from patients with rectal adenoma polyps (AP_{EV}) or invasive adenocarcinoma (RC_{EV}) who had either localized cancer (local-RC_{EV}) or metastatic disease (met-RC_{EV}). The data represent a *p*-value cut-off of 0.05 and fold-change cut-off of 1.2.

Top 5 Diseases and Disorders	ID
RC_{EV} vs AP_{EV}	
Cancer	ACTR3B, ANGEL2, ARG2, ARIH2OS, ATXN3, C8orf86, CHST12, DCLRE1C, DDR2, DDX51, DERL2, DGKD, DPPA3, EFCAB6, EPS15, EZHIP, FAM30A, GAN, GART, GNB5, GOLIM4, GRIN3B, HCN4, IRX3, KBTBD12, KCNJ14, KCNMB3, KRTAP12-4, LCE6A, LIN7A, LRRC3, LYRM4, MEAK7, MLLT10, MRPS6, NEB, NPHP3, PALM, PIAS2, PIGP, PLA2G4A, PLD1, PNMA2, PPARGC1A, PPIF, RASSF8, RFTN1, RORC, SCIN, SNX21, SPAG11B, TADA1, TATDN3, TDRD3, TESK2, TM4SF1, TMEM185A, TPRG1, TREM2, TRIM62, TRIM63, TSPAN12, TXNDC5, USP1, USP32, USP54, VASN, VNN2, ZFAND1, ZFP62, ZNF785
Organismal Injury and Abnormalities	ACTR3B, ANGEL2, ARG2, ARIH2OS, ATXN3, C8orf86, CHST12, DCLRE1C, DDR2, DDX51, DERL2, DGKD, DPPA3, EFCAB6, EPS15, EZHIP, FAM30A, GAN, GART, GNB5, GOLIM4, GRIN3B, HCN4, IRX3, KBTBD12, KCNJ14, KCNMB3, KRTAP12-4, LCE6A, LIN7A, LRRC3, LYRM4, MEAK7, MLLT10, MRPS6, NEB, NPHP3, PALM, PIAS2, PIGP, PLA2G4A, PLD1, PNMA2, PPARGC1A, PPIF, RASSF8, RFTN1, RORC, SCIN, SNX21, SPAG11B, TADA1, TATDN3, TDRD3, TESK2, TM4SF1, TMEM185A, TPRG1, TREM2, TRIM62, TRIM63, TSPAN12, TXNDC5, USP1, USP32, USP54, VASN, VNN2, ZFAND1, ZFP62, ZNF785
Cell Morphology	ATXN3, DCLRE1C, EPS15, NEB, PALM, PLA2G4A, PLD1, PPARGC1A, RORC, SCIN, TM4SF1, TRIM63
Cellular Assembly and Organization	ATXN3, DGKD, EPS15, GAN, LIN7A, NEB, PALM, PLA2G4A, PLD1, PPARGC1A, PPIF, RORC, SCIN, TM4SF1, TRIM63
Organ Morphology	ARG2, ATXN3, DCLRE1C, IRX3, NEB, PLA2G4A, PLD1, PPARGC1A, RFTN1, RORC, TREM2, TRIM63
met-RC_{EV} vs local-RC_{EV}	
Cancer	AAMP, ABCB7, ABHD12, ACADVL, ACLY, ACOT8, ACOXL, ACSF3, ACTN4, ADAMTS12, ADAMTS2, ADORA2B, ADPRH, ADSS2, AEBP1, AHNAK, AKAP8, ALG12, ANGPTL2, ANKRD13B, ANKS1A, AOC1, AP1G2, APAF1, APBB3, APOBEC2, APOBR, APOE, APTX, AQP12A/AQP12B, AQP2, ARC, ARFGAP1, ARFIP2, ARFRP1, ARHGAP35, ARHGEF11, ARHGEF39, ARHGEF40, ARL6IP6, ARV1, ATE1, ATG14, ATP13A1, ATP1A1, ATP1A3, ATRAID, ATXN7L2, AWAT1, AZIN2, BBS7, BCAS1, BCL2L14, BCL7A, BCORL1, BMS1, BST1, C11orf68, C16orf70, C22orf34, C6orf136, CA3, CACNA1B, CAD, CALCOCO1, CALML4, CAMK2N1, CAPG, CASP10, CASP8, CASR, CBY2, CCDC142, CCDC166, CCDC81, CCDC84, CCDC88B, CCNF, CCSAP, CD14, CD3E, CDH15, CDX4, CEACAM21, CEP85, CERS5, CHCHD4, CHKB, CHTOP, CLCN6, CLCNKB, CLDN23, CLIC3, CLN8, CMSS1, CMTM1, CNGA2, CNGB1, CNNM2, CNTROB, COG1, COG8,

For Review Only

COL9A1, COQ7, COX18, CPLANE1, CRNN, CRYBA2, CRYGS, CSAG2/CSAG3, CTCF, CTSH, CTSV, CTTNBP2, CYP2S1, CYP4A22, CYREN, CYS1, D2HGDH, DDAH2, DDIT4, DDX56, DEPDC7, DEPTOR, DHRS3, DHX33, DIPK1B, DLGAP1, DMAC2L, DMAP1, DNAAF3, DNAJC16, DNMT3A, DPP6, DTNBP1, DUS3L, EEF1AKNMT, EEF1G, EGF, EIF4E2, ELMOD3, EMC9, EMD, EML4, EN2, ENDOU, ENTPD2, EPHB2, ERP29, ESM1, ESPN, EXOC3L2, FABP5, FAM111A, FAM214B, FAM72C/FAM72D, FARSB, FBXL20, FBXO38, FEM1A, FHL3, FILIP1L, FKBP2, FKBP5, FKBPL, FLI1, FNTB, FRAT2, GALNT12, GAS1, GAS7, GBA2, GBGT1, GEN1, GFI1, GJA3, GLI1, GNA12, GNAI1, GNAT1, GNL1, GNL2, GPAT2, GPAT4, GPR141, GRHL2, GRK6, GTF2H4, GTPBP1, GTPBP2, H2BC10, HAMP, HAUS3, HBEGF, HDHD3, HEATR5A, HIBADH, HIF1AN, HK1, HK3, HNRNPCL1/HNRNPCL2, HNRNPF, HOMER2, HSCB, HSD17B10, HSPA8, HSPB6, HTRA1, HTRA2, HVCN1, IDH3G, IFFO1, IGF1R, IGF2BP1, IGLC1, IGSF21, IKBKE, IL1R2, IL1RL2, INO80C, INPP5D, INPPL1, INTS10, IRGQ, ISY1, ITGAL, ITPA, IVNS1ABP, JADE3, JUN, KCNA7, KCNJ11, KCNJ15, KCTD13, KHNYN, KIAA0513, KIF1C, KLHDC3, KLHL6, KLK10, KLK6, KNOP1, KRT16, KRT38, KRT6C, LAMB4, LAT2, LCE1B, LCE2B, LCE4A, LDHC, LDHD, LDLRAP1, LEFTY2, LETMD1, LGALS2, LMF1, LOC102724159/PWP2, LOXHD1, LRRC3, LRRC47, LRRC57, LRRC8A, LRRFIP1, LSM14A, LUC7L, LUC7L2, MAN1A1, MAN2A2, MANBAL, MAP1LC3A, MAP3K10, MARCHF5, MARVELD1, MBLAC1, MBTPS1, MCM5, MDN1, MED24, METTL7B, MIA, MICOS10-NBL1/NBL1, MLXIP, MOCS2, MPHOSPH8, MRAS, MRPL13, MRPL37, MSRB2, MYBL2, MYH11, MYH7, MYL1, MYL9, MYLIP, MYO15A, MYO1C, MYO5C, MYOZ3, NAA10, NAA40, NAGA, NDST1, NDUFAF4, NDUFAF7, NDUFS7, NEDD4L, NEK4, NFIC, NGB, NLRP12, NODAL, NPR1, NR2C2AP, NR4A2, NSD1, NSUN6, NTAN1, NUDT9, NUMA1, OAZ2, OGDH, OGG1, OPN1MW (includes others), OR6N1, ORMDL3, OSGIN1, OTOP2, OXLD1, PANK2, PANK4, PAPSS1, PARP8, PCIF1, PDIK1L, PEBP4, PELP1, PEX19, PFDN1, PGRMC2, PHF19, PI4KA, PI4KAP2, PIEZO1, PIGU, PLA2G2A, PNPLA2, PNPLA6, POLB, PPARD, PPFIBP2, PPIF, PPP1R21, PRDM10, PRDM8, PRKCD, PRKCQ, PRKX, PRMT2, PRNP, PROCR, PRPSAP1, PSD4, PSMB6, PTHLH, PTK2B, PTPRN2, PXDC1, PYROXD2, QPCTL, RAB11B, RAB35, RAB5B, RABEPK, RABL2A, RACGAP1, RALGPS1, RARRES2, RASA4, RBM12B, RBM41, RCC2, RD3, REEP4, REXO2, RFTN1, RGP1, RIF1, RING1, RIPK4, RNF112, RNF123, RNF224, RNF5, RNFT1, RPS6KA1, RSPH10B/RSPH10B2, RXRA, S100A2, SAMD11, SART3, SCAMP4, SEC22A, SEPHS2, SEPTIN10, SETD1A, SF3A3, SFMBT1, SGSM3, SH2D2A, SHPRH, SHROOM3, SIPA1L2, SLA2, SLAIN1, SLC17A7, SLC1A7, SLC25A11, SLC25A19, SLC25A33, SLC25A46, SLC2A13, SLC2A6, SLC2A8, SLC35A1, SLC37A2, SLC38A7, SLC41A1, SLC43A2, SLC46A2, SLC50A1, SLC8A2, SLC9A3R1, SLCO4A1, SMC3, SMG5, SMOC1, SNAPIN, SNX11, SNX24, SNX33, SOCS2, SOCS6, SORD, SOX14, SPATA31A6 (includes others), SPOP, SPSB2, SPTLC2, SRR, SSBP3, STK11IP, STKLD1, SUDS3, SULT1A3/SULT1A4, SURF4, SYNJ2, SYT2, SYTL4, TARBP2, TBC1D10B, TBC1D2, TBCE, TCEAL3, TCERG1L, TCF20, TCFL5, TCP11L2, TEP1, TET3, TK1, TLX2, TMCO4, TMEM159, TMEM181, TMEM182, TMEM211, TMEM233, TMEM260, TMEM39B, TMEM88, TMOD1, TNFRSF10D, TOP3B, TRAF7, TRAPPC4, TRIB3, TRIM28, TRIM37, TRIP10, TRMT2B, TSC22D4, TSR2, TTC36, TTC7A, TXLNA, TXNRD2, UAP1L1, UBIAD1, UBQLN3, UCK1, UCN2, UPF3B, URGCP, USP21, USP32, USP5, USP53, VAMP1, VAV2, VPS33B, VPS37D, WDR46, WDR55, WNT8B, WRAP73, XIRP1, XRCC4, YIPF2, YPEL1, ZBTB2, ZDHHC3, ZFYVE21, ZNF154, ZNF175, ZNF276, ZNF329, ZNF341, ZNF420, ZNF426, ZNF653, ZNF721, ZNF75D, ZNF775, ZNF780A, ZNF784, ZNF81, ZNRF3, ZSWIM8

For Review Only

Organismal Injury and Abnormalities	AAMP, ABCB7, ABHD12, ACADVL, ACLY, ACOT8, ACOXL, ACSF3, ACTN4, ADA2, ADAMTS12, ADAMTS2, ADORA2B, ADPRH, ADSS2, AEBP1, AHNAK, AKAP8, ALG12, ANGPTL2, ANKRD13B, ANKS1A, AOC1, AP1G2, APAF1, APBB3, APOBEC2, APOBR, APOE, APTX, AQP12A/AQP12B, AQP2, ARC, ARFGAP1, ARFIP2, ARFRP1, ARHGAP35, ARHGEF11, ARHGEF39, ARHGEF40, ARL6IP6, ARV1, ATE1, ATG14, ATP13A1, ATP1A1, ATP1A3, ATRAID, ATXN7L2, AWAT1, AZIN2, BBS7, BCAS1, BCL2L14, BCL7A, BCORL1, BMS1, BST1, C11orf68, C16orf70, C22orf34, C6orf136, CA3, CACNA1B, CAD, CALCOCO1, CALML4, CAMK2N1, CAPG, CASP10, CASP8, CASR, CBY2, CCDC142, CCDC166, CCDC81, CCDC84, CCDC88B, CCNF, CCSAP, CD14, CD3E, CDH15, CDX4, CEACAM21, CEP85, CERS5, CHCHD4, CHKB, CHTOP, CLCN6, CLCNKB, CLDN23, CLIC3, CLN8, CMSS1, CMTM1, CNGA2, CNGB1, CNNM2, CNTROB, COG1, COG8, COL9A1, COQ7, COX18, CPLANE1, CRNN, CRYBA2, CRYGS, CSAG2/CSAG3, CTCF, CTSH, CTSV, CTTNBP2, CYP2S1, CYP4A22, CYREN, CYS1, D2HGDH, DDAH2, DDIT4, DDX56, DEPDC7, DEPTOR, DHRS3, DHX33, DIPK1B, DLGAP1, DMAC2L, DMAP1, DNAAF3, DNAJC16, DNMT3A, DPP6, DTNBP1, DUS3L, EEF1AKNMT, EEF1G, EGF, EIF4E2, ELMOD3, ELOA, ELP1, EMC9, EMD, EML4, EN2, ENDOU, ENTPD2, EPHB2, ERP29, ESM1, ESPN, EXOC3L2, FABP5, FAM111A, FAM214B, FAM72C/FAM72D, FARSB, FBXL20, FBXO38, FEM1A, FHL3, FILIP1L, FKBP2, FKBP5, FKBPL, FLI1, FNTB, FRAT2, GALNT12, GAS1, GAS7, GBA2, GBGT1, GEN1, GFI1, GJA3, GLI1, GMPPB, GNA12, GNAI1, GNAT1, GNL1, GNL2, GPAT2, GPAT4, GPR141, GPR179, GRHL2, GRK6, GTF2H4, GTPBP1, GTPBP2, GUCY1B1, H2BC10, HAMP, HAUS3, HBEGF, HDHD3, HEATR5A, HIBADH, HIF1AN, HK1, HK3, HNRNPCL1/HNRNPCL2, HNRNPF, HOMER2, HSCB, HSD17B10, HSPA8, HSPB6, HTRA1, HTRA2, HVCN1, IDH3G, IFFO1, IGF1R, IGF2BP1, IGLC1, IGSF21, IKBKE, IL1R2, IL1RL2, INO80C, INPP5D, INPPL1, INTS10, IRGQ, ISY1, ITGAL, ITPA, IVNS1ABP, JADE3, JUN, KCNA7, KCNE3, KCNJ11, KCNJ15, KCTD13, KHNYN, KIAA0513, KIF1C, KLHDC3, KLHL6, KLK10, KLK6, KNOP1, KRT16, KRT38, KRT6C, LAMB4, LAT2, LCE1B, LCE2B, LCE4A, LDHC, LDHD, LDLRAP1, LEFTY2, LETMD1, LGALS2, LMF1, LOC102724159/PWP2, LOXHD1, LRRC3, LRRC47, LRRC57, LRRC8A, LRRFIP1, LSM14A, LUC7L, LUC7L2, MAN1A1, MAN2A2, MANBAL, MAP1LC3A, MAP3K10, MARCHF5, MARVELD1, MBLAC1, MBTPS1, MCM5, MDN1, MED24, METTL7B, MIA, MICOS10-NBL1/NBL1, MLXIP, MOCS2, MPHOSPH8, MRAS, MRPL13, MRPL37, MSRB2, MYBL2, MYH11, MYH7, MYL1, MYL9, MYLIP, MYO15A, MYO1C, MYO5C, MYOZ3, NAA10, NAA40, NAGA, NDST1, NDUFAF4, NDUFAF7, NDUFS7, NEDD4L, NEK4, NFIC, NGB, NLRP12, NODAL, NPR1, NR2C2AP, NR4A2, NSD1, NSUN6, NTAN1, NUDT9, NUMA1, OAZ2, OGDH, OGG1, OPN1MW (includes others), OR6N1, ORMDL3, OSGIN1, OTOP2, OXLD1, PANK2, PANK4, PAPSS1, PARP8, PCIF1, PDIK1L, PEBP4, PELP1, PEX19, PFDN1, PGRMC2, PHF19, PI4KA, PI4KAP2, PIEZO1, PIGU, PLA2G2A, PNPLA2, PNPLA6, POLB, PPARD, PPFIBP2, PPIF, PPP1R21, PRCD, PRDM10, PRDM8, PRKCD, PRKCQ, PRKX, PRMT2, PRNP, PROCR, PRPSAP1, PSD4, PSMB6, PTHLH, PTK2B, PTPRN2, PXDC1, PYROXD2, QPCTL, RAB11B, RAB35, RAB5B, RABEPK, RABL2A, RACGAP1, RALGPS1, RARRES2, RASA4, RBM12B, RBM41, RCC2, RD3, REEP4, REXO2, RFTN1, RGP1, RIF1, RING1, RIPK4, RNF112, RNF123, RNF224, RNF5, RNFT1, RPS6KA1, RSPH10B/RSPH10B2, RXRA, S100A2, SAMD11, SART3, SCAMP4, SEC22A, SEPHS2, SEPTIN10, SETD1A, SF3A3, SFMBT1, SGSM3, SH2D2A, SHPRH, SHROOM3, SIPA1L2, SLA2, SLAIN1, SLC17A7, SLC1A7, SLC25A11, SLC25A19, SLC25A33, SLC25A46, SLC2A13, SLC2A6, SLC2A8,

For Review Only

	SLC35A1, SLC37A2, SLC38A7, SLC41A1, SLC43A2, SLC46A2, SLC50A1, SLC8A2, SLC9A3R1, SLCO4A1, SMC3, SMG5, SMOC1, SNAPIN, SNX11, SNX24, SNX33, SOCS2, SOCS6, SORD, SOX14, SPATA31A6 (includes others), SPOP, SPSB2, SPTLC2, SRR, SSBP3, STK11IP, STKLD1, SUDS3, SULT1A3/SULT1A4, SURF4, SYNJ2, SYT2, SYTL4, TARBP2, TBC1D10B, TBC1D2, TBCE, TCEAL3, TCERG1L, TCF20, TCFL5, TCP11L2, TEP1, TET3, TK1, TLX2, TMCO4, TMEM159, TMEM181, TMEM182, TMEM211, TMEM233, TMEM260, TMEM39B, TMEM88, TMOD1, TNFRSF10D, TOP3B, TRAF7, TRAPPC4, TRIB3, TRIM28, TRIM37, TRIP10, TRMT2B, TSC22D4, TSR2, TTC36, TTC7A, TXLNA, TXNRD2, UAP1L1, UBIAD1, UBQLN3, UCK1, UCN2, UPF3B, URGCP, USP21, USP32, USP5, USP53, VAMP1, VAV2, VPS33B, VPS37D, WDR46, WDR55, WNT8B, WRAP73, XIRP1, XRCC4, YIPF2, YPEL1, ZBTB2, ZDHHC3, ZFYVE21, ZNF154, ZNF175, ZNF276, ZNF329, ZNF341, ZNF420, ZNF426, ZNF653, ZNF721, ZNF75D, ZNF775, ZNF780A, ZNF784, ZNF81, ZNRF3, ZSWIM8
Endocrine System Disorders	AAMP, ABCB7, ABHD12, ACADVL, ACLY, ACSF3, ACTN4, ADAMTS12, ADAMTS2, ADORA2B, ADSS2, AEBP1, AHNAK, ALG12, ANGPTL2, ANKRD13B, ANKS1A, AP1G2, APAF1, APBB3, APOBR, APOE, APTX, AQP12A/AQP12B, AQP2, ARC, ARFGAP1, ARFIP2, ARFRP1, ARHGAP35, ARHGEF11, ARHGEF39, ARHGEF40, ARV1, ATE1, ATG14, ATP13A1, ATP1A1, ATP1A3, ATRAID, ATXN7L2, AWAT1, BBS7, BCAS1, BCL7A, BCORL1, BMS1, BST1, C11orf68, C16orf70, C6orf136, CA3, CACNA1B, CAD, CALML4, CAMK2N1, CAPG, CASP10, CASP8, CASR, CBY2, CCDC142, CCDC166, CCDC81, CCDC84, CCDC88B, CCNF, CCSAP, CD3E, CDH15, CDX4, CEP85, CHKB, CLCN6, CLCNKB, CLDN23, CLIC3, CNGA2, CNGB1, CNNM2, CNTROB, COG1, COG8, COL9A1, COX18, CPLANE1, CRYGS, CTCF, CTSH, CTSV, CTTNBP2, CYP2S1, D2HGDH, DDAH2, DDX56, DEPDC7, DEPTOR, DHRS3, DHX33, DIPK1B, DLGAP1, DMAC2L, DNAAF3, DNAJC16, DNMT3A, DPP6, DTNBP1, DUS3L, EEF1AKNMT, EEF1G, EGF, ELMOD3, EMC9, EMD, EML4, EN2, ENDOU, ENTPD2, EPHB2, ERP29, ESM1, ESPN, EXOC3L2, FABP5, FAM111A, FAM214B, FARSB, FBXL20, FBXO38, FEM1A, FILIP1L, FKBP2, FKBP5, FLI1, FNTB, FRAT2, GALNT12, GAS1, GAS7, GBA2, GEN1, GFI1, GJA3, GLI1, GNA12, GNAI1, GNL1, GNL2, GPAT2, GRHL2, GRK6, GTF2H4, GTPBP1, GTPBP2, HAUS3, HBEGF, HDHD3, HEATR5A, HIBADH, HIF1AN, HNRNPCL1/HNRNPCL2, HNRNPF, HOMER2, HSCB, HSPB6, HTRA1, HTRA2, IDH3G, IFFO1, IGF1R, IGSF21, IKBKE, IL1RL2, INPP5D, INPPL1, INTS10, IRGQ, ISY1, ITGAL, IVNS1ABP, KCNA7, KCNJ11, KCTD13, KHNYN, KIAA0513, KIF1C, KLHL6, KLK10, KLK6, KNOP1, KRT16, KRT38, KRT6C, LAMB4, LCE4A, LDHD, LDLRAP1, LEFTY2, LMF1, LOC102724159/PWP2, LOXHD1, LRRC3, LRRC47, LRRC8A, LRRFIP1, LSM14A, MAN1A1, MAN2A2, MAP1LC3A, MAP3K10, MARCHF5, MARVELD1, MBLAC1, MBTPS1, MCM5, MDN1, MED24, METTL7B, MIA, MICOS10-NBL1/NBL1, MPHOSPH8, MRPL13, MSRB2, MYBL2, MYH11, MYH7, MYL1, MYL9, MYLIP, MYO15A, MYO1C, MYO5C, MYOZ3, NAA10, NAGA, NDST1, NDUFAF7, NDUFS7, NEDD4L, NEK4, NFIC, NLRP12, NODAL, NPR1, NR4A2, NSD1, NSUN6, NUMA1, OGDH, OGG1, OTOP2, PANK2, PANK4, PAPSS1, PARP8, PCIF1, PEBP4, PELP1, PEX19, PGRMC2, PHF19, PI4KA, PIEZO1, PIGU, PLA2G2A, PNPLA2, PNPLA6, POLB, PPARD, PPFIBP2, PPIF, PPP1R21, PRDM10, PRDM8, PRKCD, PRKCQ, PRKX, PRMT2, PROCR, PRPSAP1, PSD4, PTHLH, PTK2B, PTPRN2, PXDC1, PYROXD2, QPCTL, RAB35, RACGAP1, RARRES2, RASA4, RBM12B, RBM41, RCC2, RD3, REEP4, REXO2, RFTN1, RIF1, RING1, RIPK4, RNF123, RNF224, RNF5, RNFT1, RPS6KA1, RSPH10B/RSPH10B2, RXRA, S100A2, SAMD11, SART3, SEC22A, SEPHS2, SEPTIN10,

For Review Only

	SETD1A, SF3A3, SFMBT1, SGSM3, SH2D2A, SHPRH, SHROOM3, SIPA1L2, SLA2, SLAIN1, SLC17A7, SLC1A7, SLC25A19, SLC25A33, SLC25A46, SLC2A13, SLC2A6, SLC2A8, SLC35A1, SLC37A2, SLC41A1, SLC43A2, SLC46A2, SLC50A1, SLC8A2, SLC9A3R1, SLCO4A1, SMC3, SMG5, SMOC1, SNX11, SNX33, SOCS2, SORD, SPATA31A6 (includes others), SPOP, SPSB2, SPTLC2, SRR, SSBP3, STK11IP, STKLD1, SUDS3, SULT1A3/SULT1A4, SURF4, SYNJ2, SYT2, SYTL4, TBC1D10B, TBC1D2, TBCE, TCERG1L, TCF20, TCFL5, TEP1, TET3, TK1, TLX2, TMCO4, TMEM159, TMEM181, TMEM182, TMEM233, TMEM260, TMEM39B, TMEM88, TMOD1, TOP3B, TRAF7, TRAPPC4, TRIM28, TRIM37, TRIP10, TRMT2B, TSC22D4, TSR2, TTC36, TTC7A, TXLNA, TXNRD2, UAP1L1, UBIAD1, UBQLN3, UCK1, UPF3B, URGCP, USP32, USP5, USP53, VAV2, VPS33B, VPS37D, WNT8B, WRAP73, XIRP1, XRCC4, YIPF2, ZDHHC3, ZFYVE21, ZNF154, ZNF276, ZNF341, ZNF653, ZNF721, ZNF75D, ZNF775, ZNF780A, ZNF784, ZNF81, ZNRF3, ZSWIM8
Gastrointestinal Disease	AAMP, ABCB7, ACADVL, ACLY, ACOT8, ACOXL, ACSF3, ACTN4, ADAMTS12, ADAMTS2, ADORA2B, ADPRH, AEBP1, AHNAK, AKAP8, ALG12, ANGPTL2, ANKRD13B, ANKS1A, AOC1, AP1G2, APAF1, APOBR, APOE, APTX, AQP12A/AQP12B, AQP2, ARC, ARFGAP1, ARFIP2, ARHGAP35, ARHGEF11, ARHGEF40, ARL6IP6, ARV1, ATE1, ATG14, ATP13A1, ATP1A1, ATP1A3, ATRAID, ATXN7L2, AWAT1, AZIN2, BBS7, BCAS1, BCORL1, BMS1, BST1, C11orf68, C16orf70, C22orf34, C6orf136, CACNA1B, CAD, CALML4, CAMK2N1, CAPG, CASP10, CASP8, CASR, CBY2, CCDC142, CCDC166, CCDC81, CCDC84, CCDC88B, CCNF, CCSAP, CD14, CD3E, CDH15, CDX4, CEACAM21, CHCHD4, CHKB, CHTOP, CLCN6, CLCNKB, CLDN23, CLN8, CMSS1, CMTM1, CNGA2, CNGB1, CNNM2, CNTROB, COG1, COG8, COL9A1, COQ7, COX18, CPLANE1, CRNN, CRYBA2, CTCF, CTSH, CTSV, CTTNBP2, CYP2S1, CYP4A22, CYREN, D2HGDH, DDIT4, DDX56, DEPDC7, DEPTOR, DHX33, DIPK1B, DLGAP1, DMAP1, DNAAF3, DNAJC16, DNMT3A, DPP6, DTNBP1, DUS3L, EEF1AKNMT, EEF1G, EGF, EIF4E2, ELMOD3, EMC9, EMD, EML4, EN2, ENTPD2, EPHB2, ESM1, ESPN, EXOC3L2, FABP5, FAM111A, FAM214B, FAM72C/FAM72D, FARSB, FBXL20, FBXO38, FEM1A, FILIP1L, FKBP2, FKBP5, FKBPL, FLI1, FNTB, FRAT2, GALNT12, GAS1, GAS7, GBA2, GBGT1, GEN1, GJA3, GLI1, GNA12, GNAI1, GNAT1, GNL1, GNL2, GPAT2, GPR141, GRHL2, GRK6, GTF2H4, GTPBP1, GTPBP2, GUCY1B1, H2BC10, HAMP, HAUS3, HBEGF, HDHD3, HEATR5A, HIBADH, HIF1AN, HK1, HK3, HNRNPCL1/HNRNPCL2, HNRNPF, HOMER2, HSCB, HSD17B10, HSPA8, HSPB6, HTRA1, HTRA2, HVCN1, IDH3G, IFFO1, IGF1R, IGF2BP1, IGSF21, IKBKE, IL1R2, IL1RL2, INO80C, INPP5D, INPPL1, INTS10, IRGQ, ISY1, ITGAL, IVNS1ABP, JADE3, JUN, KCNA7, KCNE3, KCNJ11, KCNJ15, KCTD13, KHNYN, KIAA0513, KIF1C, KLHDC3, KLHL6, KLK10, KLK6, KNOP1, KRT16, KRT38, LAMB4, LAT2, LCE1B, LCE2B, LDHC, LDHD, LDLRAP1, LEFTY2, LETMD1, LGALS2, LMF1, LOC102724159/PWP2, LOXHD1, LRRC3, LRRC47, LRRC57, LRRC8A, LRRFIP1, LSM14A, LUC7L, LUC7L2, MAN1A1, MAN2A2, MAP1LC3A, MAP3K10, MARCHF5, MBLAC1, MBTPS1, MCM5, MDN1, MED24, MIA, MICOS10-NBL1/NBL1, MLXIP, MOCS2, MPHOSPH8, MRAS, MRPL13, MRPL37, MYBL2, MYH11, MYH7, MYL1, MYL9, MYLIP, MYO15A, MYO1C, MYO5C, MYOZ3, NAA10, NAA40, NAGA, NDST1, NDUFAF7, NDUFS7, NEDD4L, NEK4, NFIC, NGB, NLRP12, NODAL, NPR1, NR2C2AP, NR4A2, NSD1, NSUN6, NTAN1, NUMA1, OAZ2, OGDH, OGG1, ORMDL3, OSGIN1, OTOP2, PANK2, PANK4, PAPSS1, PARP8, PCIF1, PDIK1L, PEBP4, PELP1, PEX19, PHF19, PI4KA, PIEZO1, PIGU, PLA2G2A, PNPLA2, PNPLA6, POLB, PPARD, PPFIBP2, PPP1R21, PRDM10, PRDM8, PRKCD,

For Review Only

	PRKCQ, PRKX, PRMT2, PRNP, PSD4, PTHLH, PTK2B, PTPRN2, PXDC1, PYROXD2, QPCTL, RAB11B, RAB35, RAB5B, RABL2A, RACGAP1, RALGPS1, RASA4, RBM12B, RBM41, RCC2, RD3, REXO2, RFTN1, RGP1, RIF1, RING1, RIPK4, RNF112, RNF123, RNF5, RNFT1, RPS6KA1, RXRA, SART3, SCAMP4, SEPHS2, SEPTIN10, SETD1A, SFMBT1, SGSM3, SH2D2A, SHPRH, SHROOM3, SIPA1L2, SLA2, SLAIN1, SLC17A7, SLC1A7, SLC25A11, SLC25A19, SLC25A33, SLC25A46, SLC2A13, SLC2A6, SLC2A8, SLC35A1, SLC37A2, SLC38A7, SLC41A1, SLC43A2, SLC46A2, SLC50A1, SLC8A2, SLC9A3R1, SLCO4A1, SMC3, SMG5, SMOC1, SNX24, SNX33, SOCS2, SOCS6, SORD, SOX14, SPATA31A6 (includes others), SPOP, SPTLC2, SSBP3, STK11IP, STKLD1, SUDS3, SURF4, SYNJ2, SYT2, SYTL4, TARBP2, TBC1D10B, TBC1D2, TBCE, TCERG1L, TCF20, TCFL5, TEP1, TET3, TK1, TLX2, TMCO4, TMEM159, TMEM181, TMEM182, TMEM211, TMEM233, TMEM260, TMEM39B, TMEM88, TNFRSF10D, TOP3B, TRAF7, TRAPPC4, TRIB3, TRIM28, TRIM37, TRIP10, TSC22D4, TSR2, TTC7A, TXLNA, TXNRD2, UAP1L1, UBIAD1, UBQLN3, UCK1, UCN2, UPF3B, USP21, USP32, USP5, USP53, VAMP1, VAV2, VPS33B, WDR55, WRAP73, XIRP1, XRCC4, YIPF2, YPEL1, ZBTB2, ZDHHC3, ZFYVE21, ZNF154, ZNF175, ZNF276, ZNF341, ZNF420, ZNF426, ZNF653, ZNF721, ZNF75D, ZNF775, ZNF780A, ZNF784, ZNRF3, ZSWIM8
Hematological Disease	ABCB7, ACADVL, ADAMTS12, ADORA2B, AHNAK, AKAP8, ANGPTL2, AP1G2, APAF1, APOE, ARHGAP35, ARHGEF39, ATP1A1, BBS7, BCAS1, BCL2L14, BCL7A, BCORL1, C6orf136, CAD, CALML4, CAMK2N1, CASP10, CASP8, CASR, CCNF, CD14, CD3E, CLCNKB, CLIC3, CLN8, CMTM1, CNNM2, CPLANE1, CRNN, CSAG2/CSAG3, CTCF, CTSH, CYP4A22, DHX33, DNMT3A, EGF, ELMOD3, ENTPD2, EPHB2, FAM111A, FBXO38, FILIP1L, FLI1, FNTB, GAS7, GEN1, GFI1, GLI1, GNA12, GNAI1, GPAT2, GRHL2, GTPBP2, HAMP, HDHD3, HK3, HNRNPCL1/HNRNPCL2, HTRA2, HVCN1, IGF1R, IGLC1, IKBKE, IL1RL2, INPP5D, JUN, KCNJ15, KHNYN, KLHDC3, KLHL6, KRT16, KRT38, KRT6C, LAMB4, LAT2, LCE4A, LRRC3, LRRC8A, LRRFIP1, LSM14A, LUC7L2, MCM5, MDN1, MED24, MRPL37, MSRB2, MYBL2, MYH11, MYL1, MYL9, MYLIP, MYO15A, MYO5C, NEDD4L, NEK4, NLRP12, NR4A2, NSD1, NUMA1, OGG1, OTOP2, PANK2, PANK4, PDIK1L, PEBP4, PFDN1, PLA2G2A, POLB, PPFIBP2, PRKCD, PRKCQ, PSMB6, PTHLH, PTK2B, RASA4, RBM41, RFTN1, RGP1, RIF1, RIPK4, RNF112, RNF123, RPS6KA1, RSPH10B/RSPH10B2, RXRA, SEPTIN10, SH2D2A, SHPRH, SHROOM3, SIPA1L2, SLC17A7, SMC3, SMOC1, SOCS2, SORD, SPATA31A6 (includes others), SULT1A3/SULT1A4, SYTL4, TARBP2, TBC1D2, TBCE, TCEAL3, TEP1, TET3, TMCO4, TNFRSF10D, TXLNA, UBQLN3, USP32, VAV2, XIRP1, XRCC4, ZNF276, ZNF341, ZNF784, ZNRF3, ZSWIM8

For Review Only

Supplementary Table S5. Transcripts assigned to the additional diseases and functions in Figure 5c. Comparison of monocytes incubated with plasma extracellular vesicles (EVs) from patients with rectal adenoma polyps (AP$_{EV}$) or invasive adenocarcinoma (RC$_{EV}$) who had either localized cancer (local-RC$_{EV}$) or metastatic disease (met-RC$_{EV}$). The data represent a *p*-value cut-off of 0.05 and fold-change cut-off of 1.2.

Diseases and functions	*p*-value range	ID
RC$_{EV}$ vs AP$_{EV}$		
Inflammatory Response	6.42E-03-3.90E-02	ATXN3, PLA2G4A, PLD1, RORC, TREM2
Immune Cell Trafficking	3.21E-03-2.54E-02	PLA2G4A, RORC
Cell Death and Survival	1.34E-03-3.48E-02	ATXN3, PIAS2, PLA2G4A, PLD1, PPARGC1A, PPIF, RORC
Gastrointestinal disease	1.56E-04-3.92E-02	ACTR3B, ANGEL2, ARIH2OS, ATXN3, CHST12, DCLRE1C, DDR2, DDX51, DERL2, DGKD, DPPA3, EFCAB6, EPS15, EZHIP, GAN, GART, GNB5, GOLIM4, GRIN3B, HCN4, IRX3, KBTBD12, KCNJ14, KCNMB3, LIN7A, LRRC3, MEAK7, MLLT10, NEB, NPHP3, PALM, PIAS2, PLA2G4A, PLD1, PNMA2, PPARGC1A, RASSF8, RFTN1, RORC, SCIN, SNX21, SPAG11B, TDRD3, TESK2, TM4SF1, TMEM185A, TPRG1, TREM2, TRIM62, TRIM63, TSPAN12, TXNDC5, USP1, USP32, USP54, VASN, VNN2, ZFAND1, ZFP62, ZNF785
met-RC$_{EV}$ vs local-RC$_{EV}$		
Inflammatory Response	1.38E-02-2.59E-02	ACLY, ACTN4, ADA2, ADGRE3, ADORA2B, AOC1, APAF1, APOE, BST1, CAPG, CASP8, CD14, COQ7, CTSH, CYP2S1, EGF, FABP5, GFI1, GRK6, GTPBP2, HBEGF, HK3, HSPA8, HVCN1, IGF1R, INPP5D, INPPL1, ITGAL, LAT2, LEFTY2, NODAL, ORMDL3, PFDN1, PPARD, PRKCD, PRNP, PTPRN2, RAB5B, RARRES2, RASA4, SLA2, SLC9A3R1, STK11IP, SURF4, SYTL4, TRIB3, TXLNA, VAV2
Immune Cell Trafficking	4.87E-03-2.59E-02	APOE, CASP8, CD3E, FABP5, GFI1, GNA12, INPP5D, ITGAL, LAT2, MRAS, PFDN1, PRKCD, PRKCQ, PTK2B, RARRES2, SLA2, TXLNA, VAV2
Cell Death and Survival	1.29E-03-2.59E-02	APAF1, APOE, ATP1A1, BCL2L14, CA3, CASP10, CASP8, CASR, CD3E, CERS5, COMMD4, CTCF, CTSV, DCAF1, DDIT4, EGF, ELOA, EMD, EPHB2, FKBPL, FLI1, GAS7, GLI1, GNA12, GPAT2, GRHL2, HBEGF, HK1, HSD17B10, HTRA1, HTRA2, IGF1R, IKBKE, IVNS1ABP, JUN, KLK6, MAP1LC3A, MBTPS1, MDN1, MYBL2, MYH11, NFIC, NR4A2, OSGIN1, PEBP4, PELP1, PNPLA6, POLB, PPARD, PRKCD, PRMT2, PRNP, PTHLH, PTK2B, RASA4, RNF5, RPS6KA1, RXRA, SLC25A11, SNX33, TNFRSF10D, TRAF7, TRIB3, TRIM28, TRIM37, TRIP10

For Review Only

Supplementary Figures

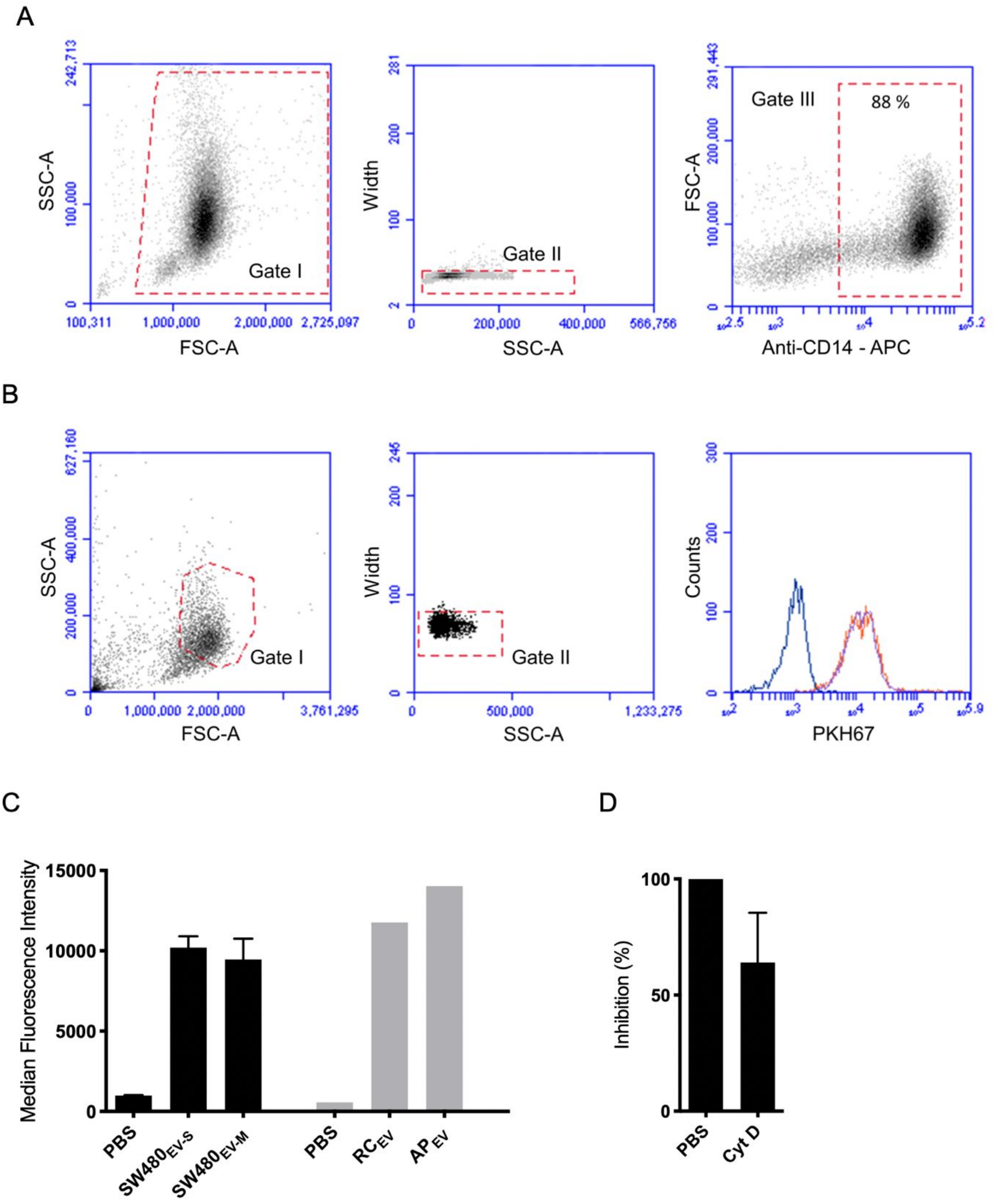

Supplementary Figure S1. Uptake of SW480-derived and plasma extracellular vesicles (EVs) by human primary monocytes. A Representative plots in flow cytometry analysis for identification of monocytes. Cells were selected in an SSC *versus* FSC dotplot (gate I) and single cells were identified and gated (gate II) in a width *versus* SSC dotplot. Single monocytes were identified (gate III) in a FSC *versus* Anti-CD14-APC dotplot. **B** Representative plots in flow cytometry analysis of primary monocytes incubated with PKH67-labeled EVs from the colorectal cancer cell line SW480. Monocytes were identified and gated (gate I) in an SSC *versus* FSC dotplot and single monocytes were identified and gated (gate II) in a width *versus* SSC dotplot. The fluorescence intensity of primary monocytes incubated with PKH67-labeled

EVs is shown in an overlay histogram of monocytes given PKH67-labeled phosphate-buffered saline (PBS) (dark blue line), $SW480_{EV\text{-}S}$ (violet line), or $SW480_{EV\text{-}M}$ (orange line). **C** Flow cytometry analysis of primary monocytes incubated with PKH67-labeled EVs. The data represent the mean ± standard deviation of median fluorescence intensity from 3 independent experiments for small EVs ($SW480_{EV\text{-}S}$) and medium EVs ($SW480_{EV\text{-}M}$) from the colorectal cancer SW480 cell line and 1 experiment each for plasma EVs from patients with either rectal adenocarcinoma (RC_{EV}) or a rectal adenoma polyp (AP_{EV}). Monocytes given PKH67-labeled PBS were used as negative control. **D** Flow cytometry analysis of primary monocytes incubated with PKH67-labeled $SW480_{EV\text{-}S}$ in the absence (PBS) or presence of cytochalasin D (Cyt D). The data represent mean percent inhibition ± standard deviation from 3 independent experiments.

For Review Only

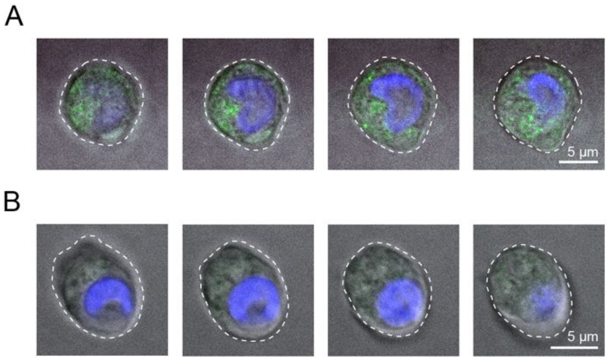

Supplementary Figure S2. Internalization of SW480-derived small extracellular vesicles (EVs) by human primary monocytes. Single z-planes of the monocyte shown in each lower panel of Figure 3 A and B. **A** Images of monocyte given PKH67-labeled small EVs (green). **B** Corresponding images of monocytes incubated with PKH67-labeled phosphate-buffered saline as negative control. **For all panels** Cells were fixed and mounted with the nuclear stain 4',6-diamidino-2-phenylindole (DAPI; blue). White dashed lines mark the plasma membrane, which is also shown in the transmitted light channel (grey). Scale bars are 5 µm.

For Review Only

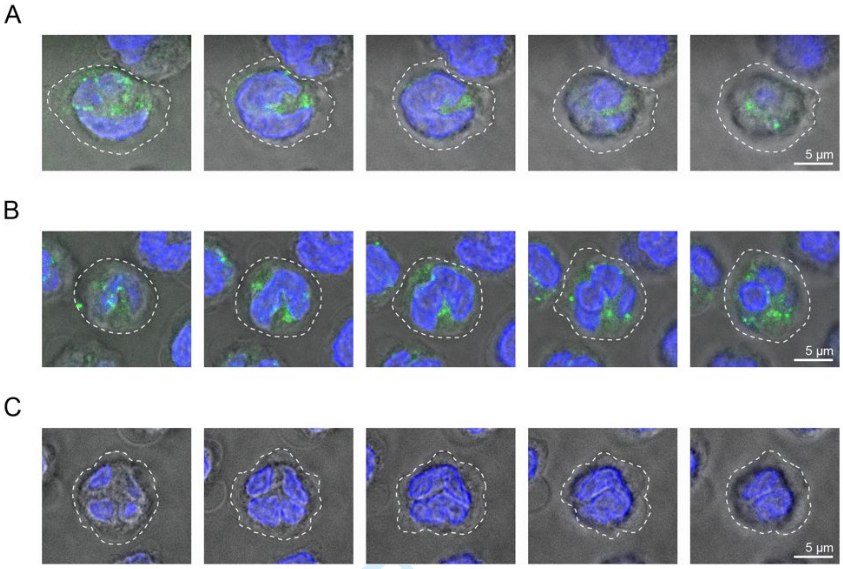

Supplementary Figure S3. Internalization of plasma extracellular vesicles (EVs) by human primary monocytes. Single z-planes of the cell shown in each lower panel of Figure 4 A-C. **A** Images of monocytes given PKH67-labeled EVs (green) from a patient with rectal adenocarcinoma. **B** Corresponding images of monocytes incubated with PKH67-labeled EVs (green) from a patient with a rectal adenoma polyp. **C** Corresponding images of monocytes incubated with PKH67-labeled phosphate-buffered saline as negative control. **For all panels** Cells were fixed and mounted with the nuclear stain 4',6-diamidino-2-phenylindole (DAPI; blue). White dashed lines mark the plasma membrane, which is also shown in the transmitted light channel (grey). Scale bars are 5 µm.

For Review Only

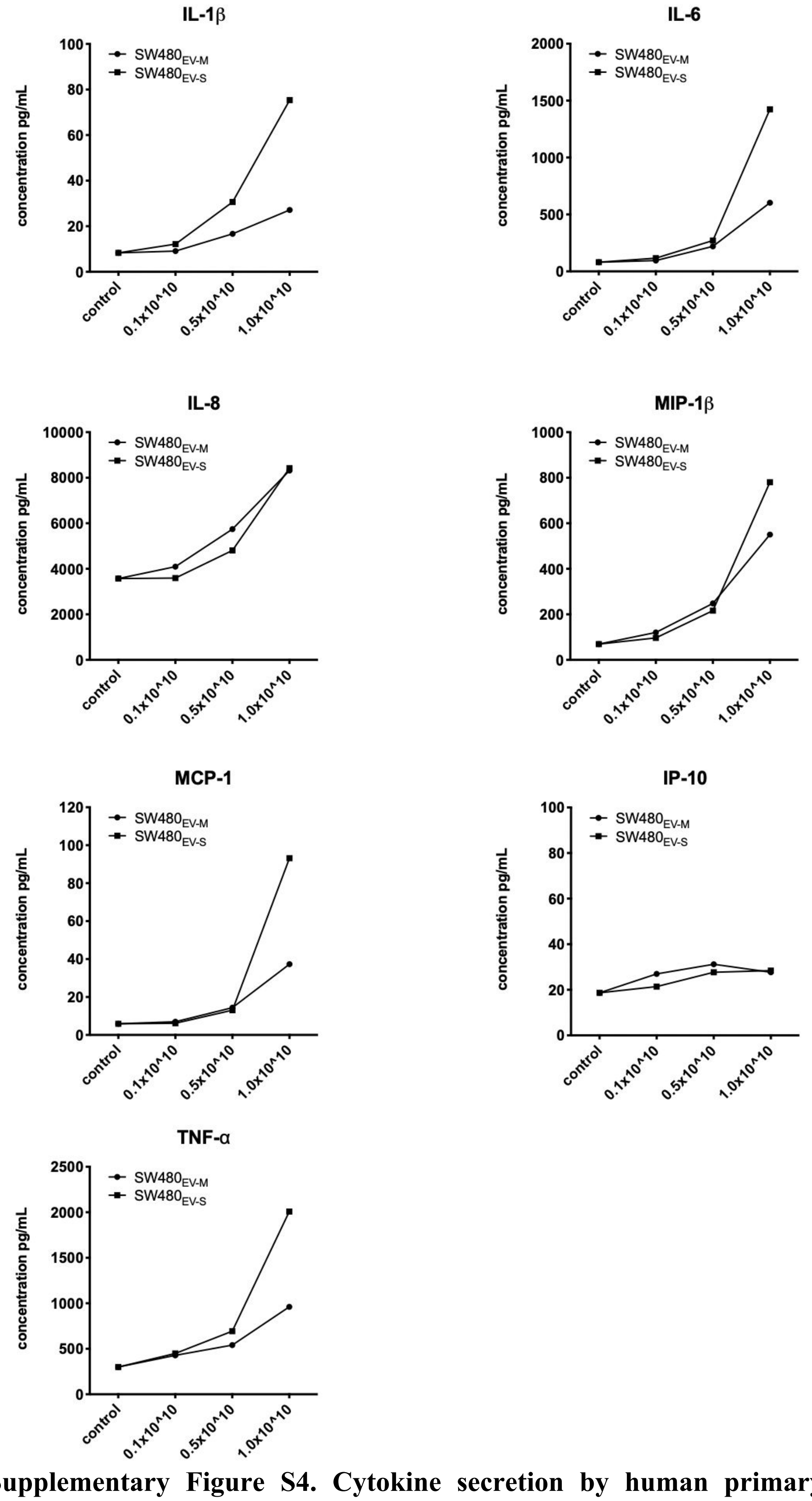

Supplementary Figure S4. Cytokine secretion by human primary monocytes given SW480-derived extracellular vesicles. Multiprotein (Luminex) assay results following incubation with colorectal cancer SW480 cell line medium EVs ($SW480_{EV\text{-}M}$) or small EVs ($SW480_{EV\text{-}S}$) in the indicated numbers (particles/ml). Control monocytes were given serum-free RPMI medium. The data represent an experiment performed once.
